# Asiatic Acid from *Centella asiatica* as a Potent EGFR Tyrosine Kinase Inhibitor with Anticancer Activity in NSCLC Cells Harboring Wild-Type and T790M-Mutated EGFR

**DOI:** 10.3390/biom15101410

**Published:** 2025-10-03

**Authors:** Chaiwat Monmai, Sahachai Sabuakham, Wachirachai Pabuprapap, Waraluck Chaichompoo, Apichart Suksamrarn, Panupong Mahalapbutr

**Affiliations:** 1 Department of Biochemistry, and Center for Translational Medicine, Faculty of Medicine, Khon Kaen University, Khon Kaen 40002, Thailand; bbuayy@gmail.com; 2 School of Biomolecular Science and Engineering, Vidyasirimedhi Institute of Science and Technology, Rayong 21210, Thailand; sahachai.s@vistec.ac.th; 3 Department of Chemistry, and Center of Excellence for Innovation in Chemistry, Faculty of Science, Ramkhamhaeng University, Bangkok 10240, Thailand; wachirachai7@gmail.com (W.P.); waraluck_kik@hotmail.com (W.C.); asuksamrarn@yahoo.com (A.S.)

**Keywords:** EGFR, *Centella asiatica*, pentacyclic triterpenoid, anticancer, NSCLC, molecular docking

## Abstract

Lung cancer is a leading cause of cancer mortality worldwide. Targeted therapies with epidermal growth factor receptor (EGFR) tyrosine kinase inhibitors (TKIs) represent a significant advance in the management of lung cancer. However, their long-term efficacy is often limited by acquired resistance, particularly due to the T790M mutation, highlighting the need for novel EGFR-TKIs. Although compounds derived from *Centella asiatica* have demonstrated anticancer potential, their role in EGFR inhibition has not yet been reported. In this study, we investigated the inhibitory activity of two primary constituents, asiaticoside and asiatic acid, against wild-type and double-mutant (L858R/T790M) EGFR, as well as the anticancer effects of the more potent compound in lung cancer cells. A kinase activity assay revealed that asiatic acid potently inhibited both wild-type and double-mutant EGFR, whereas asiaticoside showed minimal inhibitory activity. Molecular docking demonstrated that asiatic acid bound to the ATP-binding pocket of both EGFR forms with binding energies superior to those of erlotinib and osimertinib. Treatment with asiatic acid significantly (i) reduced viability of A549 and H1975 cells while remaining non-toxic to BEAS-2B normal lung cells, (ii) enhanced cancer cell apoptosis, (iii) suppressed extracellular signal-regulated kinase (ERK) and protein kinase B (Akt) signaling pathways, and (iv) inhibited EGFR activation in A549 and H1975 cells. These results suggest that asiatic acid is a promising lead compound for anticancer drug development.

## 1. Introduction

Cancer, a multifaceted and devastating group of diseases, poses a major threat to global public health. It is characterized by the unchecked growth and spread of abnormal cells, which disrupt normal tissue function and can be fatal if left untreated [1,2]. The scale of the problem is staggering: nearly 20 million new cases and about 10 million deaths are reported each year, and these figures are projected to rise, underscoring the urgent need for more effective prevention, diagnosis, and treatment strategies [3]. Among these, lung cancer remains a major global health challenge and one of the leading causes of cancer-related mortality worldwide [4]. In 2020, it accounted for approximately 2.21 million new cases and 1.80 million deaths, making it the second most common cancer [5,6].

Epidermal growth factor receptor (EGFR) plays a crucial role in regulating cell proliferation, differentiation, and survival [7]. Autophosphorylation of EGFR triggers multiple downstream signaling pathways, including mitogen-activated protein kinase/extracellular signal-regulated kinase (MAPK/ERK), phosphatidylinositol 3-kinase/Akt (PI3K/Akt), and Janus kinase/signal transducer and activator of transcription (JAK/STAT) [8]. Alterations in EGFR—such as overexpression, exon 19 deletions, and the L858R substitution in exon 21—are key oncogenic drivers in non-small cell lung cancer (NSCLC) [9]. Erlotinib, a first-generation EGFR tyrosine kinase inhibitor (EGFR-TKI), provides clinical benefits to NSCLC patients [10]. However, its long-term efficacy is often limited by acquired resistance, particularly due to the T790M mutation [11]. Consequently, the development of novel EGFR-TKIs remains a critical need.

**Figure 1:**
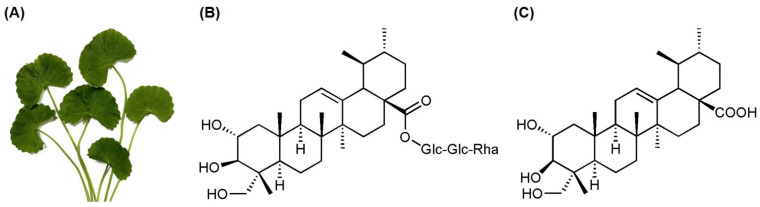
(**A**) *Centella asiatica* and the chemical structures of its two major isolated compounds: (**B**) asiaticoside and (**C**) asiatic acid.

*Centella asiatica* (Figure 1A) is widely used as a medicinal herb across Asia, Europe, Australia, the Americas, and Southern Africa [12]. It exhibits a wide range of pharmaceutical potentials, including treatment of various skin conditions [13], promotion of wound healing [14], antioxidant [15], anti-inflammatory [16], and anti-apoptotic [17] activities. The primary active constituents of *C. asiatica* are pentacyclic triterpenes [18], including asiaticoside [19], asiatic acid [20,21,22], madecassoside [23], and madecassic acid [24], which have demonstrated anticancer potential. Although the anticancer effects of *C. asiatica*’s active compounds have been studied, their targeted inhibition of EGFR (both wild-type and T790M mutant) has not yet been reported. In this study, we evaluated the inhibitory activity of two major constituents, asiaticoside and asiatic acid, against both wild-type and double-mutant (L858R/T790M) EGFR, and further examined the anticancer effects of the more potent compound in human NSCLC cell lines expressing wild-type EGFR (A549) and double-mutant EGFR (H1975).

## 2. Materials and Methods

### 2.1. Extraction and Isolation of Asiaticoside and Asiatic Acid

*Centella asiatica* (Apiaceae) was collected from Nonthaburi Province, Thailand, in 2022. The plant species was identified by Mr. Yanyong Punpreuk, Department of Agriculture, Bangkok, Thailand, and a voucher specimen was deposited at the Faculty of Science, Ramkhamhaeng University (Apichart Suksamrarn, No. 102). Asiaticoside and asiatic acid were isolated from the aerial parts of this plant species. Briefly, the fresh aerial parts of *C. asiatica* (1.3 kg) were washed, air-dried, milled, and macerated with MeOH. The filtered solution was evaporated under reduced pressure at 40–45 °C to yield the MeOH extract (75.8 g). The crude extract was subjected to column chromatography on silica gel using a CH_2_Cl_2_–MeOH gradient system, yielding five fractions. Fraction 2 was chromatographed on silica gel column (CH_2_Cl_2_–MeOH, 95:5) to yield asiatic acid (28.5 mg, 0.04% yield). Fraction 4 was purified by reverse phase-C_18_ column chromatography (MeOH–H_2_O, 1:4) to afford asiaticoside (142.8 mg, 0.19% yield). The structures of asiaticoside and asiatic acid were confirmed by comparing their spectroscopic data (^1^H, ^13^C NMR, and mass spectra; Figures S1–S6) with those previously reported in the literature ([25] for asiaticoside and [26] for asiatic acid). The purity level of the isolated compounds is greater than 95% from ^1^H NMR and TLC investigations. Compounds with high molecular weight typically exhibit poor solubility, which can be attributed to their stronger intermolecular interactions, greater chain entanglement, and larger non-polar surface area [27]. Therefore, the stock solutions of asiaticoside and asiatic acid were prepared at a concentration of 50 mM in 100% DMSO.

### 2.2. EGFR Inhibitory Activity of Asiaticoside and Asiatic Acid

The EGFR inhibitory activity of the tested compound was measured using the ADP-Glo^TM^ assay (Promega, WI, USA) according to a previous study [28]. Kinase reactions were performed in a 384-well plate and contained 2 μL of the tested compound, 8 μL of 1X kinase buffer, 25 μM ATP, 2.5 μM Poly(Glu-Tyr), and EGFR enzyme [1.25 ng/μL; wild-type (SRP0239), or L858R/T790M (SRP0242); Sigma-Aldrich, St. Louis, MO, USA]. For the positive control, 2 μL of DMSO was added in place of the tested compound. For the negative control, the EGFR enzyme was omitted and replaced with 5 μL of 1X kinase buffer. The plate was protected from light and incubated at room temperature for 1 h, followed by the addition of 5 μL ADP-Glo^TM^ reagent and a further 40-min incubation. To convert ADP to ATP, 10 μL of kinase detection reagent was added, followed by a 30-min incubation under the same conditions. ATP levels were then measured using a Varioskan LUX multimode microplate reader (Thermo Fisher Scientific, Waltham, MA, USA), and percent inhibition was calculated using Equation (1).

(1) $$\mathrm{Inhibition}\;\mathrm{activity}\;\left(\%\right)=\left[\frac{\left(\mathrm{positive}\;-\;\mathrm{negative}\right)-\left(\mathrm{sample}\;-\mathrm{negative}\right)}{\mathrm{positive}-\mathrm{negative}}\right]\;\times 100$$

The IC_50_ of each compound was calculated using GraphPad Prism software (version 8.0.2, GraphPad Software, Inc., Boston, MA, USA).

### 2.3. Preparation of Structure and Molecular Docking

The binding interactions of the studied compound with wild-type and double-mutant EGFR were evaluated using molecular docking. The crystal structures of EGFR were retrieved from the Protein Data Bank (PDB), using PDB entry 1M17 for wild-type EGFR and 4I22 for the double-mutant (L858R/T790M) EGFR. The three-dimensional (3D) structure of the tested compound was constructed using the GaussView 6.0 program (Gaussian, Inc., Wallingford, CT, USA). Protonation states of the protein and ligand at physiological pH (7.4) were assigned using the APBS webserver (https://server.poissonboltzmann.org, accessed on 7 August 2025) and MarvinSketch (version: 24.3.0: Chemaxon Ltd., Budapest, Hungary), respectively. The optimized protein and ligand structures were converted to pdbqt format using AutoDockTools (version 1.5.7; Molecular Graphics Laboratory, The Scripps Research Institute, La Jolla, CA, USA). The grid box dimensions and centers were defined as follows: for wild-type EGFR, the grid size was set to 50 Å × 50 Å × 60 Å with center coordinates at x = 25.32, y = −0.99, and z = 53.94; for double-mutant EGFR, the grid size was 50 Å × 50 Å × 50 Å with center coordinates at x = 10.66, y = −15.55, and z = 8.89. Molecular docking was performed using AutoDock version 4.2.6, and the docking energies (kcal/mol) of the resulting protein-ligand complexes were obtained using a Lamarckian genetic algorithm and an empirical binding free energy function. To validate the docking protocol, the co-crystallized ligand was re-docked, yielding RMSD values of 1.69 Å for wild-type EGFR and 1.25 Å for mutant EGFR (Figure S7), confirming the accuracy of the procedure. Two-dimensional (2D) interaction profiles of the studied compounds with their target proteins were generated using Discovery Studio Visualizer (version 21.1.0.20298; BIOVIA, San Diego, CA, USA).

### 2.4. Cell Lines and Cell Culture

BEAS-2B, A549, and H1975 cell lines were purchased from the American Type Culture Collection (ATCC; Manassas, VA, USA). All cancer cell lines (A549 and H1975) were maintained in Dulbecco’s Modified Eagle’s Medium (DMEM; Gibco, Waltham, MA, USA) supplemented with 10% (*v*/*v*) fetal bovine serum (FBS) and 1% (*v*/*v*) antibiotic-antimycotic (penicillin, streptomycin, and amphotericin B; Gibco, Brooklyn, NY, USA), at 37 °C in a 5% CO_2_ humidified incubator. BEAS-2B cells (a non-cancerous human bronchial epithelium) were maintained in Airway Epithelial Cell Medium (AECM; PCS-300-030, ATCC) supplemented with the Bronchial Epithelial Cell Growth Kit (PCS-300-040, ATCC) under identical incubation conditions.

### 2.5. Cell Viability Assay

Cells were seeded in a 96-well plate at 1,500 cells/well and incubated at 37 °C for 24 h. After incubation, the culture medium was replaced with fresh medium containing various concentrations of asiatic acid. Cells treated with 0.8% (*v*/*v*) DMSO served as the vehicle control. After 72 h, cells were incubated with 100 μL of MTT solution (0.5 mg/mL in PBS) for 4 h at 37 °C. The solution was then removed, 100 μL of DMSO was added, and the plate was protected from light with gentle agitation for 15 min. Absorbance was recorded at 540 nm using a Sunrise microplate reader (Tecan Group Ltd., Männedorf, Switzerland).

### 2.6. Cell Apoptosis Assay

Cells were plated in a 6-well plate at a density of 200,000 cells/well and allowed to adhere overnight at 37 °C. The following day, cells were treated with increasing concentrations of asiatic acid for 24 h. Erlotinib (10 μM) [29,30] and osimertinib at 5 μM (Table S1) were used as reference drugs for A549 and H1975 cells, respectively. After treatment, cells were harvested by trypsinization and centrifugation. The cell pellets were stained with FITC-conjugated Annexin V (640945; BioLegend, San Diego, CA, USA) and incubated for 30 min at room temperature in the dark. Propidium iodide (PI; P3566; Invitrogen^TM^, Waltham, MA, USA) was then added, and apoptotic populations were analyzed using a BD FACSCalibur flow cytometer (BD Biosciences, Heidelberg, Germany).

### 2.7. Western Blot Analysis

Cells were plated at 200,000 cells/well in a 6-well plate and allowed to adhere for 24 h. They were then treated with the tested compound at varying concentrations for 24 h. For A549 cells, treatment was performed in serum-free medium for 24 h, followed by stimulation with 100 ng/mL human EGF for 10 min prior to harvesting [31,32]. Cells were harvested by trypsinization, lysed in RIPA buffer [50 mM Tris-HCl (pH 7.4), 150 mM NaCl, 1% NP-40, 1% Na-deoxycholate, and 0.1% SDS] with protease and phosphatase inhibitors, and incubated on ice for 30 min. Lysates were centrifuged, and protein concentration was determined using the Bradford assay. Equal amounts (30 μg) were separated by 10% SDS-PAGE and transferred to a nitrocellulose membrane (Immobilon^®^-P, Merck Millipore, Burlington, MA, USA). The membrane was blocked with 5% skim milk in 1X TBST and incubated overnight at 4 °C with primary antibodies (Table S2) diluted 1:1000 in 1% skim milk-TBST or 1% BSA-TBST. After washing, membranes were incubated at room temperature for 1 h with secondary antibodies—goat anti-Rabbit IgG or goat anti-Mouse IgG (Invitrogen, Waltham, MA, USA)—diluted 1:2000. Protein signals were detected using Luminata^TM^ Crescendo Western HRP Substrate (Merck Millipore) and imaged with an ImageQuant 800 system (GE Healthcare Bio-Sciences, Uppsala, Sweden). The auto exposure mode was selected to automatically adjust the exposure time to prevent image saturation. The detected signal was quantified using Image Studio Lite (version 5.2, LICORbio^TM^, Lincoln, NE, USA).

### 2.8. Statistic Analysis

Data are presented as mean ± standard error of the mean (SEM) from three independent experiments. Statistical differences between groups were assessed using one-way ANOVA followed by Tukey–Kramer post hoc test. Differences with *p* < 0.05 were considered statistically significant.

## 3. Results

### 3.1. Inhibitory Effect of Asiaticoside and Asiatic Acid on the EGFR Tyrosine Kinase Activity

To determine the EGFR inhibitory activity of the two major compounds isolated from *C. asiatica*, the ADP-Glo^TM^ assay was performed. Erlotinib is a reversible EGFR-TKI that blocks receptor activity by occupying the ATP-binding pocket of the kinase domain [33]. In contrast, osimertinib is a third-generation, irreversible EGFR-TKI that selectively targets the T790M resistance mutation by covalently binding to cysteine 797 within the ATP-binding pocket of the mutant receptor [34]. Accordingly, erlotinib and osimertinib were used as positive controls for wild-type and T790M-mutant EGFR, respectively.

**Figure 2:**
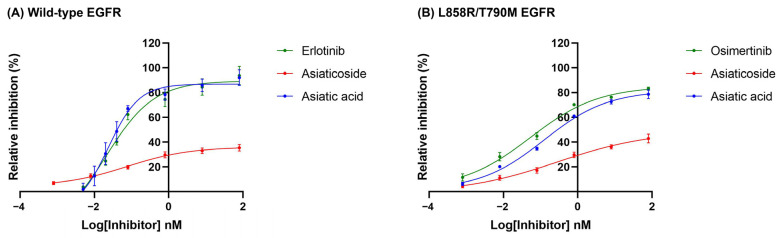
Kinase inhibitory activity against (**A**) wild-type EGFR and (**B**) double-mutant EGFR of asiaticoside and asiatic acid, as well as the known EGFR-TKIs, erlotinib and osimertinib. Data are shown as mean ± SEM (*n* = 3).

As shown in Figure 2, asiaticoside, asiatic acid, and the known EGFR-TKIs inhibited the kinase activity of both wild-type and double-mutant (T790M/L858R) forms of EGFR in a concentration-dependent manner. The IC_50_ values for kinase inhibition against wild-type EGFR were as follows: erlotinib, 0.062 ± 0.009 nM; asiaticoside, >80 nM; and asiatic acid, 0.035 ± 0.002 nM. Asiatic acid exhibited the lowest IC_50_ value, indicating the highest EGFR kinase inhibitory activity. For the double-mutant EGFR, the IC_50_ values were: osimertinib, 0.116 ± 0.038 nM; asiaticoside, >80 nM; and asiatic acid, 0.348 ± 0.055 nM (Table 1). Based on these results, only asiatic acid was chosen for further experiments due to its stronger inhibitory activity against both wild-type and mutant EGFR compared to asiaticoside.

The inhibitory effect of asiatic acid on fibroblast growth factor receptor (FGFR) was further examined to evaluate its selectivity. Results showed that asiatic acid inhibited FGFR kinase activity, with an IC_50_ of 0.471 nM (Figure S8). Overall, asiatic acid exhibits highly potent EGFR kinase inhibition and demonstrates approximately 13.5-fold greater selectivity for EGFR over FGFR.

### 3.2. Molecular Docking of Asiatic Acid into the Wild-Type and Double-Mutant Forms of EGFR

**Figure 3:**
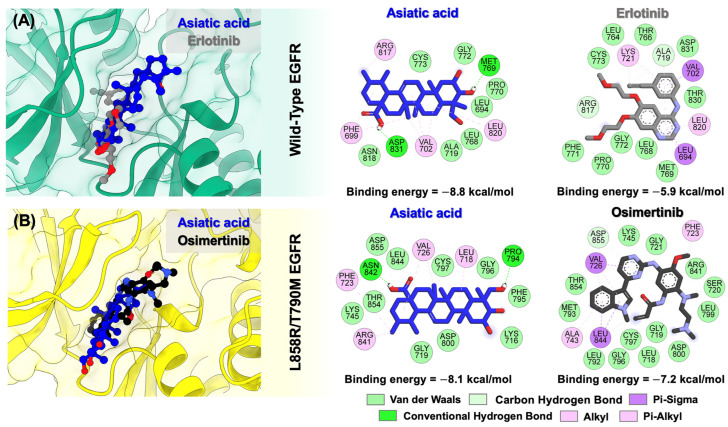
Superimposition (left) and 2D interaction profiles (right) of asiatic acid and the known EGFR-TKIs in complexes with (**A**) wild-type EGFR and (**B**) L858R/T790M double-mutant EGFR.

To elucidate how asiatic acid interacts with wild-type and mutant EGFR, molecular docking was performed. As shown in Figure 3, superimposition revealed that asiatic acid bound to the ATP-binding pocket of both wild-type EGFR and L858R/T790M EGFR similar to the known drugs, erlotinib and osimertinib. For wild-type EGFR, asiatic acid and erlotinib formed complexes with binding energies of −8.8 and −5.9 kcal/mol, respectively, whereas in the L858R/T790M mutant EGFR, asiatic acid and osimertinib bound with energies of −8.1 and −7.2 kcal/mol, respectively.

The complexation between asiatic acid and the wild-type EGFR was primarily stabilized by van der Waals and alkyl/pi-alkyl interactions with LEU694, PHE699, VAL702, ALA719, LEU768, PRO770, GLY772, CYS773, ARG817, ASN818, and LEU820 residues. Moreover, asiatic acid formed two hydrogen bonds with MET769 and ASP831 residues. Similarly, erlotinib binding to wild-type EGFR was mainly stabilized through van der Waals and pi interactions. Notably, several residues—LEU694, VAL702, ALA719, LEU768, MET769, PRO770, GLY772, CYS773, ARG817, LEU820, and ASP831—were common to both the asiatic acid and erlotinib complexes.

For double-mutant EGFR, asiatic acid formed several van der Waals interactions with LYS716, GLY719, LYS745, PHE795, GLY796, CYS797, ASP800, LEU844, THR854, and ASP855; alkyl/pi-alkyl interactions with LEU718, PHE723, VAL726, and ARG841; and hydrogen bonds with PRO794 and ASN842. Similarly, osimertinib binding to double-mutant EGFR was mainly stabilized through van der Waals and pi interactions. However, hydrogen bond formation was not observed in the osimertinib complex. The amino acid residues commonly involved in both asiatic acid–L858R/T790M EGFR and osimertinib–L858R/T790M EGFR complexes include LEU718, GLY719, PHE723, VAL726, LYS745, Gly796, CYS797, ASP800, ARG841, LEU844, THR854, and ASP855.

### 3.3. Effect of Asiatic Acid on the Cell Viability

**Figure 4:**
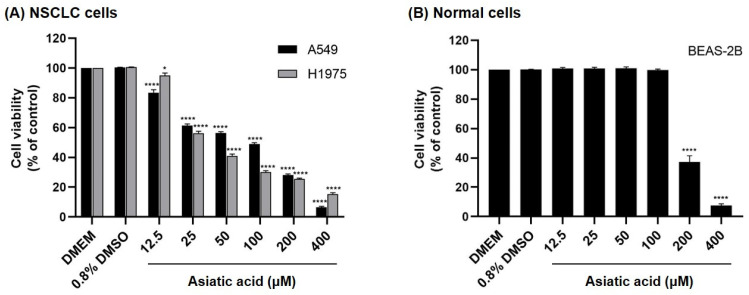
Effect of asiatic acid on cell viability of (**A**) A549 and H1975 NSCLC cells and (**B**) BEAS-2B normal lung epithelial cells. Data are shown as mean ± SEM (*n* = 3). Statistical analysis was performed using one-way ANOVA followed by Tukey–Kramer’s post hoc test. * *p* < 0.05 and **** *p* < 0.0001 vs. DMSO-treated group.

The cytotoxicity of asiatic acid was assessed in A549 (expressing wild-type EGFR) and H1975 (expressing L858R/T790M EGFR) [35] NSCLC cells using the MTT assay. As shown in Figure 4, asiatic acid suppressed cell viability in a concentration-dependent manner and exhibited strong cytotoxicity against A549 and H1975 cells, with IC_50_ values of 64.52 ± 2.49 μM and 36.55 ± 0.86 μM, respectively (Table 2). In contrast, asiatic acid displayed low cytotoxicity in non-cancerous BEAS-2B lung epithelial cells, with significant effects only at concentrations >100 μM (IC_50_ = 184.10 ± 5.38 μM), while concentrations ≤100 μM showed no notable cytotoxicity.

Compared with A549 cells, H1975 cells showed a more pronounced inhibitory response, in which asiatic acid treatment at concentrations of ≥100 μM reduced cell viability to below 40%. Based on these findings, concentrations of up to 100 μM for A549 cells and 50 μM for H1975 cells were selected for subsequent experiments.

### 3.4. Effect of Asiatic Acid on the A549 and H1975 Cell Apoptosis

**Figure 5:**
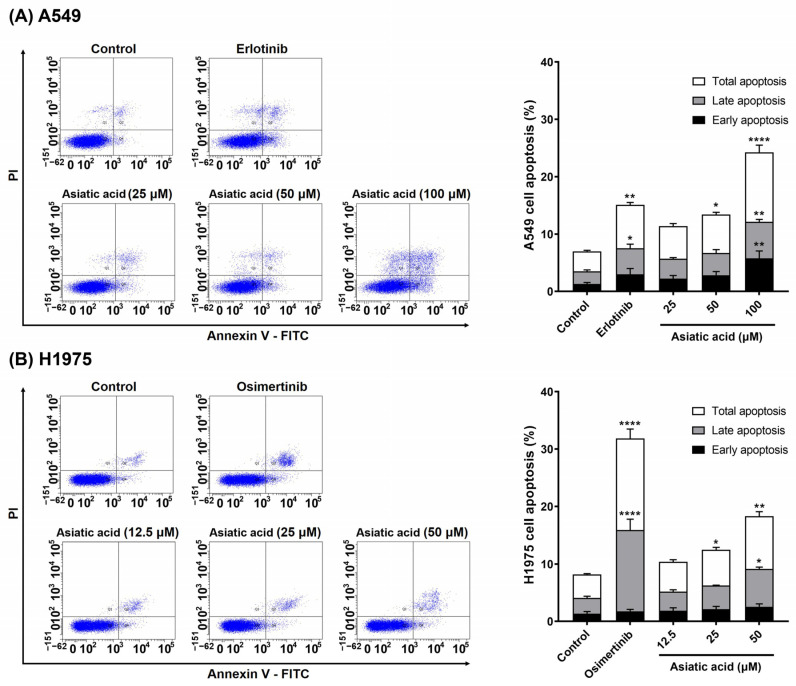
Analysis of apoptosis by flow cytometry. Asiatic acid induced apoptosis in (**A**) A549 and (**B**) H1975 NSCLC cells. DMSO was used as the vehicle control at 0.2% for A549 cells and 0.1% for H1975 cells. Erlotinib was used at a concentration of 10 μM, while osimertinib was used at 5 μM. Data are shown as mean ± SEM (*n* = 3). Statistical analysis was performed using one-way ANOVA followed by Tukey–Kramer’s post hoc test. * *p* < 0.05, ** *p* < 0.01, and **** *p* < 0.0001 vs. control.

To evaluate the role of apoptosis in asiatic acid-mediated cytotoxicity in NSCLC cells, Annexin V/PI staining was analyzed by flow cytometry. As shown in Figure 5, asiatic acid treatment significantly increased apoptosis in both A549 and H1975 cells compared to the vehicle control. At the highest tested concentrations, the apoptotic cell population increased approximately threefold in A549 cells (100 μM) and about twofold in H1975 cells (50 μM) relative to the DMSO-treated group. In A549 cells, 100 μM asiatic acid significantly induced both early (*p* = 0.0093) and late apoptosis (*p* = 0.0040), while in H1975 cells, 50 μM asiatic acid predominantly triggered late-stage (*p* = 0.0316) apoptosis. For known EGFR-TKIs, erlotinib treatment (10 μM) increased apoptosis approximately twofold in A549 cells, while osimertinib (5 μM) led to a threefold increase in apoptosis in H1975 cells compared to the untreated group.

**Figure 6:**
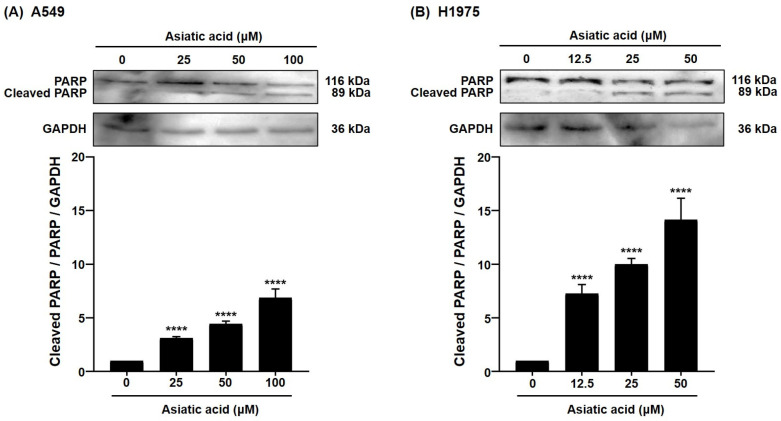
Analysis of cleaved PARP expression by Western blot. Asiatic acid induced cleaved PARP activation in both (**A**) A549 and (**B**) H1975 NSCLC cells. Data are presented as mean ± SEM (*n* = 3). Statistical analysis was performed using one-way ANOVA followed by Tukey–Kramer’s post hoc test. **** *p* < 0.0001 vs. 0 μM. Original western blot images can be found in Supplementary Materials.

The expression of cleaved poly(ADP-ribose) polymerase (PARP), a hallmark of programmed cell death, was further examined in A549 and H1975 cells to confirm the apoptosis-inducing activity of asiatic acid in NSCLC cells. As shown in Figure 6, treatment with asiatic acid led to a significant, concentration-dependent increase in cleaved PARP (Asp214) levels in A549 cells (25–100 μM, *p* < 0.0001). Similarly, in H1975 cells, cleaved PARP expression was significantly elevated following asiatic acid treatment (12.5–50 μM, *p* < 0.0001). These findings indicate that asiatic acid promotes PARP cleavage in both cell lines, consistent with its apoptosis-inducing activity (Figure 5).

### 3.5. Effect of Asiatic Acid on EGFR Downstream Signaling Pathways

**Figure 7:**
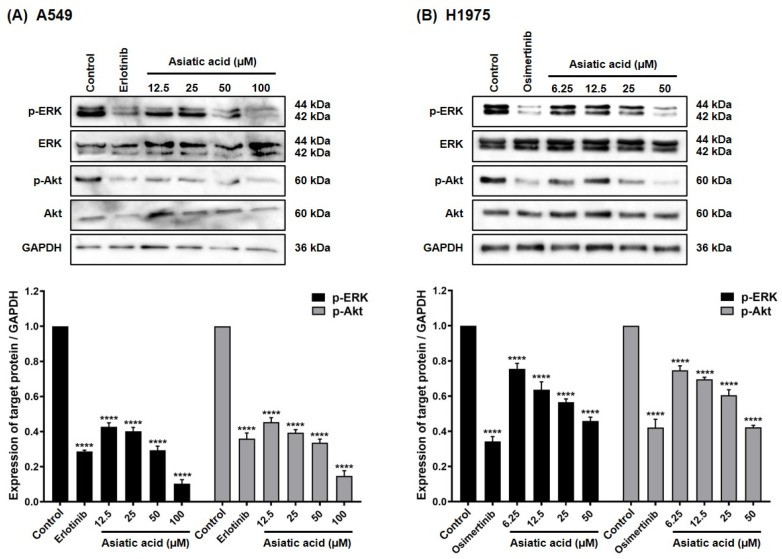
Analysis of p-ERK and p-Akt expression levels by Western blot. Asiatic acid suppressed ERK and Akt signaling pathways in (**A**) A549 and (**B**) H1975 NSCLC cells. DMSO was used as the vehicle control at 0.2% and 0.1% (*v*/*v*) for A549 and H1975 cells, respectively. Erlotinib (10 μM) and osimertinib (5 μM) served as reference treatments. Data are shown as mean ± SEM (*n* = 3). Statistical analysis was performed using one-way ANOVA followed by Tukey–Kramer’s post hoc test. **** *p* < 0.0001 vs. control. Original western blot images can be found in Supplementary Materials.

Western blot analysis was conducted to examine the effect of asiatic acid on EGFR-mediated survival pathways. As shown in Figure 7, phosphorylated ERK and Akt [p-ERK (Thr202/Tyr204) and p-Akt (Ser473)] were detected in both A549 and H1975 cells. Treatment with erlotinib (10 μM) and osimertinib (5 μM) significantly (*p* < 0.0001) reduced p-ERK and p-Akt levels in A549 and H1975 cells, respectively. In A549 cells, asiatic acid significantly decreased p-ERK and p-Akt levels compared to the vehicle control, with 100 μM treatment reducing these proteins more than erlotinib. Similarly, in H1975 cells, asiatic acid caused a significant, dose-dependent decrease in p-ERK and p-Akt (*p* < 0.0001). These results demonstrate that asiatic acid inhibits ERK and Akt signaling pathways in both NSCLC cell lines.

### 3.6. Effect of Asiatic Acid on EGFR Activation in A549 and H1975 Cells

**Figure 8:**
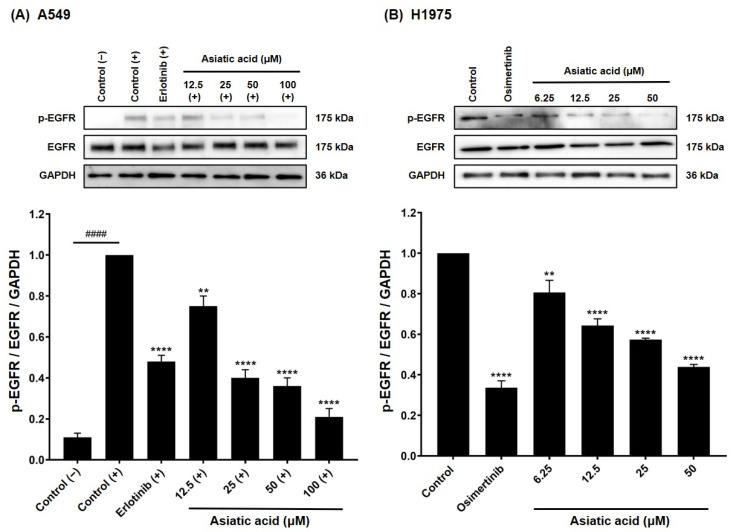
Analysis of p-EGFR expression by Western blot. Asiatic acid treatment significantly reduced p-EGFR levels in (**A**) A549 and (**B**) H1975 NSCLC cells. DMSO served as the vehicle control at 0.2% (*v*/*v*) for A549 cells and 0.1% (*v*/*v*) for H1975 cells. Erlotinib (10 μM) and osimertinib (5 μM) were used as reference treatments. (−) indicates no EGF stimulation, while (+) denotes EGF stimulation. Data are presented as mean ± SEM (*n* = 3). Statistical analysis was performed using one-way ANOVA followed by Tukey–Kramer’s post hoc test. ** *p* < 0.01 and **** *p* < 0.0001 vs. control/control (+). ^####^ *p* < 0.0001 vs. control (−). Original western blot images can be found in Supplementary Materials.

To assess whether asiatic acid–induced reductions in p-ERK and p-Akt were mediated through EGFR inhibition, the levels of p-EGFR (Tyr1068) were examined using Western blot. In A549 cells, basal p-EGFR expression was initially low (Figure 8A). Stimulation with human EGF increased p-EGFR levels, confirming activation of the EGFR. Treatment with 10 μM erlotinib significantly reduced EGF-induced EGFR phosphorylation compared with the EGF-stimulated group (*p* < 0.0001). Likewise, asiatic acid significantly inhibited EGF-induced EGFR phosphorylation in a concentration-dependent fashion. In H1975 cells, which harbor constitutively active double-mutant EGFR, basal p-EGFR expression was high without stimulating with EGF (Figure 8B). Treatment with 5 μM osimertinib significantly suppressed (*p* < 0.0001) p-EGFR levels compared to the control. Similarly, asiatic acid significantly reduced p-EGFR levels in H1975 cells in a dose-dependent manner.

Furthermore, the cellular thermal shift assay (CETSA) revealed that treatment with 100 μM asiatic acid markedly enhanced the stability of the EGFR protein in A549 cells, indicating strong binding affinity toward EGFR (Figure S9). Collectively, these findings indicated that asiatic acid effectively suppresses EGFR activation in both A549 and H1975 NSCLC cells, highlighting its potential as a novel TKI targeting both wild-type and mutant forms of EGFR.

## 4. Discussion

Currently, chemotherapy is widely used as a treatment for patients with wild-type EGFR, while TKIs are employed for those with EGFR mutations [36]. However, a major issue of chemotherapy is its lack of selectivity for cancer cells, leading to significant toxicity in normal cells [37]. Furthermore, acquired resistance to TKIs caused by the T790M mutation in the EGFR poses a significant therapeutic challenge [11]. Together, these issues—non-selective cytotoxicity and drug resistance—highlight the urgent need for novel anticancer agents capable of effectively targeting both wild-type and T790M-mutat EGFR.

Previous studies have shown that asiatic acid effectively inhibited the proliferation of various cancer cells, including human colon cancer cells (SW480 and HCT116) [20], human liver cancer cells (HepG2) [22], and human breast cancer cells (MCF-7 and MDA-MB-231) [21]. Additionally, asiatic acid and its derivatives have been reported to exhibit antiproliferative effect on NSCLC cells [38,39]. Consistent with these findings, our results showed that asiatic acid isolated from *C. asiatica* effectively inhibited the proliferation of A549 and H1975 NSCLC cells within a concentration range of 12.5–400 μM, while showing no cytotoxicity toward BEAS-2B normal lung cells at concentrations of 12.5–100 μM (Figure 4 and Table 2). Notably, H1975 cells, which harbor EGFR double mutations, were more sensitive to asiatic acid (IC_50_ = 36.55 ± 0.86 µM) compared with A549 cells expressing wild-type EGFR (IC_50_ = 64.52 ± 2.49 µM).

As shown in Figure 5, asiatic acid significantly induced apoptosis in both A549 and H1975 cells. This finding is supported by previous work from Wu et al., demonstrating that asiatic acid significantly induced apoptotic cell death in lung cancer cells both in vitro and in vivo [40]. PARP is a DNA repair protein that detects DNA damage and facilitates repair processes. During apoptosis, caspases cleave PARP into smaller fragments, and the presence of cleaved PARP serves as a molecular marker of apoptosis, reflecting the loss of PARP’s DNA repair function and the progression of programmed cell death [41,42]. Consistent with this, our results showed that asiatic acid significantly increased cleaved PARP levels in both A549 and H1975 cells (Figure 6).

Cancer progression and growth are closely associated with the activation of intracellular signaling pathways mediated by EGFR, which is overexpressed in up to 60% of NSCLC cases [43,44]. As shown in Figure 8, treatment with asiatic acid significantly suppressed EGFR activation (p-EGFR) in A549 and H1975 NSCLC cell lines. Supportively, molecular docking demonstrated that asiatic acid could bind to the ATP-binding pocket of wild-type and double-mutant EGFR with binding energies superior to those of erlotinib and osimertinib, and interacted with key active-site residues (Figure 3), leading to suppression of EGFR tyrosine kinase activity (Figure 2 and Table 1).

EGFR phosphorylation activates multiple downstream signaling pathways, including MAPK/ERK and PI3K/Akt cascades [45,46]. ERK activation, a common mechanism in many cell types, is often associated with cancer progression [47]. Overactivation of ERK and Akt, frequently triggered by p-EGFR, contributes to aggressive tumor phenotypes and therapy resistance [48]. As shown in Figure 7, A549 and H1975 cells exhibited high levels of p-ERK and p-Akt, which were significantly suppressed by treatment with asiatic acid or the known TKIs erlotinib and osimertinib. These findings align with previous reports demonstrating that asiatic acid and its analog significantly downregulated p-ERK [38] and p-Akt [49] in A549 cells.

## 5. Conclusions

Our findings highlight the promising anticancer potential of asiatic acid from *C. asiatica*, which demonstrated potent EGFR inhibitory activity, selective cytotoxicity toward NSCLC cells, apoptosis induction, and effective suppression of EGFR-mediated signaling pathways. By targeting both wild-type and mutant forms of EGFR, asiatic acid emerges as a candidate for further development as a targeted therapeutic agent for lung cancer.

## Figures and Tables

**Table 1:** The IC_50_ values for kinase inhibition against the wild-type and double mutant (T790M/L858R) forms of EGFR.

Compounds	IC_50_ (nM) ^a^	
	Wild-Type EGFR	L858R/T790M EGFR
Asiaticoside	>80	>80
Asiatic acid	0.035 ± 0.002	0.348 ± 0.055
Erlotinib	0.062 ± 0.009	-
Osimertinib	-	0.116 ± 0.038

^a^ Data are presented as mean ± SEM (*n* = 3).

**Table 2:** The IC_50_ values of asiatic acid against A549 lung cancer cells, H1975 lung cancer cells, and BEAS-2B normal lung epithelial cells.

Cell Lines	IC_50_ (μM) ^a^
A549	64.52 ± 2.49
H1975	36.55 ± 0.86
BEAS-2B	184.10 ± 5.38

^a^ Data are shown as mean ± SEM (*n* = 3).

## Data Availability

All the applicable data have been provided in the manuscript. The authors will provide additional details if necessary.

## Supplementary Materials

The following supporting information can be downloaded at: https://www.mdpi.com/article/10.3390/biom15101410/s1, File S1: Figure S1: ^1^H NMR spectrum of asiaticoside (400 MHz, DMSO-*d*_6_); Figure S2: ^13^C NMR spectrum of asiaticoside (100 MHz, DMSO-*d*_6_); Figure S3: HR-TOFMS spectrum of asiaticoside; Figure S4: ^1^H NMR spectrum of asiatic acid (400 MHz, DMSO-*d*_6_); Figure S5: ^13^C NMR spectrum of asiatic acid (100 MHz, DMSO-*d*_6_); Figure S6: HR-TOFMS spectrum of asiatic acid; Figure S7: Re-docking of the co-crystallized ligand to (A) wild-type EGFR and (B) double mutant EGFR; Figure S8: EGFR and FGFR kinase inhibitory activities of asiatic acid; Figure S9: The cellular thermal shift assay; Table S1: Effect of osimertinib on H1975 cell viability; Table S2: Primary antibodies used for Western blot analysis. File S2: Original WB images.

## References

[1] Din S.R.U., Saeed S., Khan S.U., Arbi F.M., Xuefang G., Zhong M. (2023). Bacteria-driven cancer therapy: Exploring advancements and challenges. Crit. Rev. Oncol. Hematol..

[2] Anand U., Dey A., Chandel A.K.S., Sanyal R., Mishra A., Pandey D.K., De Falco V., Upadhyay A., Kandimalla R., Chaudhary A. (2023). Cancer chemotherapy and beyond: Current status, drug candidates, associated risks and progress in targeted therapeutics. Genes. Dis..

[3] Wang Z., Liu S., Zhang M., Liu M. (2025). Dual roles of methylglyoxal in cancer. Front. Oncol..

[4] Yu X., Wang Z., Chen Y., Yin G., Liu J., Chen W., Zhu L., Xu W., Li X. (2021). The predictive role of immune related subgroup classification in immune checkpoint blockade therapy for lung adenocarcinoma. Front. Genet..

[5] Li L., Chen C., Xiang Q., Fan S., Xiao T., Chen Y., Zheng D. (2022). Transient receptor potential cation channel subfamily V member 1 expression promotes chemoresistance in non-small-cell lung cancer. Front. Oncol..

[6] Bai Y., Yang W., Käsmann L., Sorich M.J., Tao H., Hu Y. (2024). Immunotherapy for advanced non-small cell lung cancer with negative programmed death-ligand 1 expression: A literature review. Transl. Lung Cancer Res..

[7] Gutierrez E., Cahatol I., Bailey C.A.R., Lafargue A., Zhang N., Song Y., Tian H., Zhang Y., Chan R., Gu K. (2019). Regulation of RhoB gene expression during tumorigenesis and aging process and its potential applications in these processes. Cancers.

[8] Haraldsdottir S., Bekaii-Saab T. (2013). Integrating anti-EGFR therapies in metastatic colorectal cancer. J. Gastrointest. Oncol..

[9] Wiest N., Majeed U., Seegobin K., Zhao Y., Lou Y., Manochakian R. (2021). Role of immune checkpoint inhibitor therapy in advanced EGFR-mutant non-small cell lung cancer. Front. Oncol..

[10] Šutić M., Vukić A., Baranašić J., Försti A., Džubur F., Samaržija M., Jakopović M., Brčić L., Knežević J. (2021). Diagnostic, predictive, and prognostic biomarkers in non-small cell lung cancer (NSCLC) management. J. Pers. Med..

[11] Imamura F., Uchida J., Kukita Y., Kumagai T., Nishino K., Inoue T., Kimura M., Oba S., Kato K. (2016). Monitoring of treatment responses and clonal evolution of tumor cells by circulating tumor DNA of heterogeneous mutant *EGFR* genes in lung cancer. Lung Cancer.

[12] Wiciński M., Fajkiel-Madajczyk A., Kurant Z., Gajewska S., Kurant D., Kurant M., Sousak M. (2024). Can asiatic acid from *Centella asiatica* be a potential remedy in cancer therapy?—A review. Cancers.

[13] Gohil K.J., Patel J.A., Gajjar A.K. (2010). Pharmacological review on *Centella asiatica*: A potential herbal cure-all. Indian J. Pharm. Sci..

[14] Arribas-López E., Zand N., Ojo O., Snowden M.J., Kochhar T. (2022). A systematic review of the effect of *Centella asiatica* on wound healing. Int. J. Environ. Res. Public Health.

[15] Rashid M.H.-O., Akter M.M., Uddin J., Islam S., Rahman M., Jahan K., Sarker M.M.R., Sadik G. (2023). Antioxidant, cytotoxic, antibacterial and thrombolytic activities of *Centella asiatica* L.: Possible role of phenolics and flavonoids. Clin. Phytoscience.

[16] Shin H.Y., Kim Y.S., Ha E.J., Koo J.P., Jeong W.B., Joung M.Y., Shin K.-S., Yu K.-W. (2024). Anti-inflammatory action and associated intracellular signaling of *Centella asiatica* extract on lipopolysaccharide-stimulated RAW 264.7 macrophage. Food Biosci..

[17] Ferah Okkay I., Okkay U., Aydin I.C., Bayram C., Ertugrul M.S., Mendil A.S., Hacimuftuoglu A. (2022). *Centella asiatica* extract protects against cisplatin-induced hepatotoxicity via targeting oxidative stress, inflammation, and apoptosis. Environ. Sci. Pollut. Res. Int..

[18] Singh L.S., Singh W.S. (2024). *Centella asiatica* and its bioactive compounds: A comprehensive approach to managing hyperglycemia and associated disorders. Discov. Plants.

[19] Al-Saeedi F.J. (2014). Study of the cytotoxicity of asiaticoside on rats and tumour cells. BMC Cancer.

[20] Hao Y., Huang J., Ma Y., Chen W., Fan Q., Sun X., Shao M., Cai H. (2018). Asiatic acid inhibits proliferation, migration and induces apoptosis by regulating Pdcd4 via the PI3K/Akt/mTOR/p70S6K signaling pathway in human colon carcinoma cells. Oncol. Lett..

[21] Hsu Y.-L., Kuo P.-L., Lin L.-T., Lin C.-C. (2005). Asiatic acid, a triterpene, induces apoptosis and cell cycle arrest through activation of extracellular signal-regulated kinase and p38 mitogen-activated protein kinase pathways in human breast cancer cells. J. Pharmacol. Exp. Ther..

[22] Lee Y.S., Jin D.-Q., Kwon E.J., Park S.H., Lee E.-S., Jeong T.C., Nam D.H., Huh K., Kim J.-A. (2002). Asiatic acid, a triterpene, induces apoptosis through intracellular Ca^2+^ release and enhanced expression of p53 in HepG2 human hepatoma cells. Cancer Lett..

[23] Li Z., You K., Li J., Wang Y., Xu H., Gao B., Wang J. (2016). Madecassoside suppresses proliferation and invasiveness of HGF-induced human hepatocellular carcinoma cells via PKC-cMET-ERK1/2-COX-2-PGE2 pathway. Int. Immunopharmacol..

[24] Valdeira A.S.C., Darvishi E., Woldemichael G.M., Beutler J.A., Gustafson K.R., Salvador J.A.R. (2019). Madecassic acid derivatives as potential anticancer agents: Synthesis and cytotoxic evaluation. J. Nat. Prod..

[25] Gao H., Wang Z. (2006). Triterpenoid saponins and phenylethanoid glycosides from stem of Akebia trifoliata var. australis. Phytochemistry.

[26] Acebey-Castellon I.L., Voutquenne-Nazabadioko L., Doan Thi Mai H., Roseau N., Bouthagane N., Muhammad D., Le Magrex Debar E., Gangloff S.C., Litaudon M., Sevenet T. (2011). Triterpenoid saponins from *Symplocos lancifolia*. J. Nat. Prod..

[27] Tolls J., van Dijk J., Verbruggen E.J.M., Hermens J.L.M., Loeprecht B., Schüürmann G. (2002). Aqueous solubility−molecular size relationships:  A mechanistic case study using C10- to C19-alkanes. J. Phys. Chem..

[28] Aiebchun T., Mahalapbutr P., Auepattanapong A., Khaikate O., Seetaha S., Tabtimmai L., Kuhakarn C., Choowongkomon K., Rungrotmongkol T. (2021). Identification of vinyl sulfone derivatives as EGFR tyrosine kinase inhibitor: In vitro and in silico studies. Molecules.

[29] Lin G.B., Chen W.T., Kuo Y.Y., Liu H.H., Chen Y.M., Leu S.J., Chao C.Y. (2025). Thermal cycling—hyperthermia sensitizes non—small cell lung cancer A549 cells to EGFR tyrosine kinase inhibitor erlotinib. Oncol. Rep..

[30] Alhazzani K., Alsahli M., Alanazi A.Z., Algahtani M., Alenezi A.A., Alhoshani A., Alqinyah M., Alhamed A.S., Alhosaini K. (2023). Augmented antitumor effects of erlotinib and cabozantinib on A549 non-small cell lung cancer: In vitro and in vivo studies. Saudi Pharm. J..

[31] Jaramillo M.L., Banville M., Collins C., Paul-Roc B., Bourget L., O’Connor-McCourt M. (2008). Differential sensitivity of A549 non-small lung carcinoma cell responses to epidermal growth factor receptor pathway inhibitors. Cancer Biol. Ther..

[32] Tomshine J.C., Severson S.R., Wigle D.A., Sun Z., Beleford D.A., Shridhar V., Horazdovsky B.F. (2009). Cell proliferation and epidermal growth factor signaling in non-small cell lung adenocarcinoma cell lines are dependent on Rin1. J. Biol. Chem..

[33] Reguart N., Cardona A.F., Rosell R. (2010). Role of erlotinib in first-line and maintenance treatment of advanced non-small-cell lung cancer. Cancer Manag. Res..

[34] Li Y., Mao T., Wang J., Zheng H., Hu Z., Cao P., Yang S., Zhu L., Guo S., Zhao X. (2023). Toward the next generation EGFR inhibitors: An overview of osimertinib resistance mediated by EGFR mutations in non-small cell lung cancer. Cell Commun. Signal..

[35] Onodera K., Sakurada A., Notsuda H., Watanabe T., Matsuda Y., Noda M., Endo C., Okada Y. (2018). Growth inhibition of KRAS—and EGFR—mutant lung adenocarcinoma by cosuppression of STAT3 and the SRC/ARHGAP35 axis. Oncol. Rep..

[36] Landi L., Cappuzzo F. (2011). Front-line therapy in lung cancer with mutations in EGFR. Nat. Rev. Clin. Oncol..

[37] Blagosklonny M.V. (2023). Selective protection of normal cells from chemotherapy, while killing drug-resistant cancer cells. Oncotarget.

[38] Wang L., Xu J., Zhao C., Zhao L., Feng B. (2013). Antiproliferative, cell-cycle dysregulation effects of novel asiatic acid derivatives on human non-small cell lung cancer cells. Chem. Pharm. Bull..

[39] Singh J., Hussain Y., Meena A., Sinha R.A., Luqman S. (2024). Asiatic acid impedes NSCLC progression by inhibiting COX-2 and modulating PI3K signaling. FEBS Lett..

[40] Wu T., Geng J., Guo W., Gao J., Zhu X. (2017). Asiatic acid inhibits lung cancer cell growth in vitro and in vivo by destroying mitochondria. Acta Pharm. Sin. B.

[41] Wang Y., Luo W., Wang Y. (2019). PARP-1 and its associated nucleases in DNA damage response. DNA Repair.

[42] Mashimo M., Onishi M., Uno A., Tanimichi A., Nobeyama A., Mori M., Yamada S., Negi S., Bu X., Kato J. (2021). The 89-kDa PARP1 cleavage fragment serves as a cytoplasmic PAR carrier to induce AIF-mediated apoptosis. J. Biol. Chem..

[43] Ciardiello F., Tortora G. (2002). Anti-epidermal growth factor receptor drugs in cancer therapy. Expert. Opin. Investig. Drugs.

[44] Hirsch F.R., Varella-Garcia M., Bunn P.A., Di Maria M.V., Veve R., Bremmes R.M., Barón A.E., Zeng C., Franklin W.A. (2003). Epidermal growth factor receptor in non-small-cell lung carcinomas: Correlation between gene copy number and protein expression and impact on prognosis. J. Clin. Oncol..

[45] Montor W.R., Salas A.R.O.S.E., de Melo F.H.M. (2018). Receptor tyrosine kinases and downstream pathways as druggable targets for cancer treatment: The current arsenal of inhibitors. Mol. Cancer.

[46] Tito C., Masciarelli S., Colotti G., Fazi F. (2025). EGF receptor in organ development, tissue homeostasis and regeneration. J. Biomed. Sci..

[47] Muta Y., Matsuda M., Imajo M. (2019). Divergent dynamics and functions of ERK MAP kinase signaling in development, homeostasis and cancer: Lessons from fluorescent bioimaging. Cancers.

[48] Martini M., Chiara D.S.M., Laura B., Federico G., Hirsch E. (2014). PI3K/AKT signaling pathway and cancer: An updated review. Ann. Med..

[49] Chen R., Zhang W., Zhang M., Liu W., Feng W., Zhang Y. (2025). Asiatic acid in anticancer effects: Emerging roles and mechanisms. Front. Pharmacol..

