# Supplementary material for: Asiatic Acid from Centella asiatica as a Potent EGFR Tyrosine Kinase Inhibitor with Anticancer Activity in NSCLC Cells Harboring Wild-Type and T790M-Mutated EGFR
Source: Biomolecules. 2025 Oct 3;15(10):1410. doi: 10.3390/biom15101410 (PMC12563520; doi:10.3390/biom15101410)
Supplement: Supplementary file 1 [file biomolecules-15-01410-s001.zip › biomolecules-3846435-supplementary.pdf]

Supplementary Materials

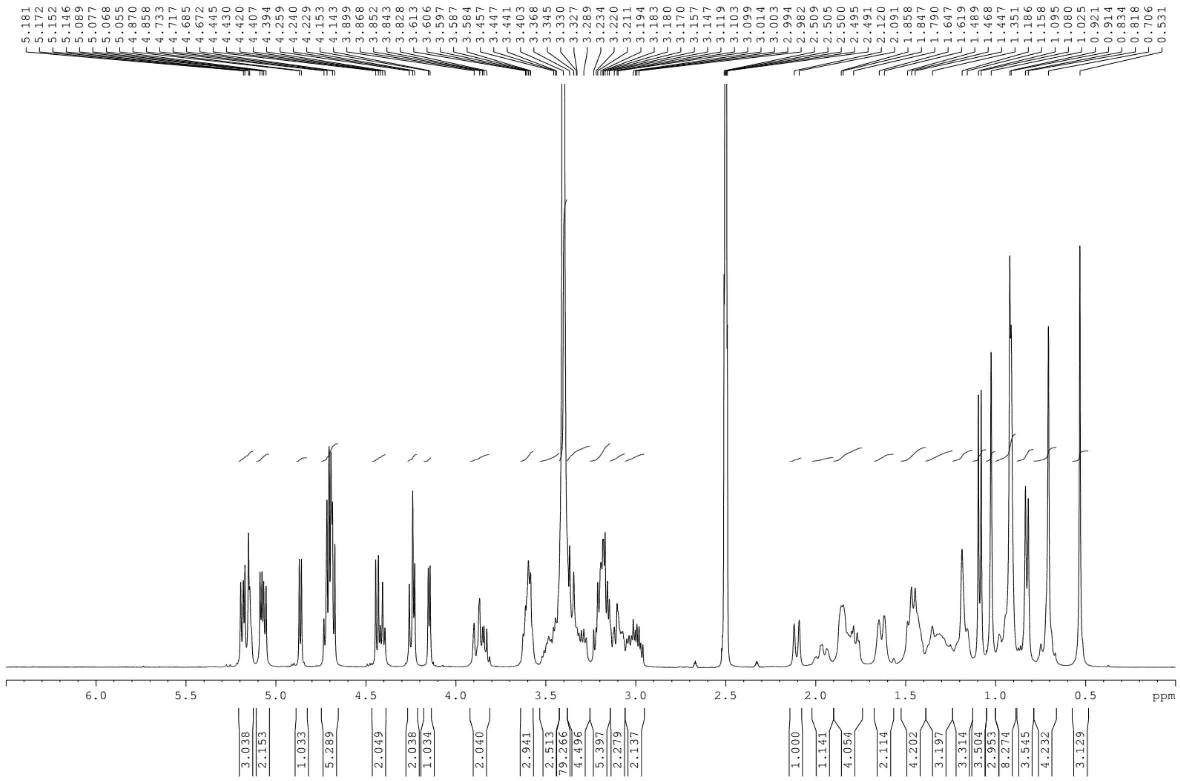

Figure S1. ^{1}H NMR spectrum of asiaticoside (400 MHz, DMSO-d_6).

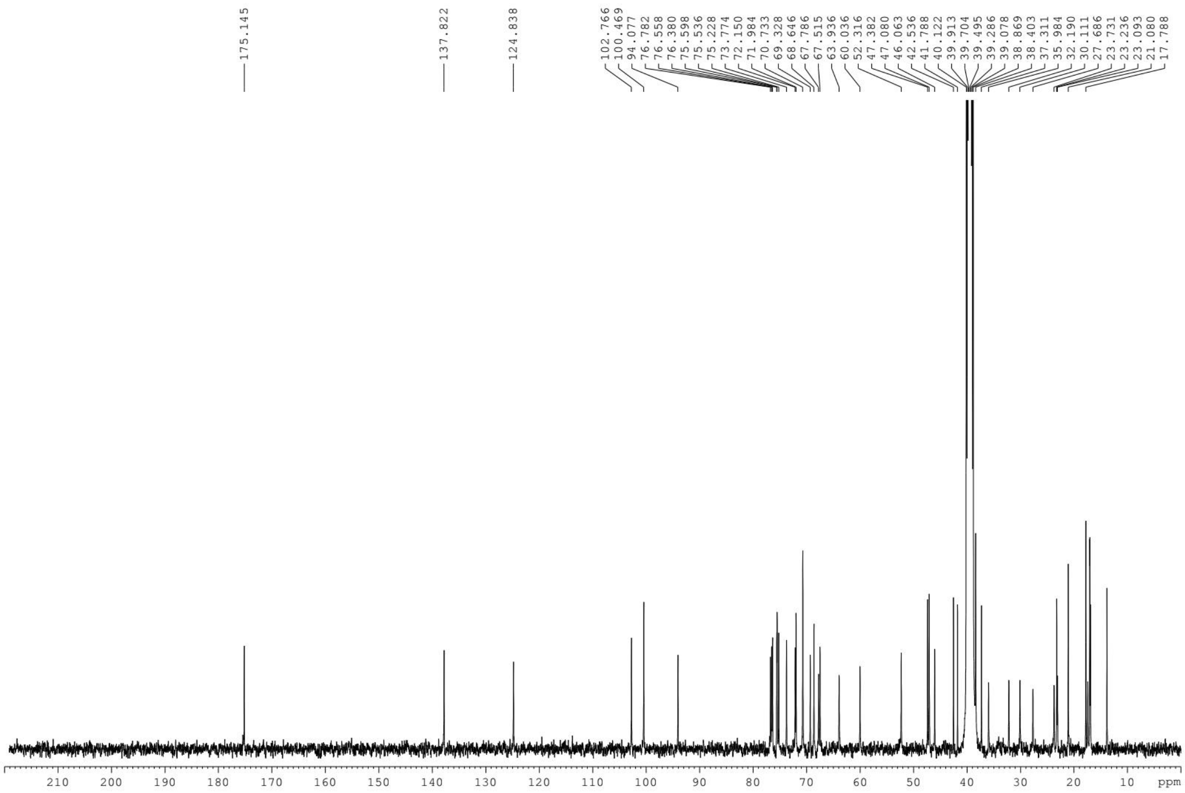

Figure S2. ^{13}C NMR spectrum of asiaticoside (100 MHz, DMSO-d_6).

Acquisition Parameter

Source Type	ESI	Ion Polarity	Positive	Set Nebulizer	2.0 Bar
Focus	Not active			Set Dry Heater	200 °C
Scan Begin	50 m/z	Set Capillary	4500 V	Set Dry Gas	8.0 l/min
Scan End	2000 m/z	Set End Plate Offset	-500 V	Set Divert Valve	Waste

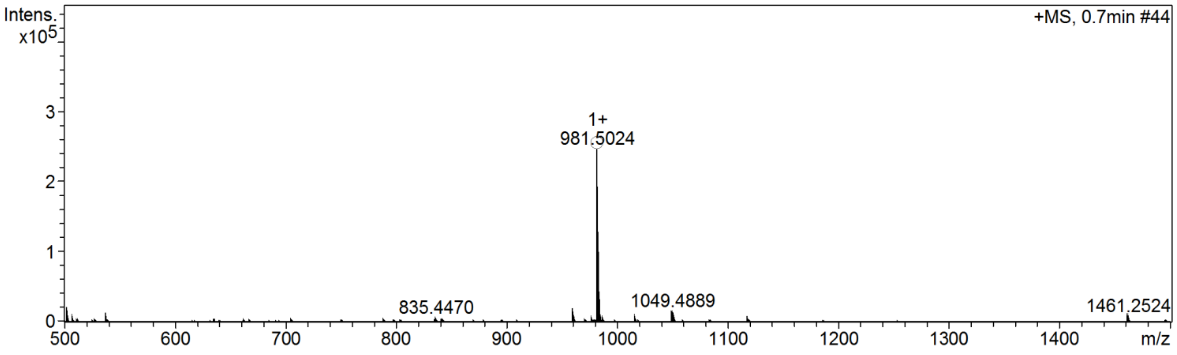

Meas. m/z	#	Ion Formula	m/z	|err| [mDa]	err [ppm]	Mean err [ppm]	rdb	N-Rule	e⁻ Conf	mSigma
981.502426	1	C48H78NaO19	981.502951	0.5	0.5	0.7	9.5	ok	even	5.3

Figure S3. HR-TOFMS spectrum of asiaticoside.

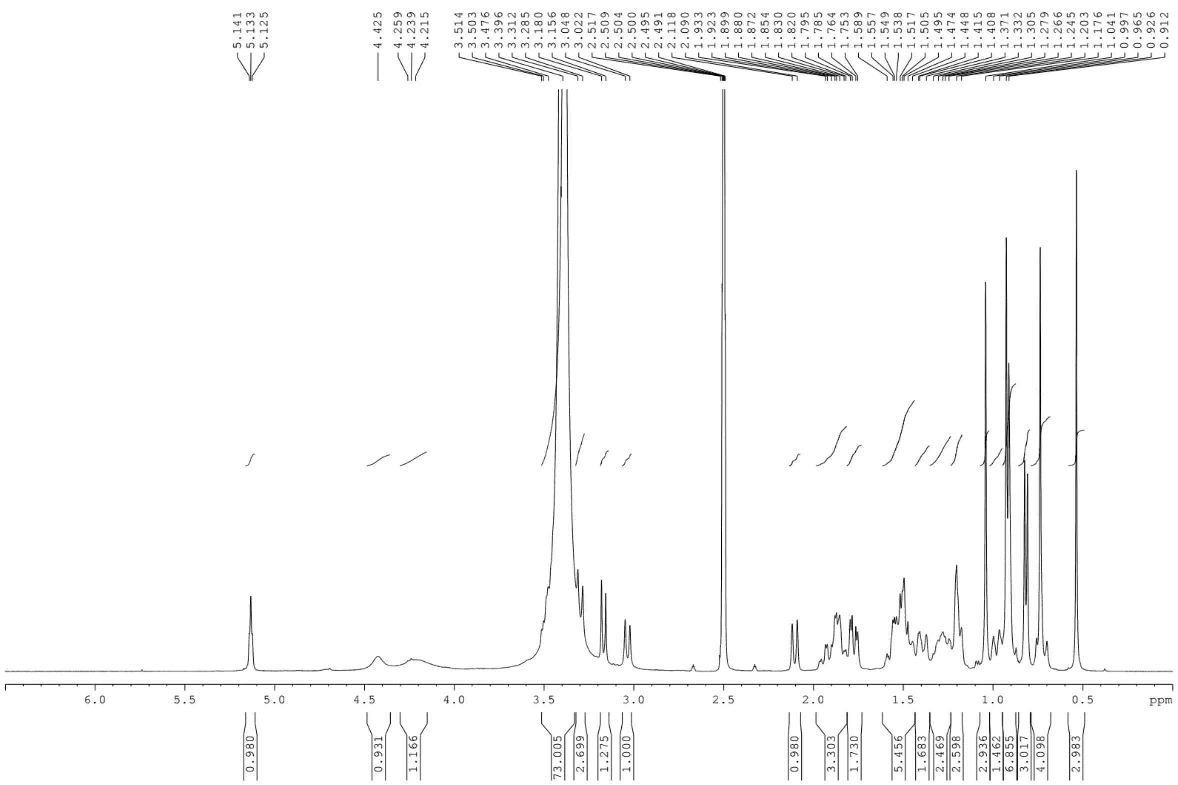

Figure S4. ^{1}H NMR spectrum of asiatic acid (400 MHz, DMSO-d_6).

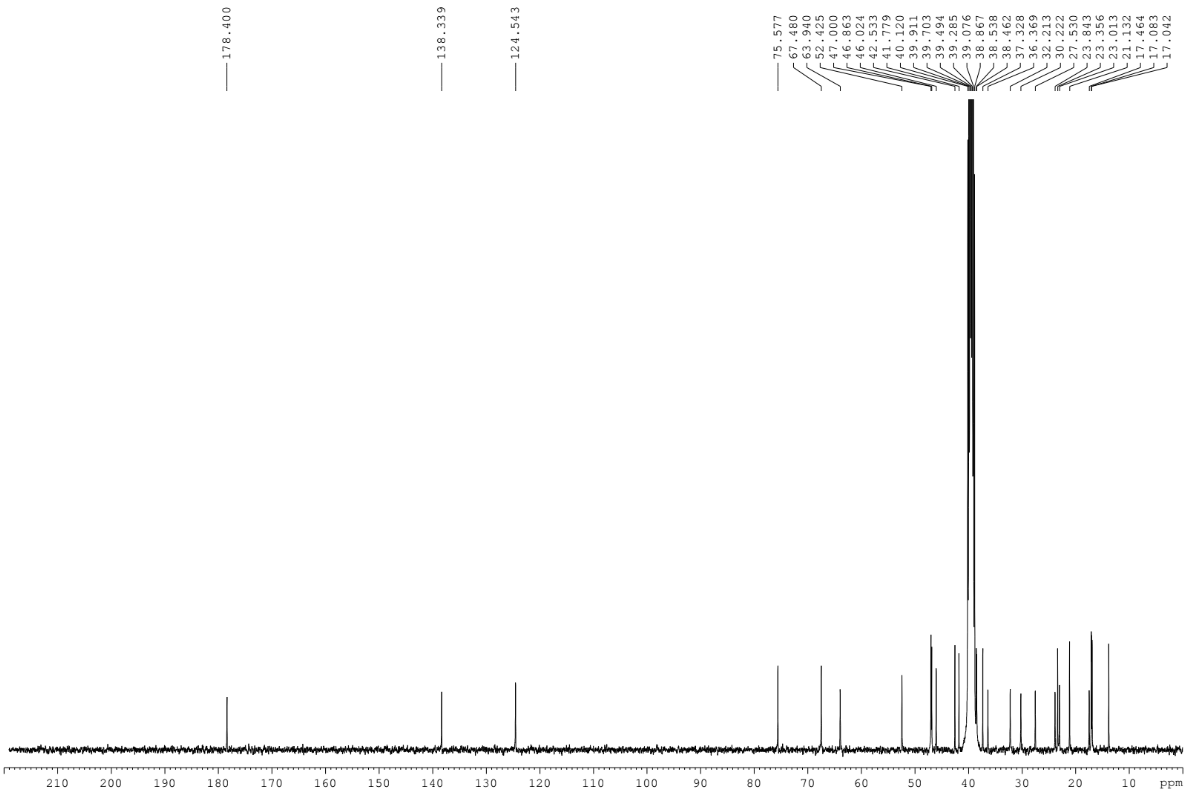

Figure S5. ^{13}C NMR spectrum of asiatic acid (100 MHz, DMSO-d_6).

Acquisition Parameter

Source Type	ESI	Ion Polarity	Positive	Set Nebulizer	2.0 Bar
Focus	Not active			Set Dry Heater	200 °C
Scan Begin	50 m/z	Set Capillary	4500 V	Set Dry Gas	8.0 l/min
Scan End	2000 m/z	Set End Plate Offset	-500 V	Set Divert Valve	Waste

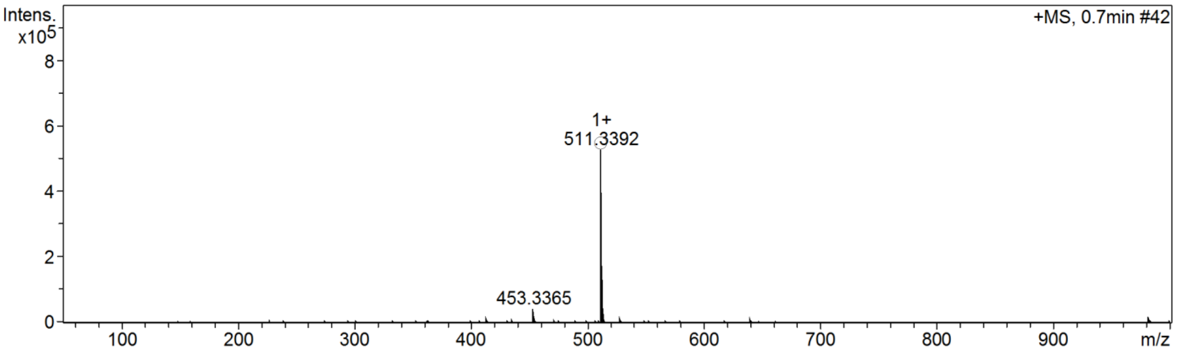

Meas. m/z	#	Ion Formula	m/z	|err| [mDa]	err [ppm]	Mean err [ppm]	rdb	N-Rule	e⁻ Conf	mSigma
511.339227	1	C30H48NaO5	511.339395	0.2	-0.3	0.5	6.5	ok	even	2.0

Figure S6. HR-TOFMS spectrum of asiatic acid.

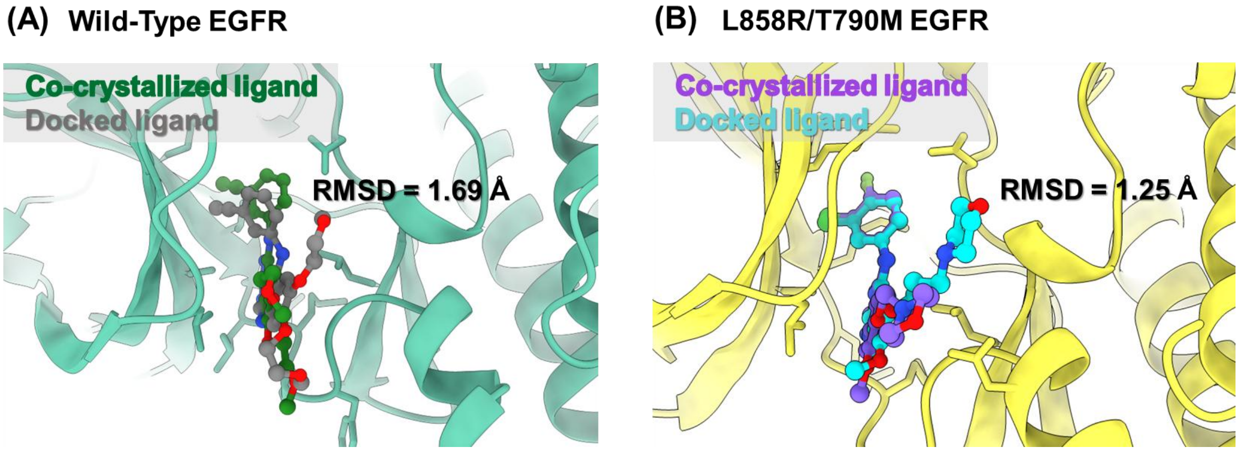

Figure S7. Re-docking of the co-crystallized ligand to (A) wild-type EGFR and (B) double mutant EGFR.

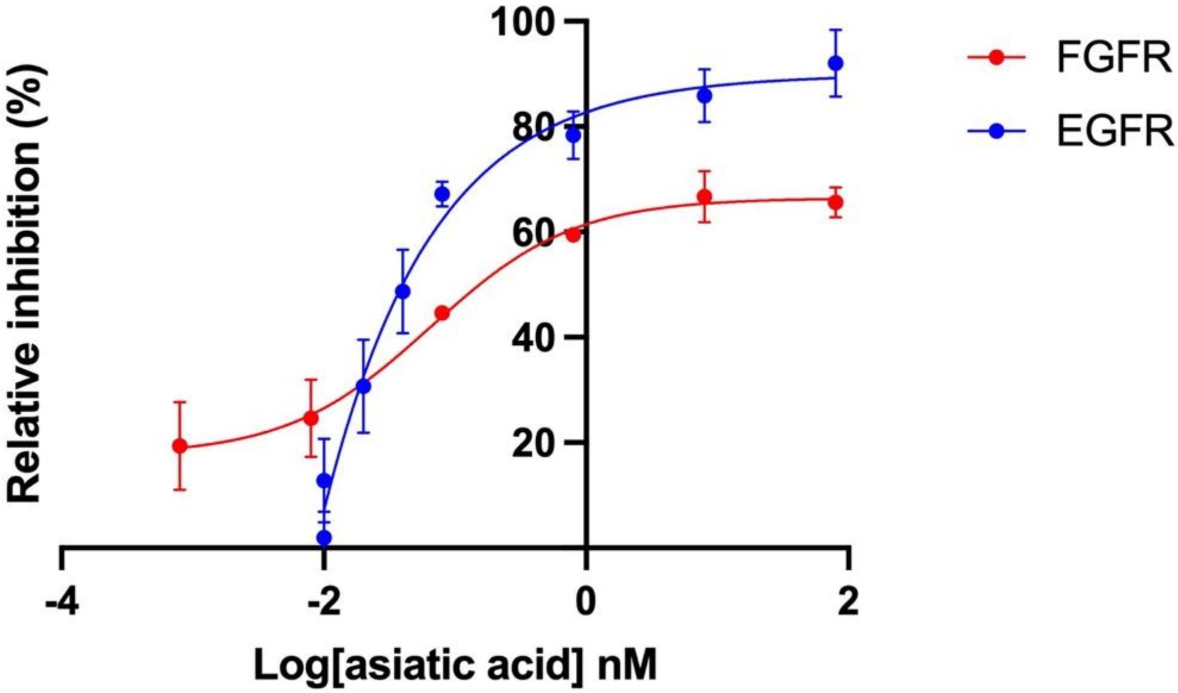

Figure S8. EGFR and FGFR kinase inhibitory activities of asiatic acid**.**

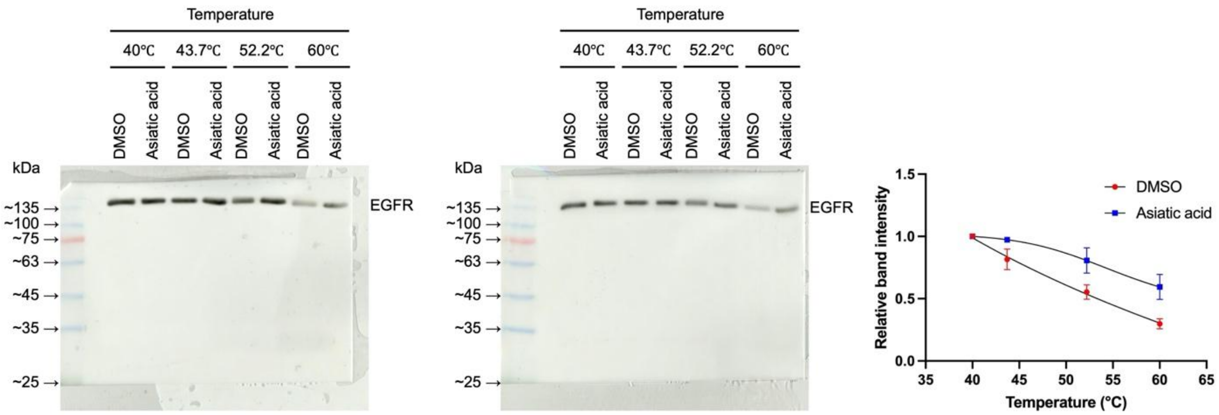

Figure S9. The cellular thermal shift assay. The stability of EGFR protein in A549 cells was assessed by treating cell lysates with either 0.2% DMSO or 100 μM asiatic acid for 1 hour. Following treatment, samples were heated at 40–60 °C for 3 minutes, rapidly cooled on ice for 10 minutes, and then centrifuged at 12,000 rpm at 4 °C for 30 minutes. The supernatants were analyzed by Western blot, and relative band intensities were plotted against the corresponding heating temperatures. Data are expressed as mean ± SEM (n = 2).

Table S1. Effect of osimertinib on H1975 cell viability (*n* = 3).

Treatment (Osimertinib)	Cell viability (%)					
	**#1**	**#2**	**#3**	**Mean**	**SD**	**SEM**
DMEM	100.00	100.00	100.00	100.00	0.00	0.00
0.8% DMSO	100.98	100.19	100.41	100.53	0.41	0.24
3.125 μM	61.47	58.99	64.15	61.54	2.58	1.49
6.25 μM	42.94	43.82	47.66	44.81	2.51	1.45
12.5 μM	22.09	23.60	25.66	23.78	1.80	1.04
25 μM	11.29	11.99	13.03	12.10	0.88	0.51
50 μM	7.24	7.87	8.55	7.89	0.66	0.38
100 μM	0.12	0.56	0.61	0.43	0.27	0.16
IC_{50} (μM)	3.89	3.98	4.54	4.14	0.35	0.20

Table S2. Primary antibodies used for Western blot analysis.

Antibody name	Company	Catalog No.	Secondary antibody
p-ERK (Thr202/Tyr204)	Cell signaling Technology	#9101	goat anti-Rabbit IgG
ERK	Cell signaling Technology	#9102	goat anti-Rabbit IgG
p-Akt (Ser473)	Cell signaling Technology	#9271	goat anti-Rabbit IgG
Akt	Cell signaling Technology	#9272	goat anti-Rabbit IgG
p-EGFR (Tyr1068)	Cell signaling Technology	#2234	goat anti-Rabbit IgG
EGFR	Cell signaling Technology	#4267	goat anti-Rabbit IgG
Cleaved PARP (Asp214)	Cell signaling Technology	#5625	goat anti-Rabbit IgG
PARP	Cell signaling Technology	#9542	goat anti-Rabbit IgG
GAPDH	Sigma-Aldrich	#MAB374	goat anti-Mouse IgG
