# Supplementary material for: Asiatic Acid from Centella asiatica as a Potent EGFR Tyrosine Kinase Inhibitor with Anticancer Activity in NSCLC Cells Harboring Wild-Type and T790M-Mutated EGFR
Source: Biomolecules. 2025 Oct 3;15(10):1410. doi: 10.3390/biom15101410 (PMC12563520; doi:10.3390/biom15101410)
Supplement: Supplementary file 1 [file biomolecules-15-01410-s001.zip › Original pictures for western blot analysis.pdf]

Original pictures for western blot analysis - Figure 6A

PARP, Cleaved PARP, and GAPDH

Batch 1 **Batch 2** **Batch 3**

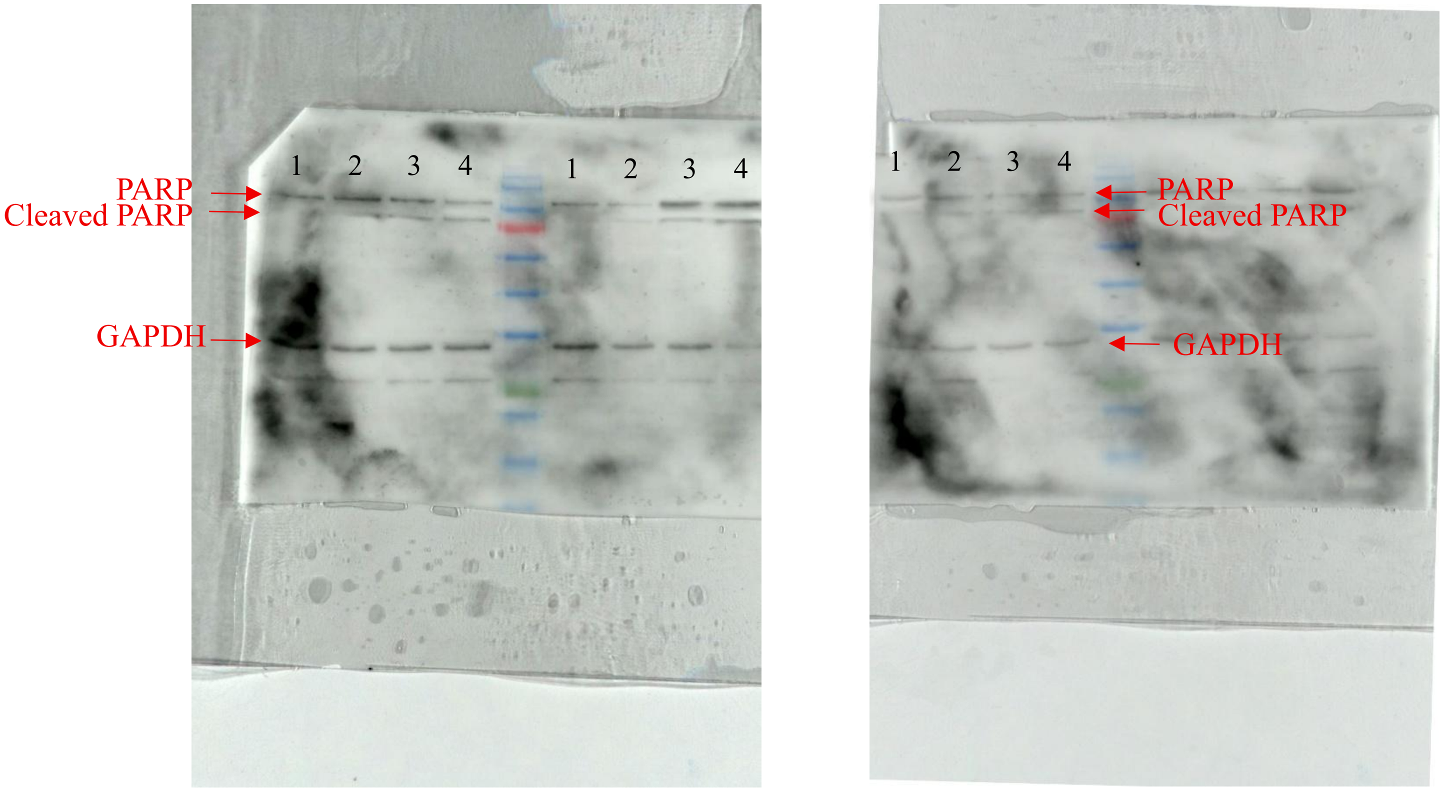

Batch 1

List	AS-PN005 Concentration	Cleaved PARP	PARP	GAPDH	Cleaved PARP expression	
					Cleaved PARP / PARP / GAPDH	Fold expression
1	0 µM	15308	119792	362471	3.52547E-07	1.00
2	25 µM	36041	127977	278861	1.0099E-06	2.86
3	50 µM	49363	114446	299434	1.44046E-06	4.09
4	100 µM	65245	79813	288758	2.831E-06	8.03

Batch 2

List	AS-PN005 Concentration	Cleaved PARP	PARP	GAPDH	Cleaved PARP expression	
					Cleaved PARP / PARP / GAPDH	Fold expression
1	0 µM	11613	66834	398838	4.35663E-07	1.00
2	25 µM	31172	62814	347305	1.42888E-06	3.28
3	50 µM	69297	91114	350823	2.16791E-06	4.98
4	100 µM	72252	90466	250694	3.18581E-06	7.31

Batch 3

List	AS-PN005 Concentration	Cleaved PARP	PARP	GAPDH	Cleaved PARP expression	
					Cleaved PARP / PARP / GAPDH	Fold expression
1	0 µM	87311	250411	849017	4.10676E-07	1.00
2	25 µM	257178	385221	507902	1.31445E-06	3.20
3	50 µM	214558	257233	485114	1.71939E-06	4.19
4	100 µM	365295	303413	558648	2.15512E-06	5.25

Average of cleaved PARP expression (fold)

List	AS-PN005 Concentration	Batch 1	Batch 2	Batch 3	Mean	SD	SEM
1	0 μM	1.00	1.00	1.00	1.00	0.00	0.00
2	25 μM	2.86	3.28	3.20	3.12	0.22	0.13
3	50 μM	4.09	4.98	4.19	4.42	0.49	0.28
4	100 μM	8.03	7.31	5.25	6.86	1.44	0.83

Original pictures for western blot analysis - Figure 6B

PARP and Cleaved PARP

Batch 1

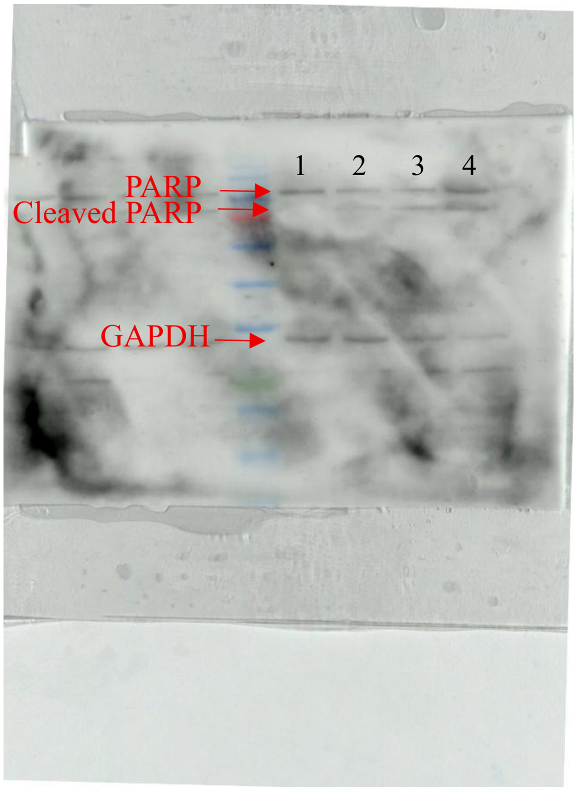

Batch 2 Batch 3

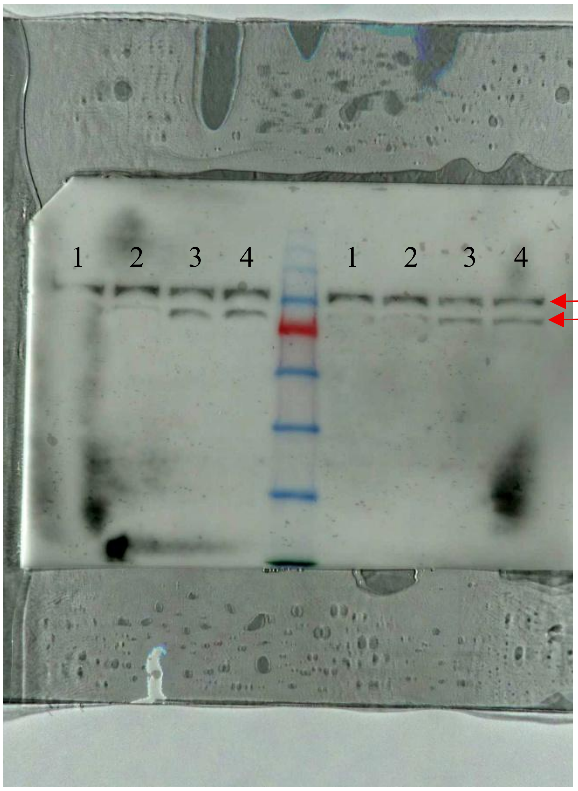

GAPDH

Batch 1

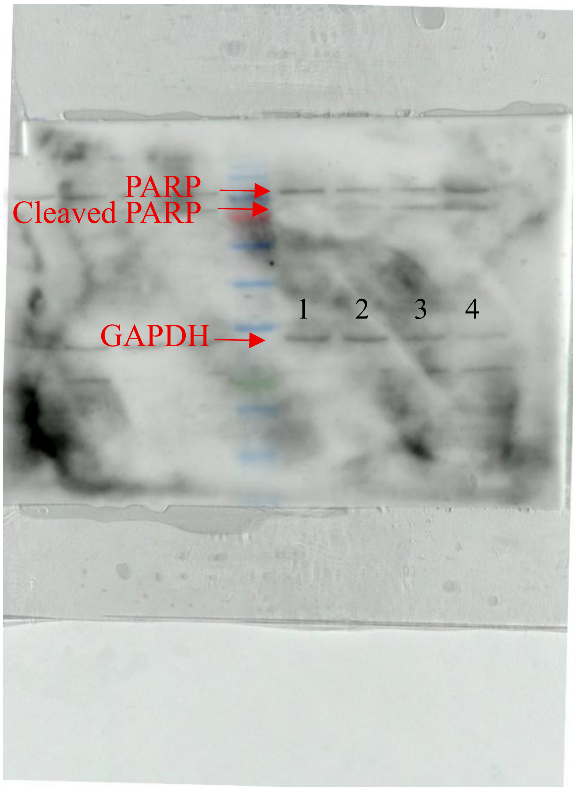

Batch 2 Batch 3

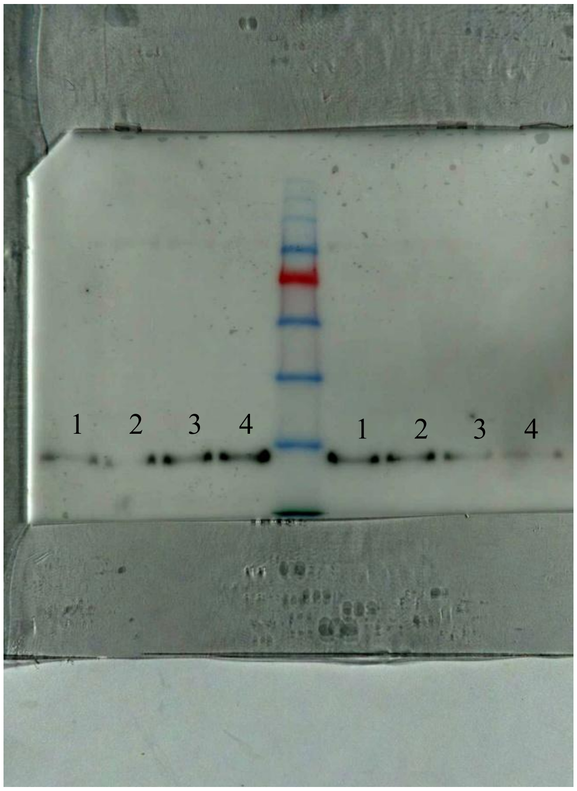

Batch 1

List	AS-PN005 Concentration	Cleaved PARP	PARP	GAPDH	Cleaved PARP expression	
					Cleaved PARP / PARP / GAPDH	Fold expression
1	0 μM	133821	537699	662856	3.75462E-07	1.00
2	12.5 μM	488732	299179	737016	2.21647E-06	5.90
3	25 μM	741163	333958	662540	3.34973E-06	8.92
4	50 μM	991855	331657	634954	4.70995E-06	12.54

Batch 2

List	AS-PN005 Concentration	Cleaved PARP	PARP	GAPDH	Cleaved PARP expression	
					Cleaved PARP / PARP / GAPDH	Fold expression
1	0 μM	8256	225784	113259	3.22852E-07	1.00
2	12.5 μM	127456	331548	135478	2.83756E-06	8.79
3	25 μM	244583	274581	257412	3.46041E-06	10.72
4	50 μM	331729	295143	296143	3.79533E-06	11.76

Batch 3

List	AS-PN005 Concentration	Cleaved PARP	PARP	GAPDH	Cleaved PARP expression	
					Cleaved PARP / PARP / GAPDH	Fold expression
1	0 μM	4078	624578	421563	1.54881E-08	1.00
2	12.5 μM	22495	568412	361245	1.09552E-07	7.07
3	25 μM	26216	488725	334894	1.60175E-07	10.34
4	50 μM	40578	461254	313158	2.80923E-07	18.14

Average of cleaved PARP expression (fold)

List	AS-PN005 Concentration	Batch 1	Batch 2	Batch 3	Mean	SD	SEM
1	0 μM	1.00	1.00	1.00	1.00	0.00	0.00
2	12.5 μM	5.90	8.79	7.07	7.26	1.45	0.84
3	25 μM	8.92	10.72	10.34	9.99	0.95	0.55
4	50 μM	12.54	11.76	18.14	14.15	3.48	2.01

Original pictures for western blot analysis - Figure 7A

p-ERK

Batch 1

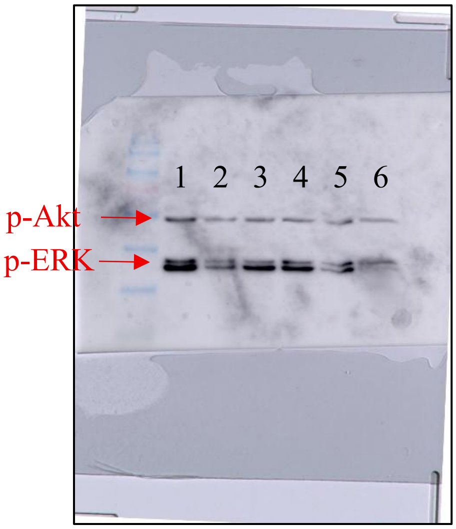

Batch 2

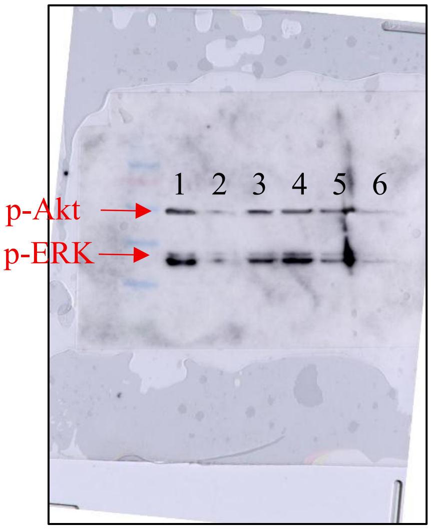

Batch 3

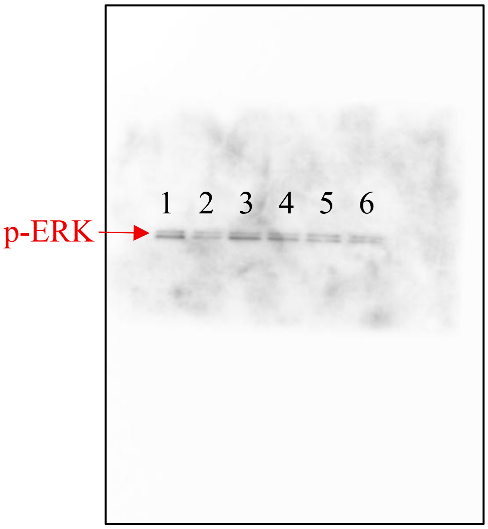

ERK

Batch 1

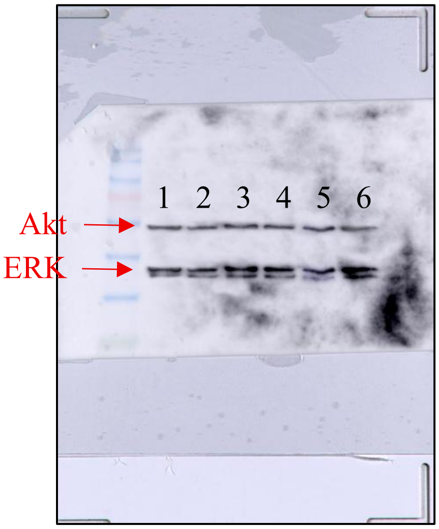

Batch 2

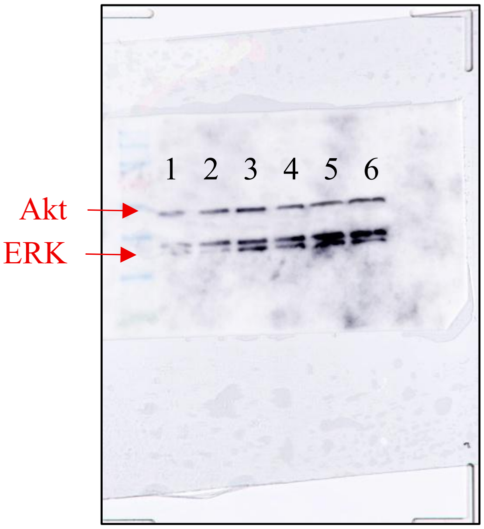

Batch 3

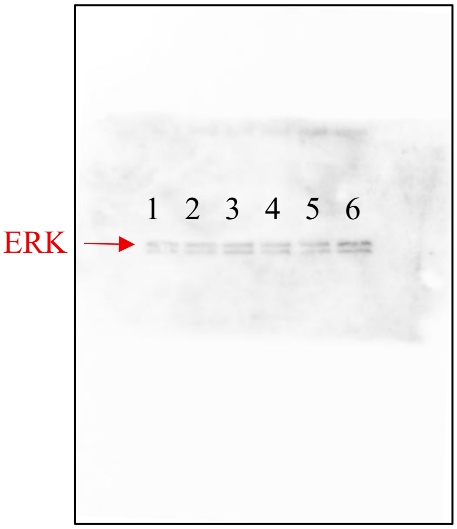

p-Akt

Batch 1 (same pic. as p-ERK)

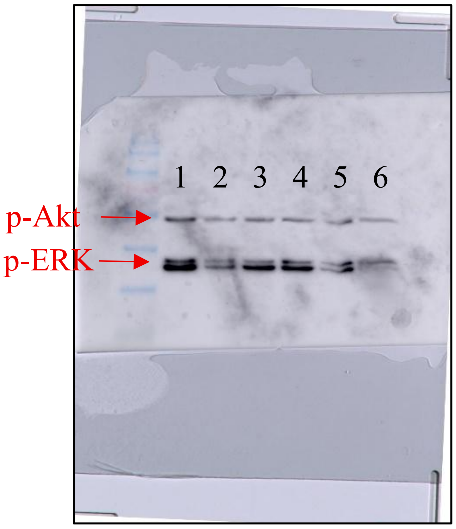

Batch 2 (same pic. as p-ERK)

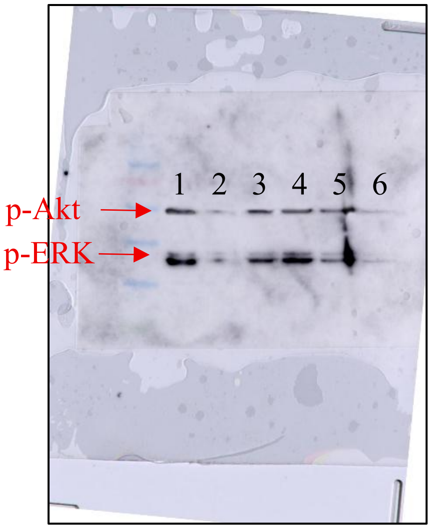

Batch 3

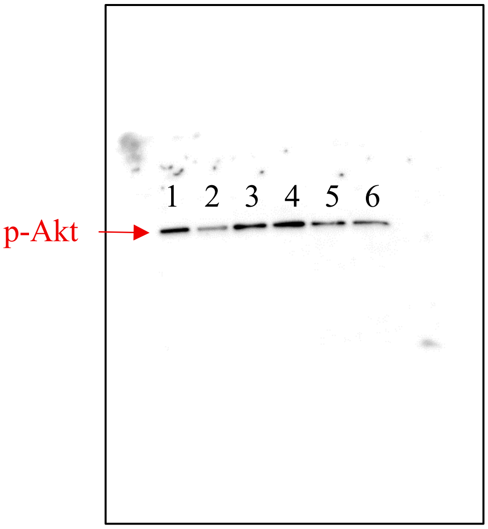

Akt

Batch 1 (same pic. as ERK)

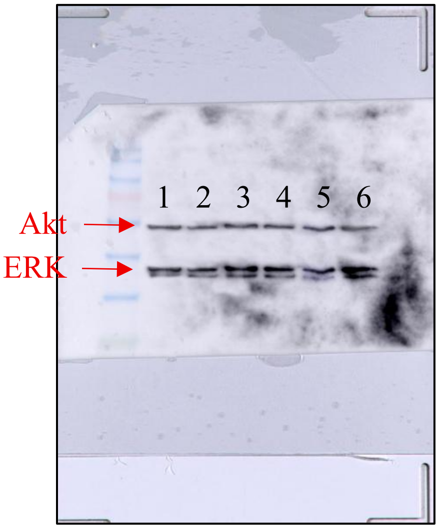

Batch 2 (same pic. as ERK)

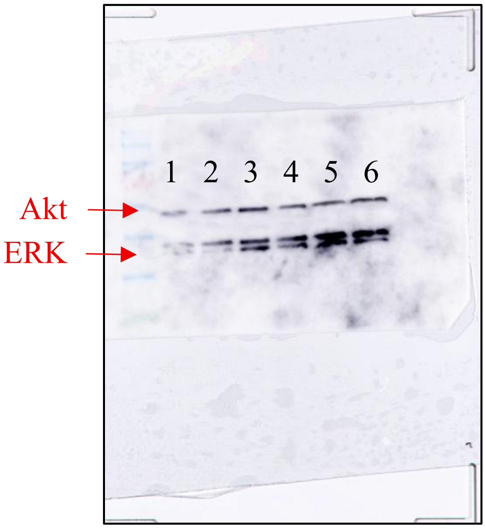

Batch 3

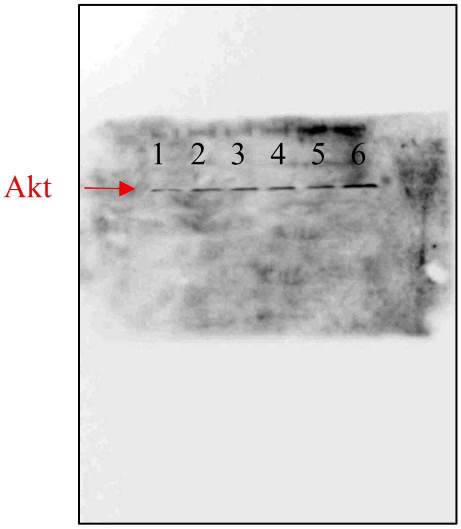

GAPDH

Batch 1

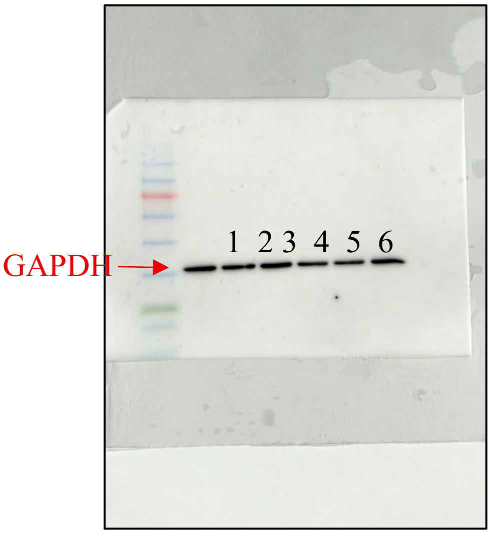

Batch 2

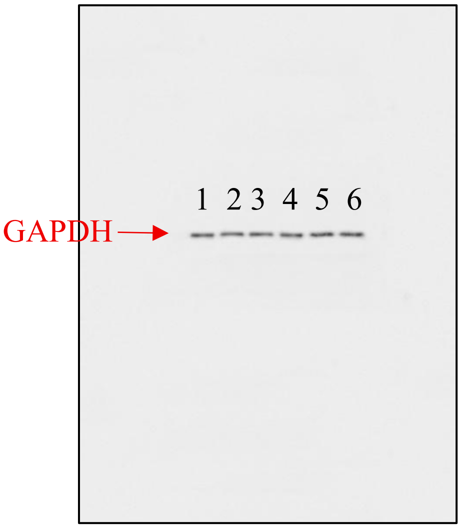

Batch 3

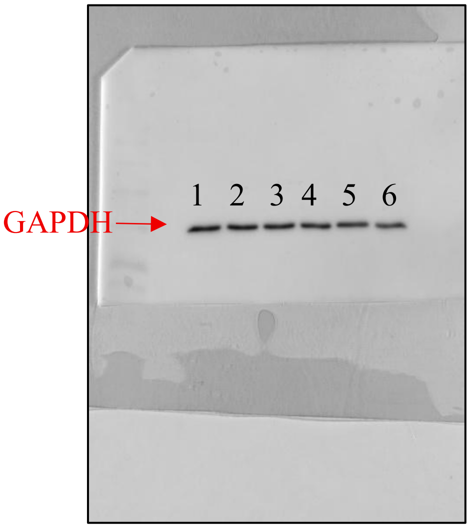

Batch 1

List	Treatment	p-ERK	ERK	GAPDH	p-ERK expression	
					p-ERK / ERK / GAPDH	Fold expression
1	DMSO	73808	292123	81682	0.000003093	1.00
2	Erlotinib	21067	261060	86757	0.000000930	0.30
3	AS-PN005_12.5 µM	59689	435887	105440	0.000001299	0.42
4	AS-PN005_25 µM	51085	453112	92456	0.000001219	0.39
5	AS-PN005_50 µM	36980	461071	87605	0.000000916	0.30
6	AS-PN005_100 µM	24222	498790	165688	0.000000293	0.09

List	Treatment	p-Akt	Akt	GAPDH	p-Akt expression	
					p-Akt / Akt / GAPDH	Fold expression
1	DMSO	12504	147078.5	81682	0.000001041	1.00
2	Erlotinib	6362	171891	86757	0.000000427	0.41
3	AS-PN005_12.5 µM	9672	176531	105440	0.000000520	0.50
4	AS-PN005_25 µM	7571	199178	92456	0.000000411	0.40
5	AS-PN005_50 µM	5552	185121	87605	0.000000342	0.33
6	AS-PN005_100 µM	4297	193334.5	165688	0.000000134	0.13

Batch 2

List	Treatment	p-ERK	ERK	GAPDH	p-ERK expression	
					p-ERK / ERK / GAPDH	Fold expression
1	DMSO	303508	108837	89574	0.000031132	1.00
2	Erlotinib	87341	112331	88411	0.000008795	0.28
3	AS-PN005_12.5 µM	120649	102836	96547	0.000012152	0.39
4	AS-PN005_25 µM	97315	90673	93457	0.000011484	0.37
5	AS-PN005_50 µM	76530	91913	105478	0.000007894	0.25
6	AS-PN005_100 µM	22406	77258	135346	0.000002143	0.07

List	Treatment	p-Akt	Akt	GAPDH	p-Akt expression	
					p-Akt / Akt / GAPDH	Fold expression
1	DMSO	141672	137078	89574	0.000011538	1.00
2	Erlotinib	49833	131891	88411	0.000004274	0.37
3	AS-PN005_12.5 µM	86634	188371	96547	0.000004764	0.41
4	AS-PN005_25 µM	85401	219178	93457	0.000004169	0.36
5	AS-PN005_50 µM	53637	145121	105478	0.000003504	0.30
6	AS-PN005_100 µM	42026	253334	135346	0.000001226	0.11

Batch 3

List	Treatment	p-ERK	ERK	GAPDH	p-ERK expression	
					p-ERK / ERK / GAPDH	Fold expression
1	DMSO	289547	180238	112487	0.000014281	1.00
2	Erlotinib	112483	225711	126258	0.000003947	0.28
3	AS-PN005_12.5 µM	305781	245478	185734	0.000006707	0.47
4	AS-PN005_25 µM	258862	248745	164587	0.000006323	0.44
5	AS-PN005_50 µM	133711	279658	100289	0.000004767	0.33
6	AS-PN005_100 µM	102784	422031	116427	0.000002092	0.15

List	Treatment	p-Akt	Akt	GAPDH	p-Akt expression	
					p-Akt / Akt / GAPDH	Fold expression
1	DMSO	426387	13865	112487	0.000273389	1.00
2	Erlotinib	131356	12697	126258	0.000081939	0.30
3	AS-PN005_12.5 µM	409871	17956	185734	0.000122898	0.45
4	AS-PN005_25 µM	418945	21965	164587	0.000115886	0.42
5	AS-PN005_50 µM	188741	18341	100289	0.000102610	0.38
6	AS-PN005_100 µM	192572	29387	116427	0.000056284	0.21

Average of p-ERK expression (fold)

List	Treatment	Batch 1	Batch 2	Batch 3	Mean	SD	SEM
1	DMSO	1.00	1.00	1.00	1.000	0.000	0.000
2	Erlotinib	0.30	0.28	0.28	0.287	0.013	0.007
3	AS-PN005_12.5 µM	0.42	0.39	0.47	0.427	0.040	0.023
4	AS-PN005_25 µM	0.39	0.37	0.44	0.402	0.038	0.022
	AS-PN005_50 µM	0.30	0.25	0.33	0.294	0.040	0.023
	AS-PN005_100 µM	0.09	0.07	0.15	0.103	0.040	0.023

Average of p-Akt expression (fold)

List	Treatment	Batch 1	Batch 2	Batch 3	Mean	SD	SEM
1	DMSO	1.00	1.00	1.00	1.000	0.000	0.000
2	Erlotinib	0.41	0.37	0.30	0.360	0.056	0.032
3	AS-PN005_12.5 µM	0.50	0.41	0.45	0.454	0.043	0.025
4	AS-PN005_25 µM	0.40	0.36	0.42	0.393	0.031	0.018
	AS-PN005_50 µM	0.33	0.30	0.38	0.336	0.036	0.021
	AS-PN005_100 µM	0.13	0.11	0.21	0.147	0.052	0.030

Original pictures for western blot analysis - Figure 7B

p-ERK

Batch 1

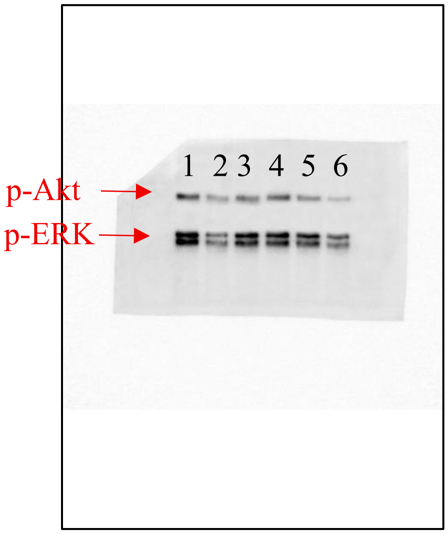

Batch 2

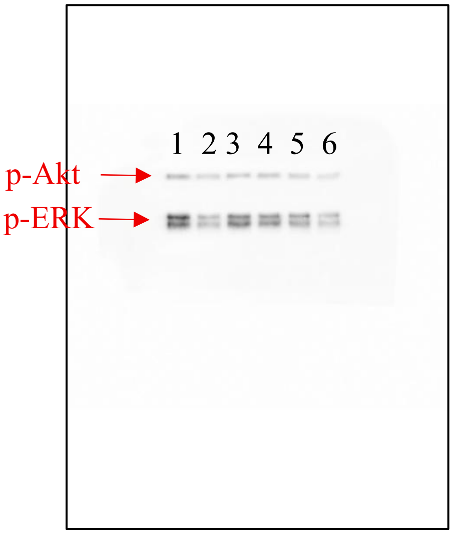

Batch 3

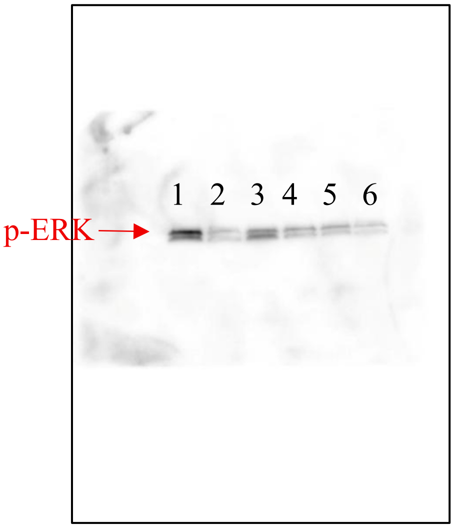

ERK

Batch 1

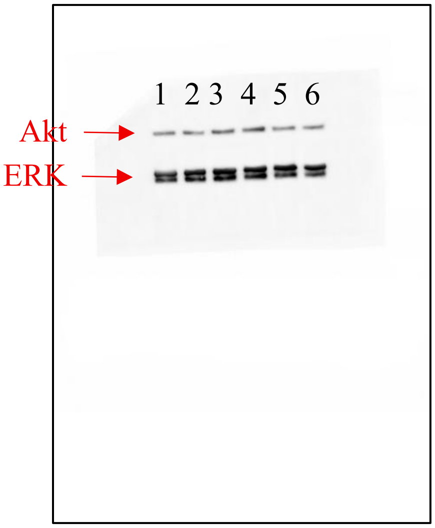

Batch 2

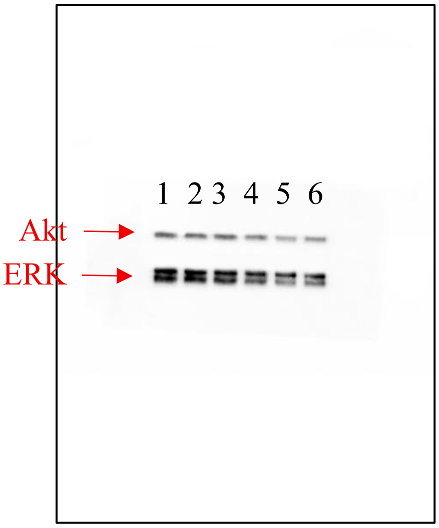

Batch 3

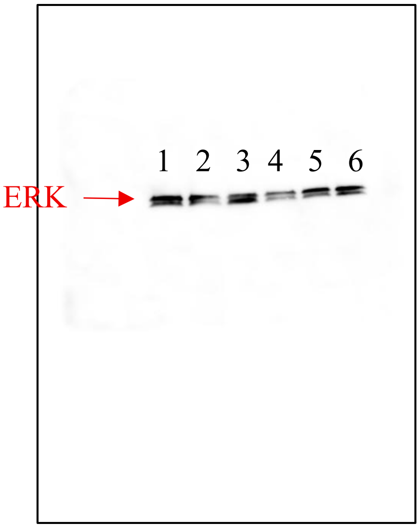

p-Akt

Batch 1 (same pic. as p-ERK)

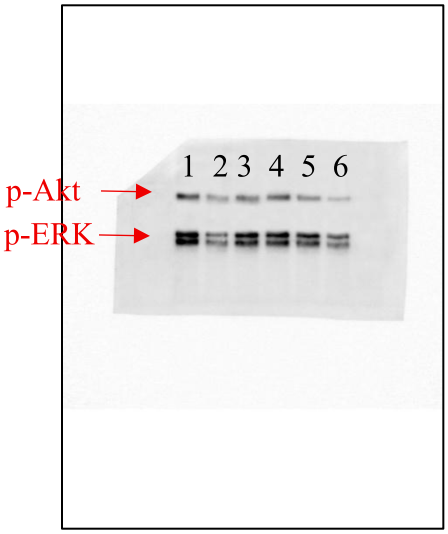

Batch 2 (same pic. as p-ERK)

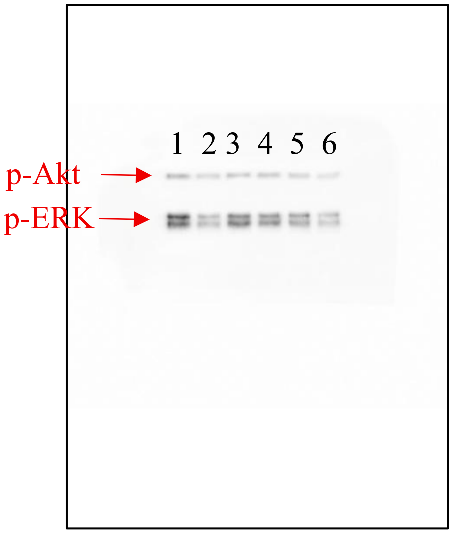

Batch 3

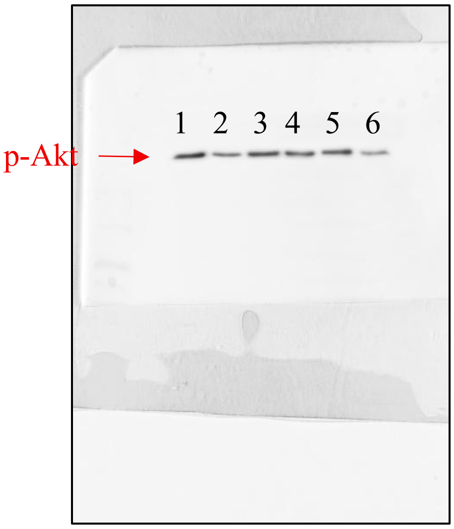

Akt

Batch 1 (same pic. as ERK)

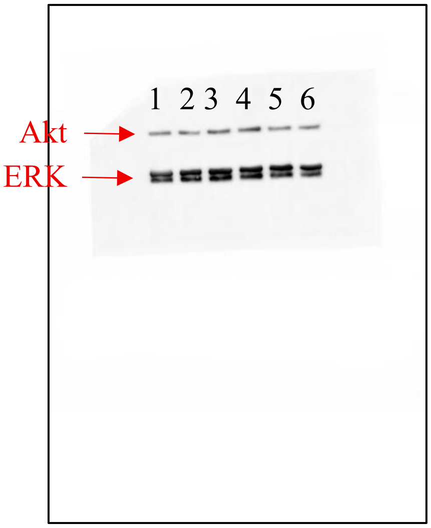

Batch 2 (same pic. as ERK)

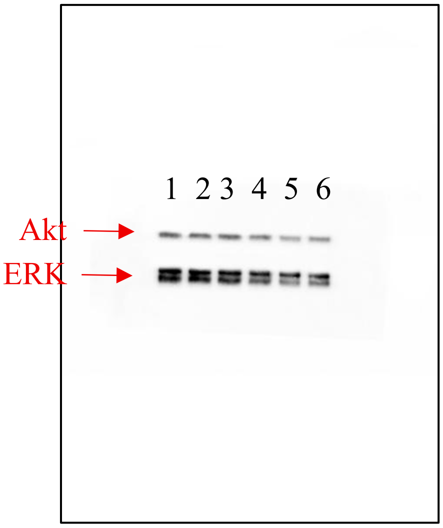

Batch 3

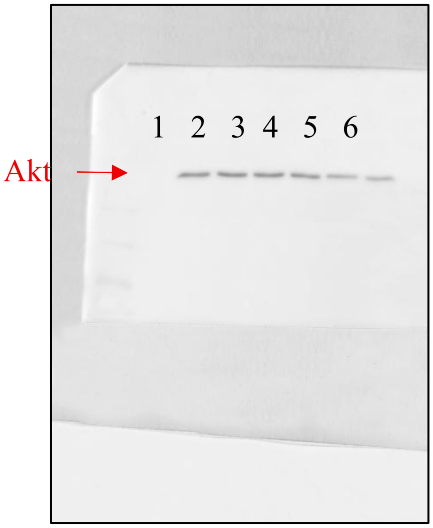

GAPDH

Batch 1

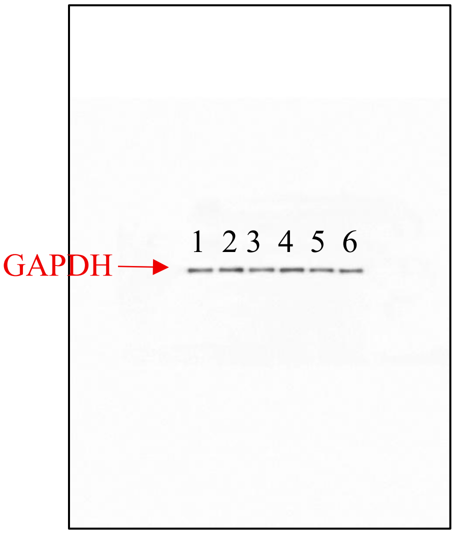

Batch 2

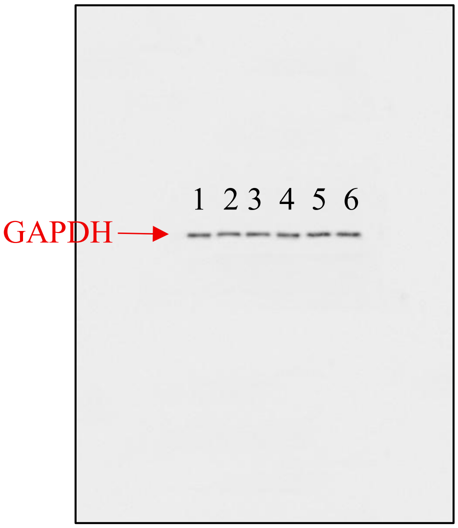

Batch 3

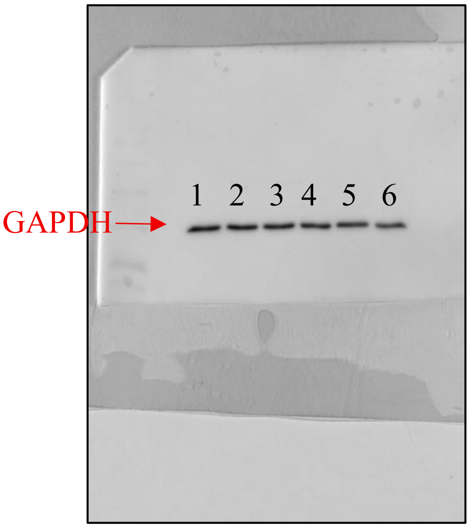

Batch 1

List	Treatment	p-ERK	ERK	GAPDH	p-ERK expression	
					p-ERK / ERK / GAPDH	Fold expression
1	DMSO	413053	417562	176482	5.60511E-06	1.00
2	Osimertinib	131746	439291	174113	1.72248E-06	0.31
3	AS-PN005_6.25 µM	256096	452041	143388	3.95105E-06	0.70
4	AS-PN005_12.5 µM	246111	404661	184564	3.29528E-06	0.59
5	AS-PN005_25 µM	188319	417325	140300	3.21634E-06	0.57
6	AS-PN005_50 µM	134756	435776	130960	2.36127E-06	0.42

List	Treatment	p-Akt	Akt	GAPDH	p-Akt expression	
					p-Akt / Akt / GAPDH	Fold expression
1	DMSO	98424	125334	176482	4.44971E-06	1.00
2	Osimertinib	38556	119427	174113	1.85421E-06	0.42
3	AS-PN005_6.25 µM	79126	169630	143388	3.25315E-06	0.73
4	AS-PN005_12.5 µM	118895	213149	184564	3.02227E-06	0.68
5	AS-PN005_25 µM	63779	165069	140300	2.75394E-06	0.62
6	AS-PN005_50 µM	40599	161065	130960	1.92476E-06	0.43

Batch 2

List	Treatment	p-ERK	ERK	GAPDH	p-ERK expression	
					p-ERK / ERK / GAPDH	Fold expression
1	DMSO	238011	235459	136351	7.4135E-06	1.00
2	Osimertinib	94791	267968	124120	2.84998E-06	0.38
3	AS-PN005_6.25 µM	198164	250906	137405	5.74793E-06	0.78
4	AS-PN005_12.5 µM	159540	262594	133947	4.53578E-06	0.61
5	AS-PN005_25 µM	141891	259868	137186	3.98008E-06	0.54
6	AS-PN005_50 µM	120373	214668	158281	3.54269E-06	0.48

List	Treatment	p-Akt	Akt	GAPDH	p-Akt expression	
					p-Akt / Akt / GAPDH	Fold expression
1	DMSO	414804	356267	136351	8.53904E-06	1.00
2	Osimertinib	181842	350452	124120	4.18046E-06	0.49
3	AS-PN005_6.25 µM	288014	339970	137405	6.16553E-06	0.72
4	AS-PN005_12.5 µM	248986	311833	133947	5.96101E-06	0.70
5	AS-PN005_25 µM	206370	275202	137186	5.4662E-06	0.64
6	AS-PN005_50 µM	170834	292648	158281	3.68808E-06	0.43

Batch 3

List	Treatment	p-ERK	ERK	GAPDH	p-ERK expression	
					p-ERK / ERK / GAPDH	Fold expression
1	DMSO	352897	754896	385649	1.21218E-06	1.00
2	Osimertinib	110256	723156	375487	4.06046E-07	0.33
3	AS-PN005_6.25 µM	270589	737311	385974	9.50827E-07	0.78
4	AS-PN005_12.5 µM	241478	709865	395781	8.59502E-07	0.71
5	AS-PN005_25 µM	195648	735842	374125	7.1068E-07	0.59
6	AS-PN005_50 µM	153874	758942	352986	5.7438E-07	0.47

List	Treatment	p-Akt	Akt	GAPDH	p-Akt expression	
					p-Akt / Akt / GAPDH	Fold expression
1	DMSO	125846	542156	385649	6.01898E-07	1.00
2	Osimertinib	45112	558712	375487	2.15035E-07	0.36
3	AS-PN005_6.25 µM	108753	594582	385974	4.73883E-07	0.79
4	AS-PN005_12.5 µM	88124	521547	395781	4.26919E-07	0.71
5	AS-PN005_25 µM	64821	518745	374125	3.33999E-07	0.55
6	AS-PN005_50 µM	43256	503327	352986	2.43466E-07	0.40

Average of p-ERK expression (fold)

List	Treatment	Batch 1	Batch 2	Batch 3	Mean	SD	SEM
1	DMSO	1.00	1.00	1.00	1.000	0.000	0.000
2	Osimertinib	0.31	0.38	0.33	0.342	0.039	0.028
3	AS-PN005_6.25 µM	0.70	0.78	0.78	0.755	0.044	0.031
4	AS-PN005_12.5 µM	0.59	0.61	0.71	0.636	0.064	0.045
	AS-PN005_25 µM	0.57	0.54	0.59	0.566	0.026	0.018
	AS-PN005_50 µM	0.42	0.48	0.47	0.458	0.032	0.022

Average of p-Akt expression (fold)

List	Treatment	Batch 1	Batch 2	Batch 3	Mean	SD	SEM
1	DMSO	1.00	1.00	1.00	1.000	0.000	0.000
2	Osimertinib	0.42	0.49	0.36	0.421	0.066	0.047
3	AS-PN005_6.25 µM	0.73	0.72	0.79	0.747	0.035	0.025
4	AS-PN005_12.5 µM	0.68	0.70	0.71	0.696	0.015	0.011
	AS-PN005_25 µM	0.62	0.64	0.55	0.605	0.044	0.031
	AS-PN005_50 µM	0.43	0.43	0.40	0.423	0.016	0.011

Original pictures for western blot analysis - Figure 8A

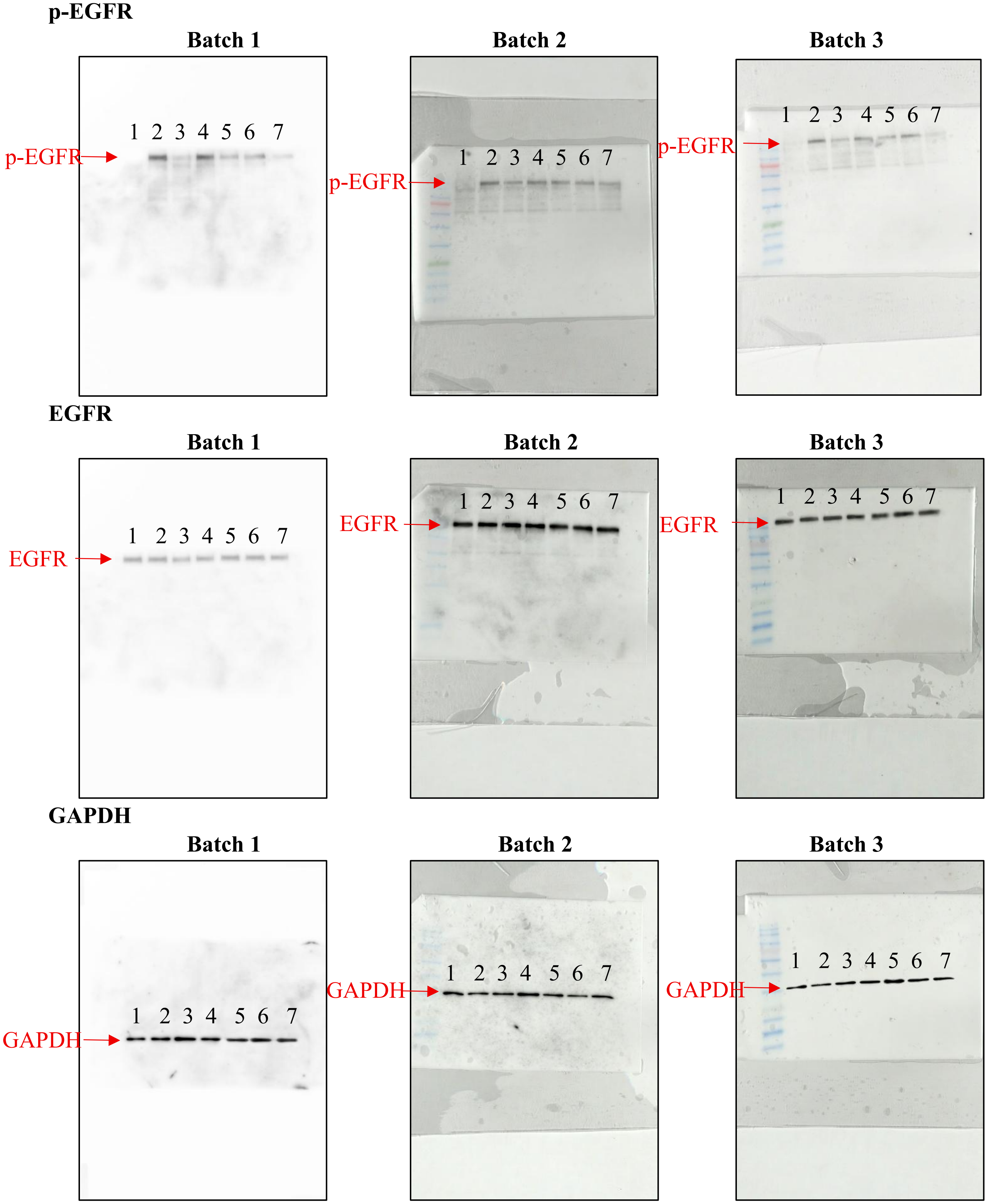

Note: 1 = 0.2%DMSO (-) 2 = 0.2%DMSO (+) 3 = Erlotinib
4 = 12.5 µM of AS-PN005(+) 5 = 25 µM of AS-PN005(+) 6 = 50 µM of AS-PN005(+)
7 = 100 µM of AS-PN005(+)

Batch 1

List	Treatment	p-EGFR	EGFR	GAPDH	p-EGFR expression	
					p-EGFR / EGFR / GAPDH	Fold expression
1	DMSO (-)	3214972	90763	56629700	6.25495E-07	0.08
2	DMSO (+)	32992728	76859	56629700	7.58018E-06	1.00
3	Erlotinib (+)	12501476	61543	64627186	3.14317E-06	0.41
4	AS-PN005_12.5 µM (+)	26099984	71940	56117964	6.46499E-06	0.85
5	AS-PN005_25 µM (+)	13731488	94586	56629700	2.56358E-06	0.34
6	AS-PN005_50 µM (+)	14081836	103132	64627186	2.11276E-06	0.28
7	AS-PN005_100 µM (+)	8583894	96420	53117964	1.67601E-06	0.22

Batch 2

List	Treatment	p-EGFR	EGFR	GAPDH	p-EGFR expression	
					p-EGFR / EGFR / GAPDH	Fold expression
1	DMSO (-)	27715	90763	361391	8.44946E-07	0.15
2	DMSO (+)	132340	76859	311391	5.52956E-06	1.00
3	Erlotinib (+)	61581	61543	339846	2.94433E-06	0.53
4	AS-PN005_12.5 µM (+)	216652	151940	381391	3.7387E-06	0.68
5	AS-PN005_25 µM (+)	97694	114586	316113	2.69708E-06	0.49
6	AS-PN005_50 µM (+)	76339	133132	253774	2.25952E-06	0.41
7	AS-PN005_100 µM (+)	53924	106420	331319	1.52937E-06	0.28

Batch 3

List	Treatment	p-EGFR	EGFR	GAPDH	p-EGFR expression	
					p-EGFR / EGFR / GAPDH	Fold expression
1	DMSO (-)	8885	311242	281391	1.01449E-07	0.08
2	DMSO (+)	102996	298530	281391	1.22609E-06	1.00
3	Erlotinib (+)	51684	281708	309846	5.92122E-07	0.48
4	AS-PN005_12.5 µM (+)	93367	349752	301391	8.85733E-07	0.72
5	AS-PN005_25 µM (+)	56797	355860	336113	4.74855E-07	0.39
6	AS-PN005_50 µM (+)	56539	393160	303774	4.734E-07	0.39
7	AS-PN005_100 µM (+)	15798	328658	301391	1.59488E-07	0.13

Average of p-EGFR expression (fold)

List	Treatment	Batch 1	Batch 2	Batch 3	Mean	SD	SEM
1	DMSO (-)	0.08	0.15	0.08	0.11	0.04	0.02
2	DMSO (+)	1.00	1.00	1.00	1.00	0.00	0.00
3	Erlotinib (+)	0.41	0.53	0.48	0.48	0.06	0.03
4	AS-PN005_12.5 µM (+)	0.85	0.68	0.72	0.75	0.09	0.05
5	AS-PN005_25 µM (+)	0.34	0.49	0.39	0.40	0.08	0.04
6	AS-PN005_50 µM (+)	0.28	0.41	0.39	0.36	0.07	0.04
7	AS-PN005_100 µM (+)	0.22	0.28	0.13	0.21	0.07	0.04

(-) represents the non-stimulated with human EGF before cell harvesting.

(+) represents the cells were stimulated with human EGF before cell harvesting.

Original pictures for western blot analysis - Figure 8B

p-EGFR

Batch 1

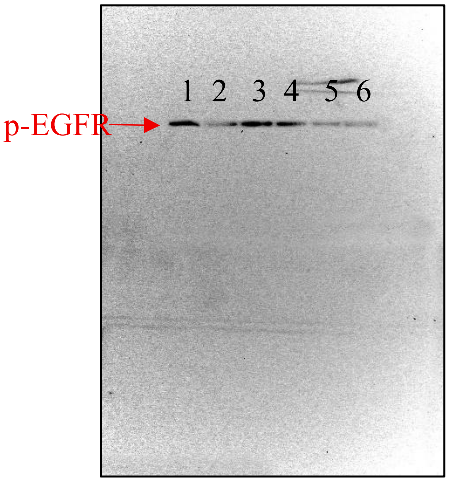

Batch 2

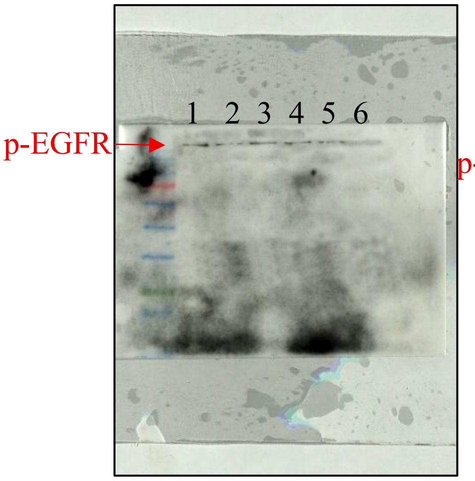

Batch 3

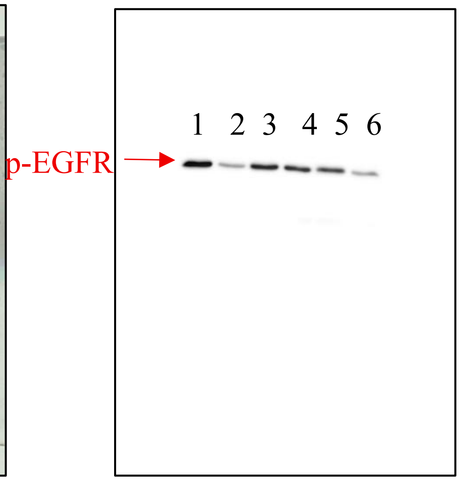

EGFR

Batch 1

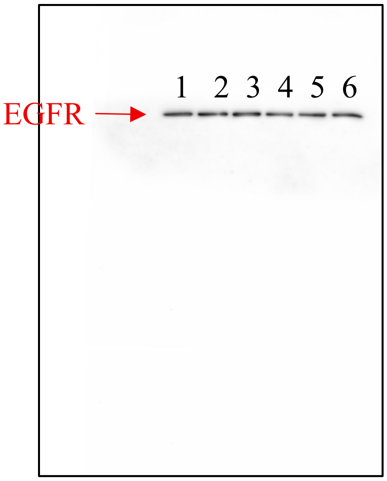

Batch 2

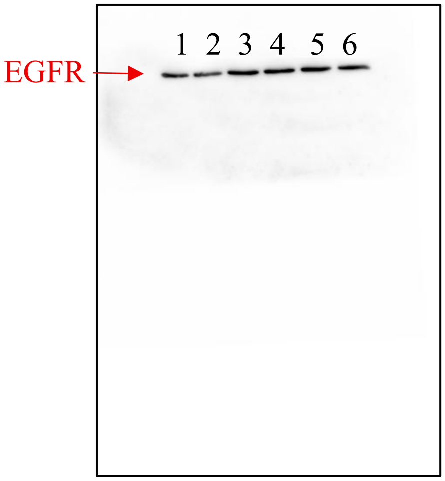

Batch 3

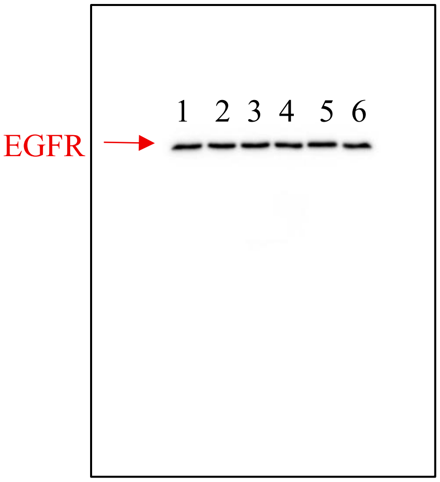

GAPDH

Batch 1

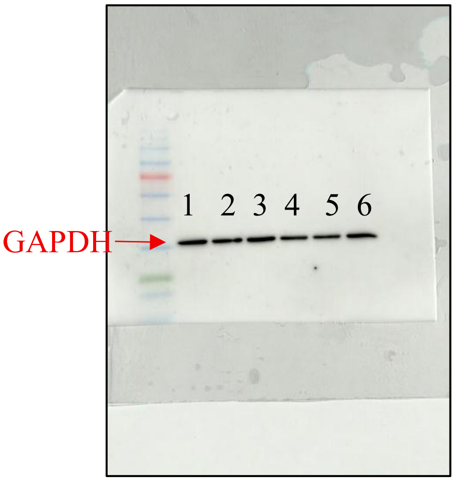

Batch 2

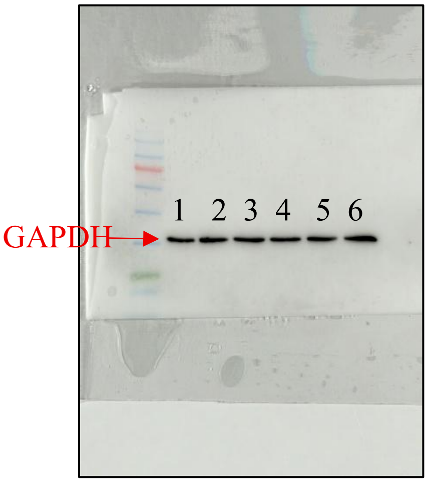

Batch 3

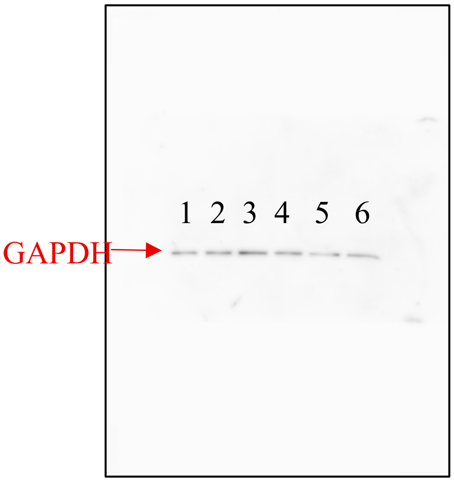

Batch 1

List	Treatment	p-EGFR	EGFR	GAPDH	p-EGFR expression	
					p-EGFR / EGFR / GAPDH	Fold expression
1	DMSO	259472	210104	176482	6.998E-06	1.00
2	Osimertinib	111443	215791	174113	2.966E-06	0.42
3	AS-PN005_6.25 µM	170066	215056	143388	5.515E-06	0.79
4	AS-PN005_12.5 µM	148643	184902	184564	4.356E-06	0.62
5	AS-PN005_25 µM	95094	173300	140300	3.911E-06	0.56
6	AS-PN005_50 µM	81345	228429	130960	2.719E-06	0.39

Batch 2

List	Treatment	p-EGFR	EGFR	GAPDH	p-EGFR expression	
					p-EGFR / EGFR / GAPDH	Fold expression
1	DMSO	134949	35338	136351	2.801E-05	1.00
2	Osimertinib	50023	38361	124120	1.051E-05	0.38
3	AS-PN005_6.25 µM	118559	37311	137405	2.313E-05	0.83
4	AS-PN005_12.5 µM	85326	34733	133947	1.834E-05	0.65
5	AS-PN005_25 µM	80346	35559	137186	1.647E-05	0.59
6	AS-PN005_50 µM	78293	39713	158281	1.246E-05	0.44

Batch 3

List	Treatment	p-EGFR	EGFR	GAPDH	p-EGFR expression	
					p-EGFR / EGFR / GAPDH	Fold expression
1	DMSO	355687	263862	385649	3.495E-06	1.00
2	Osimertinib	111443	261325	375487	1.136E-06	0.32
3	AS-PN005_6.25 µM	302564	253148	385974	3.097E-06	0.89
4	AS-PN005_12.5 µM	246745	260124	395781	2.397E-06	0.69
5	AS-PN005_25 µM	196325	268741	374125	1.953E-06	0.56
6	AS-PN005_50 µM	135899	244568	352986	1.574E-06	0.45

Average of p-EGFR expression (fold)

List	Treatment	Batch 1	Batch 2	Batch 3	Mean	SD	SEM
1	DMSO	1.00	1.00	1.00	1.000	0.000	0.000
2	Osimertinib	0.31	0.38	0.32	0.336	0.048	0.034
3	AS-PN005_6.25 µM	0.70	0.83	0.89	0.806	0.085	0.060
4	AS-PN005_12.5 µM	0.59	0.65	0.69	0.643	0.047	0.033
5	AS-PN005_25 µM	0.57	0.59	0.56	0.574	0.010	0.007
6	AS-PN005_50 µM	0.42	0.44	0.45	0.439	0.017	0.012
