# Supplementary figures and images for: Asiatic Acid from Centella asiatica as a Potent EGFR Tyrosine Kinase Inhibitor with Anticancer Activity in NSCLC Cells Harboring Wild-Type and T790M-Mutated EGFR
Source: Biomolecules. 2025 Oct 3;15(10):1410. doi: 10.3390/biom15101410 (PMC12563520; doi:10.3390/biom15101410)

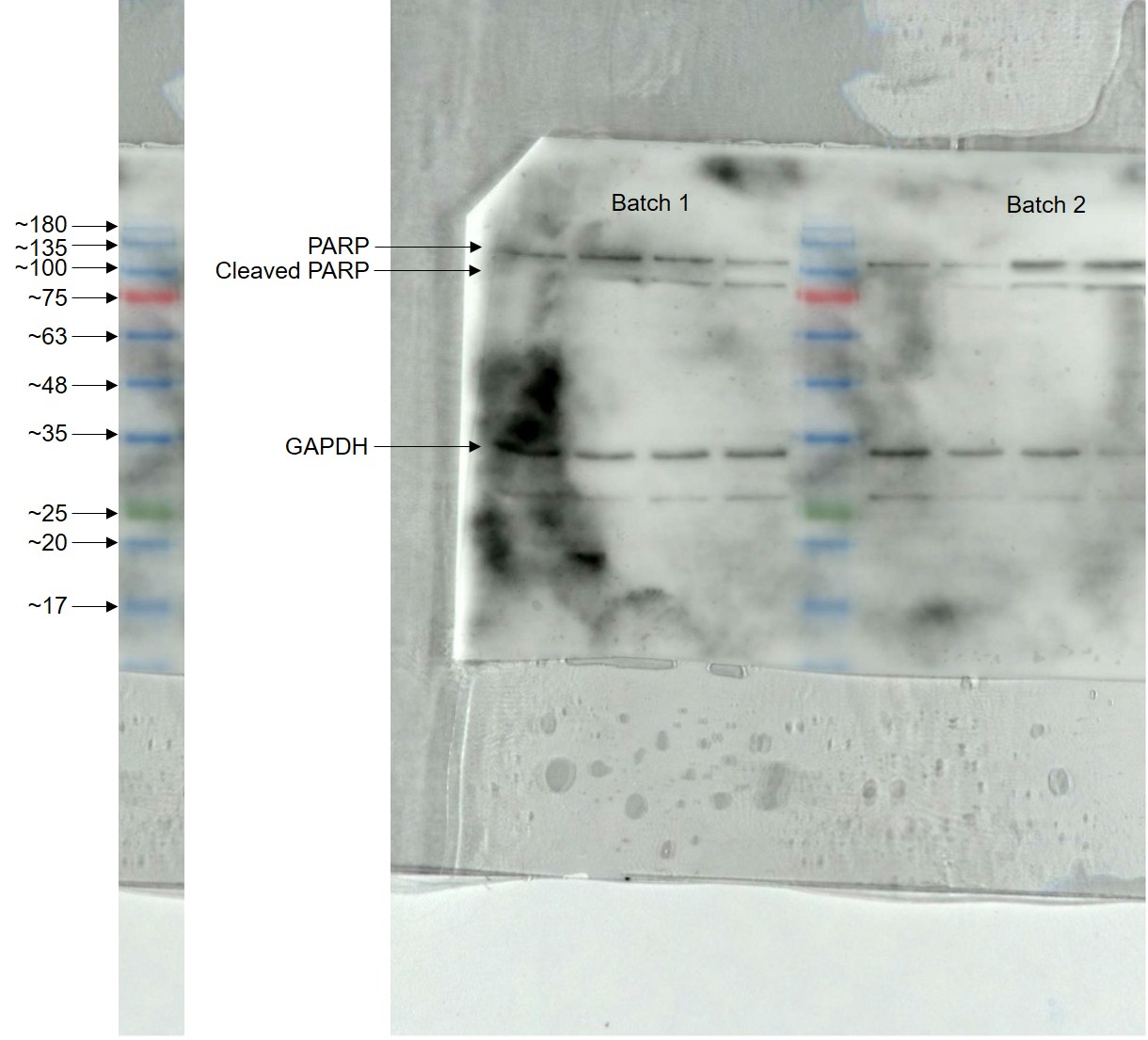

Supplement: Supplementary file 1 [file biomolecules-15-01410-s001.zip › Figure 6A_PARP_Cleaved PARP_GAPDH_Batch 1.jpg]

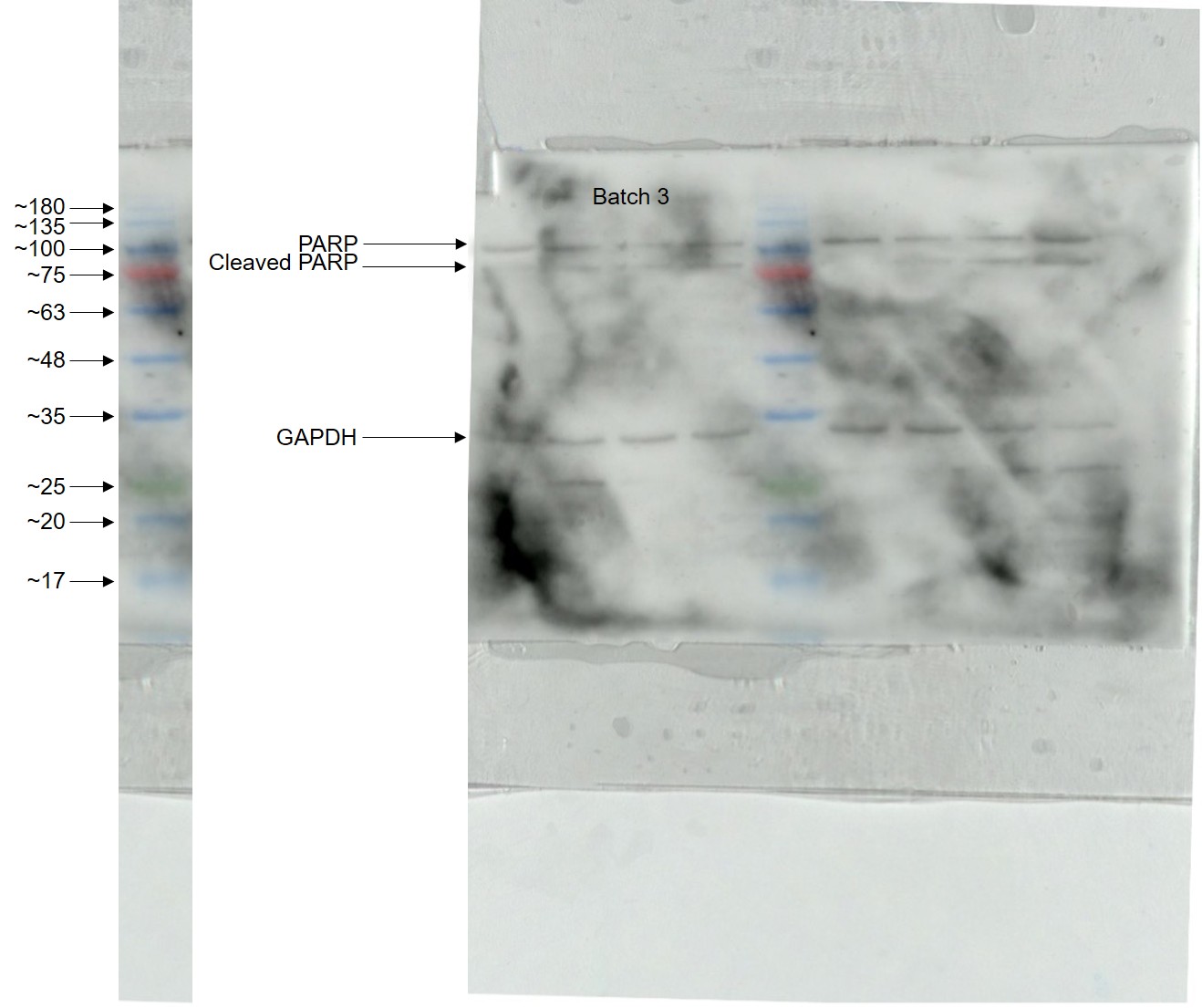

Supplement: Supplementary file 1 [file biomolecules-15-01410-s001.zip › Figure 6A_PARP_Cleaved PARP_GAPDH_Batch 3.jpg]

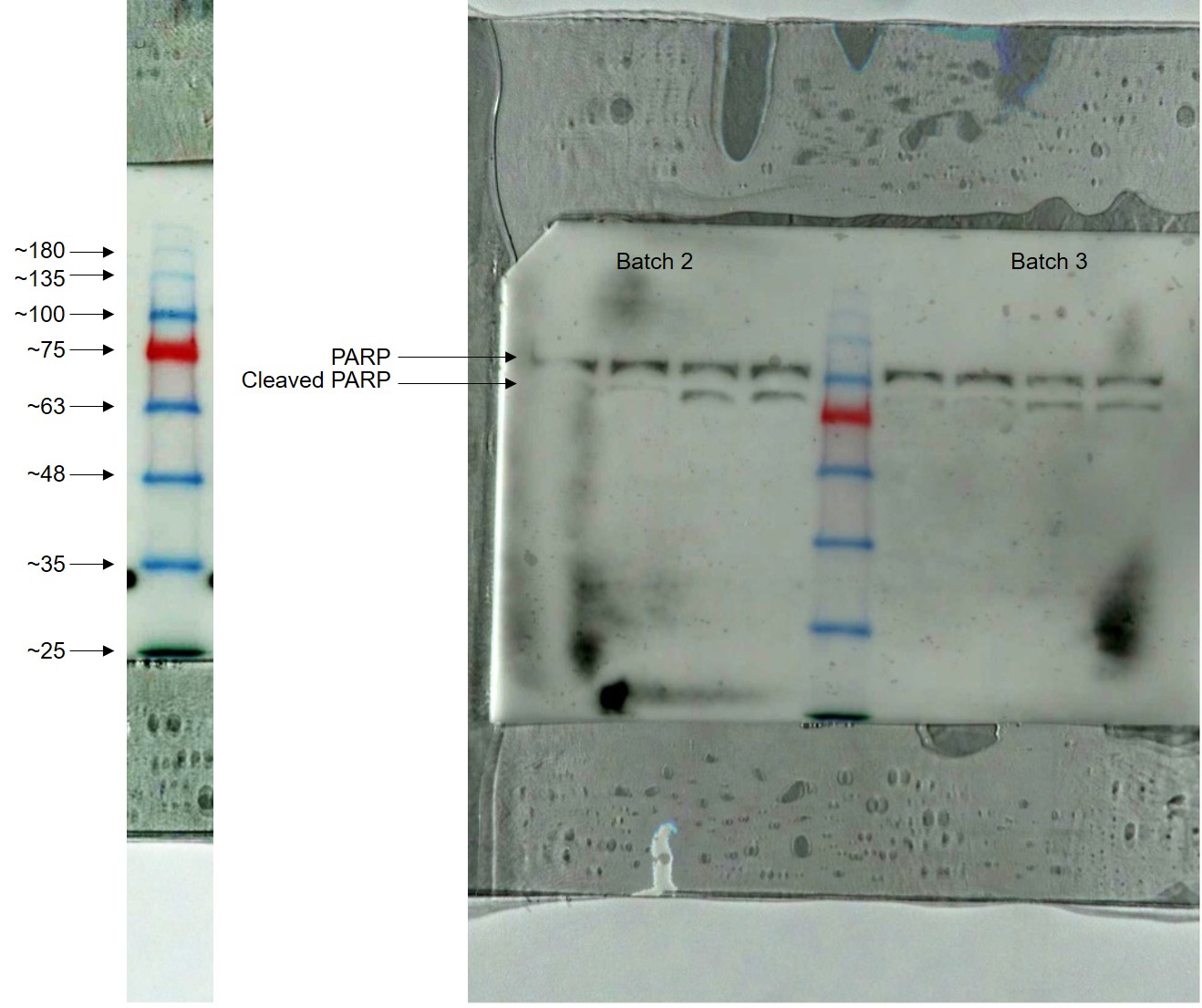

Supplement: Supplementary file 1 [file biomolecules-15-01410-s001.zip › Figure 6B_PARP_Cleaved PARP_Batch 2.jpg]

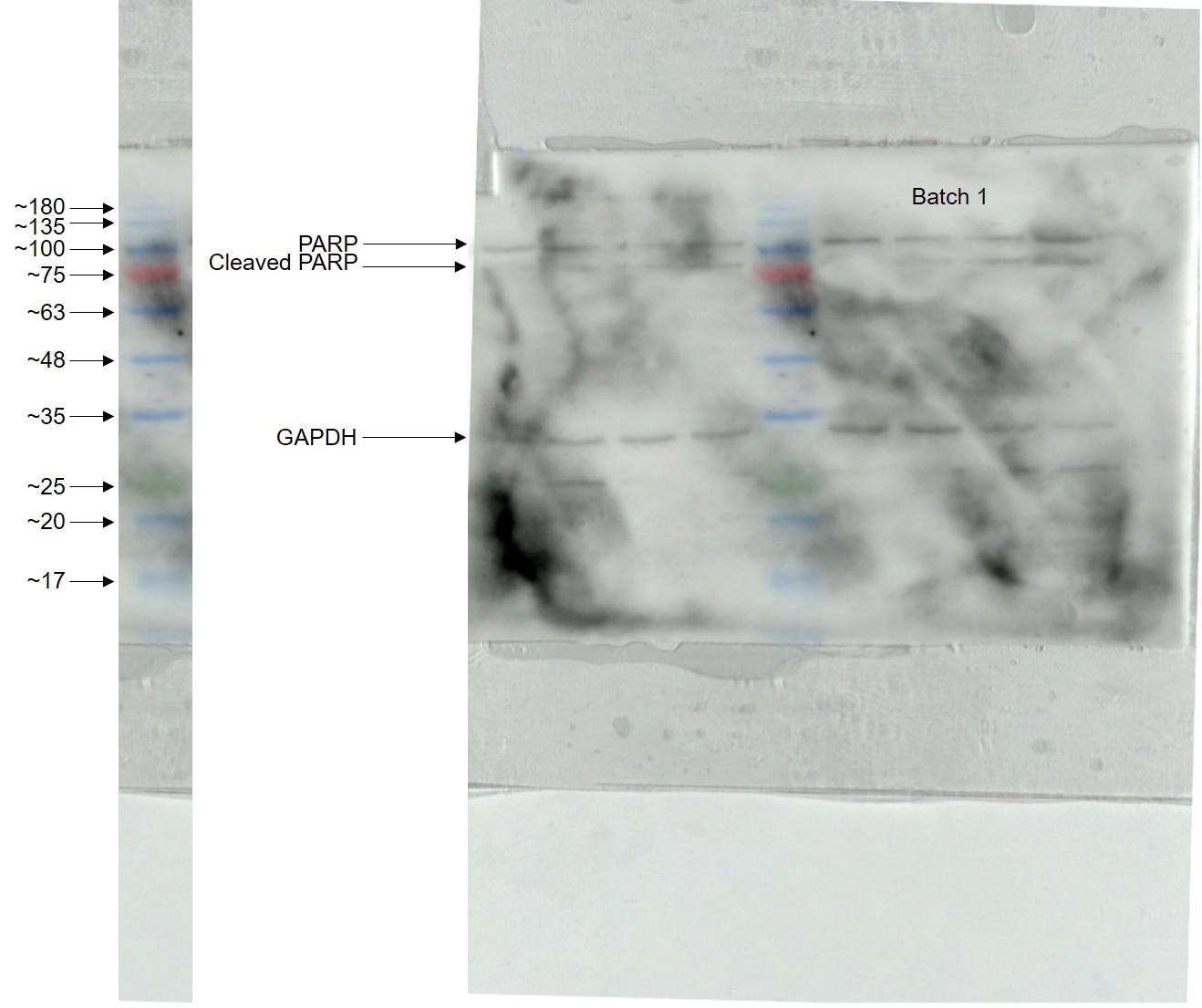

Supplement: Supplementary file 1 [file biomolecules-15-01410-s001.zip › Figure 6B_PARP_Cleaved PARP_GAPDH_Batch 1.jpg]

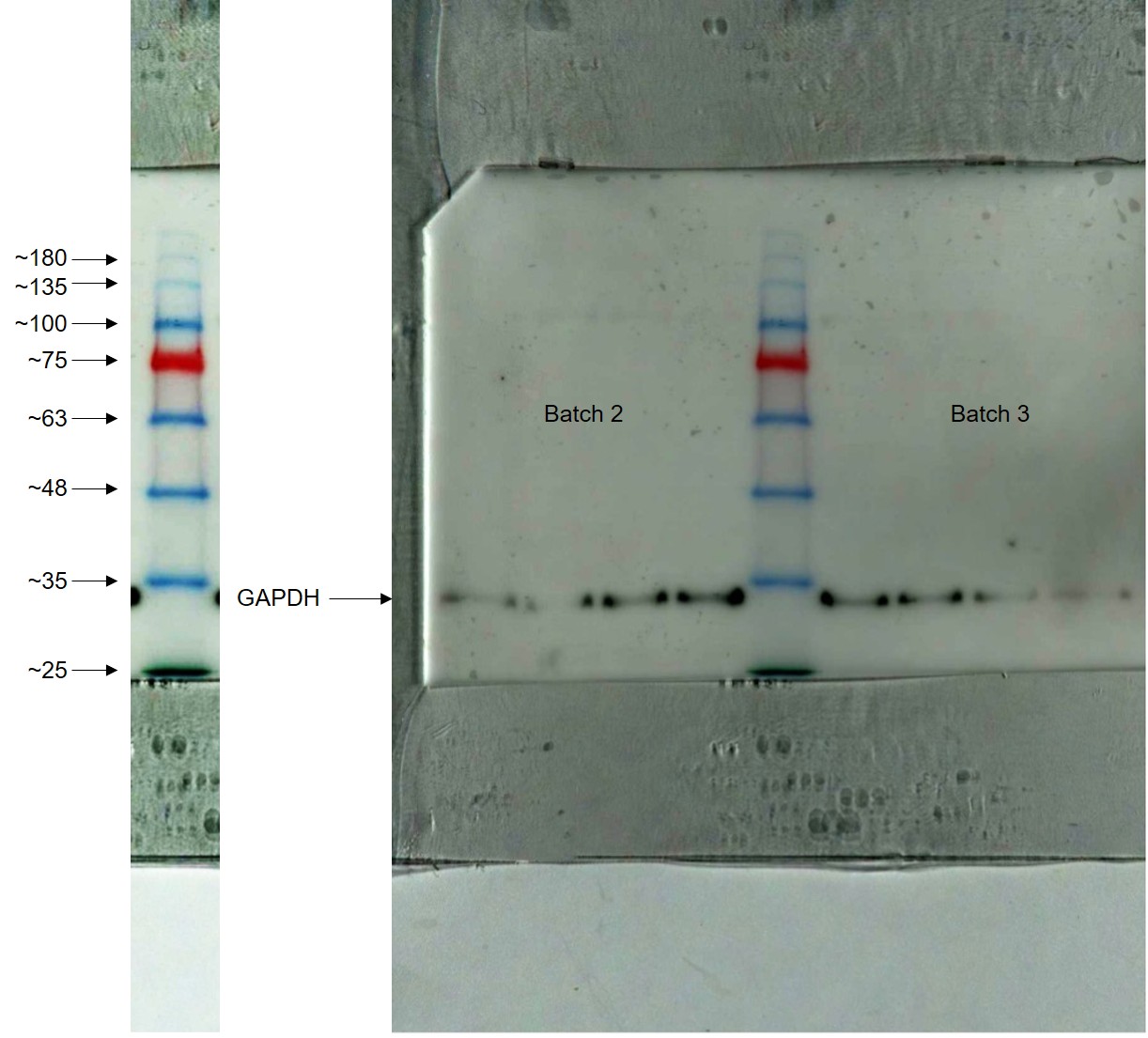

Supplement: Supplementary file 1 [file biomolecules-15-01410-s001.zip › Figure 6B_PARP_GAPDH_Batch 2.jpg]

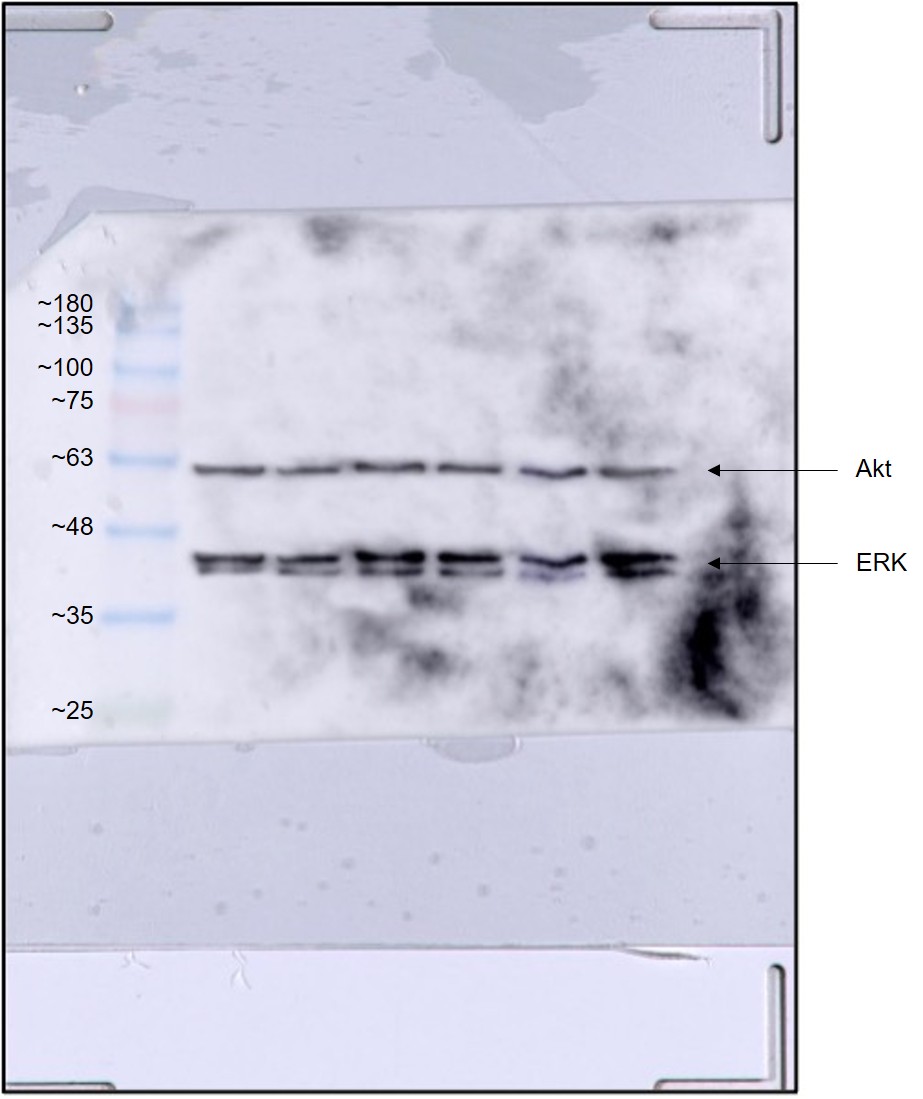

Supplement: Supplementary file 1 [file biomolecules-15-01410-s001.zip › Figure 7A_Akt_Batch 1.jpg]

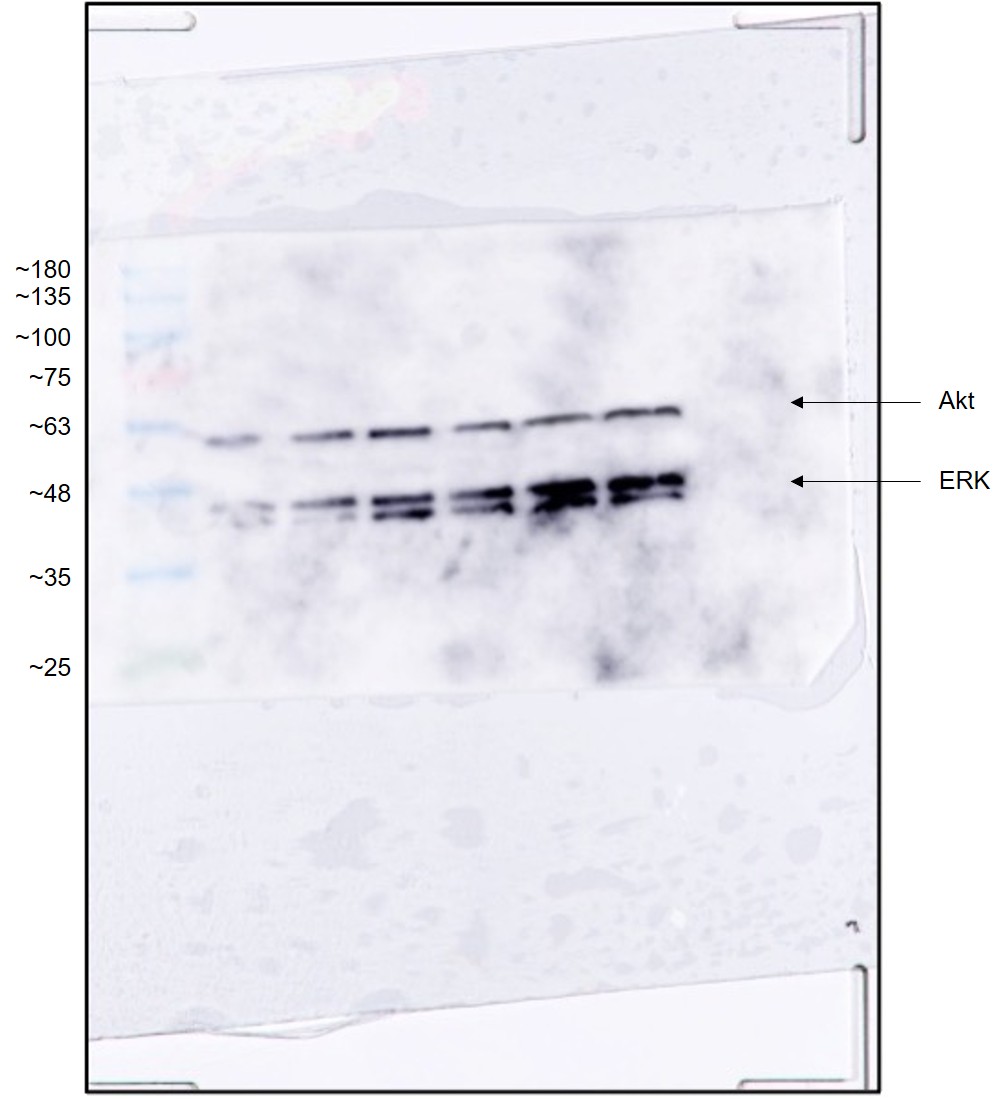

Supplement: Supplementary file 1 [file biomolecules-15-01410-s001.zip › Figure 7A_Akt_Batch 2.jpg]

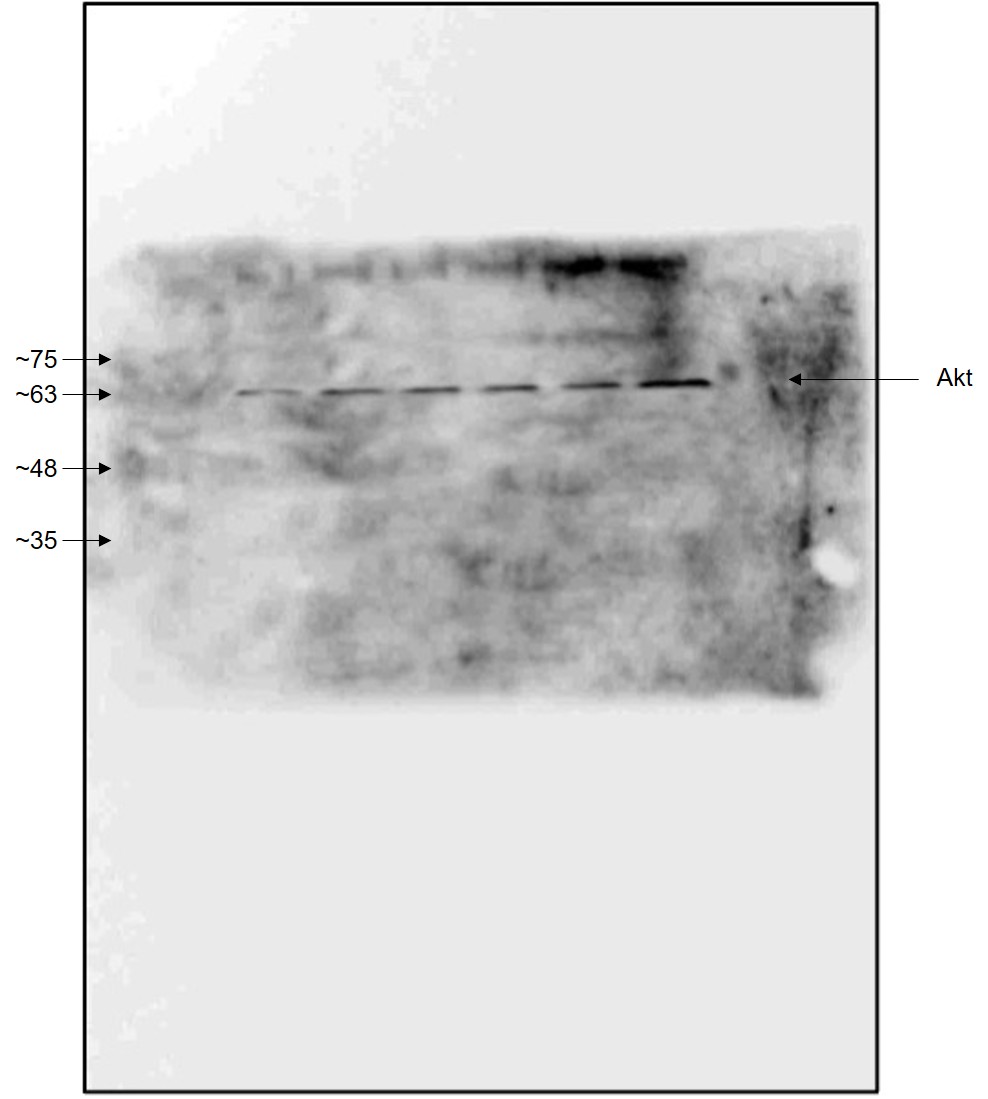

Supplement: Supplementary file 1 [file biomolecules-15-01410-s001.zip › Figure 7A_Akt_Batch 3.jpg]

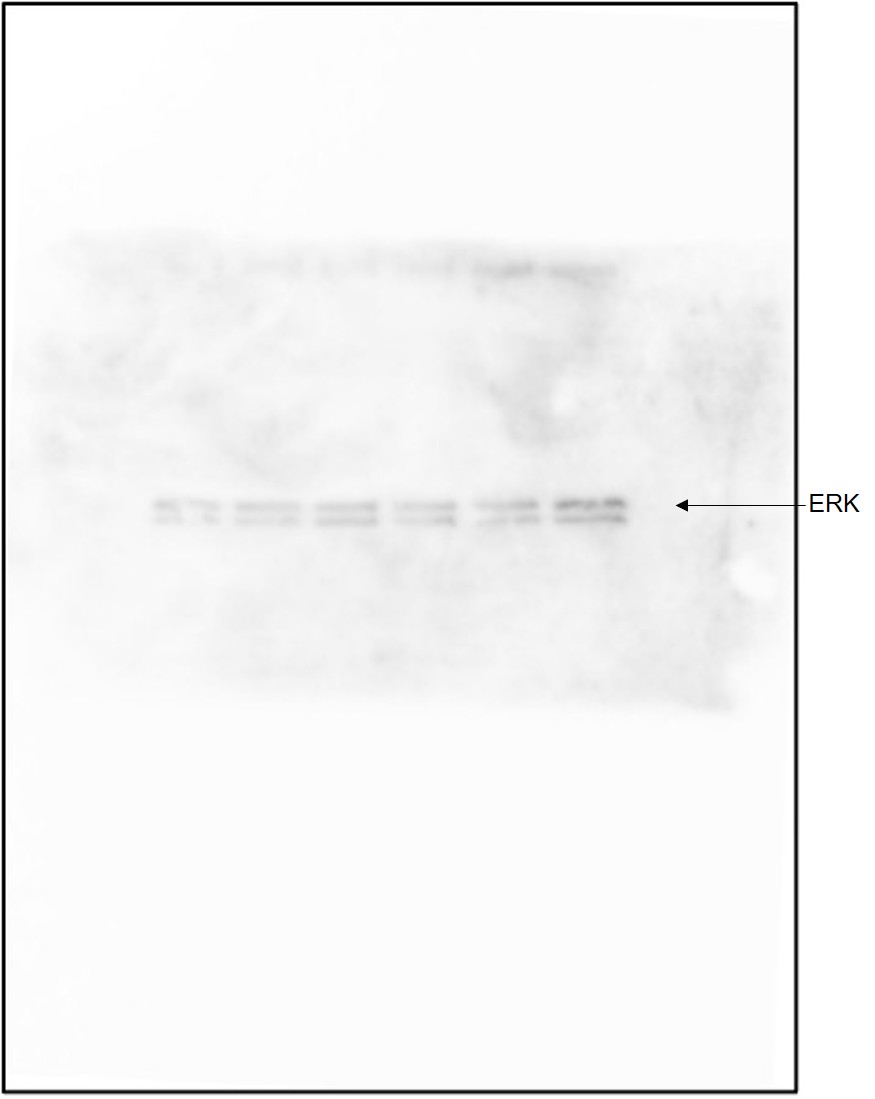

Supplement: Supplementary file 1 [file biomolecules-15-01410-s001.zip › Figure 7A_ERK_Batch 3.jpg]

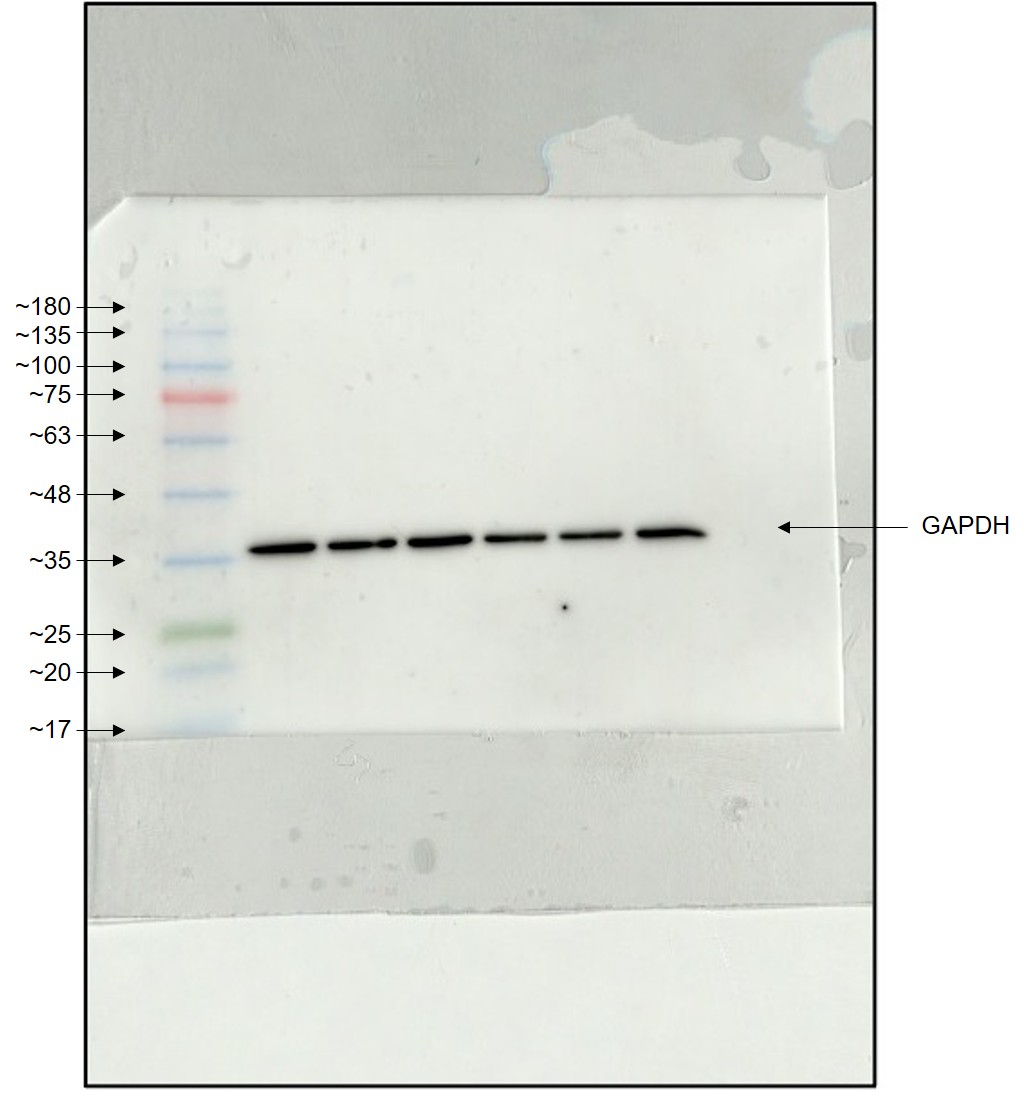

Supplement: Supplementary file 1 [file biomolecules-15-01410-s001.zip › Figure 7A_GAPDH_Batch 1.jpg]

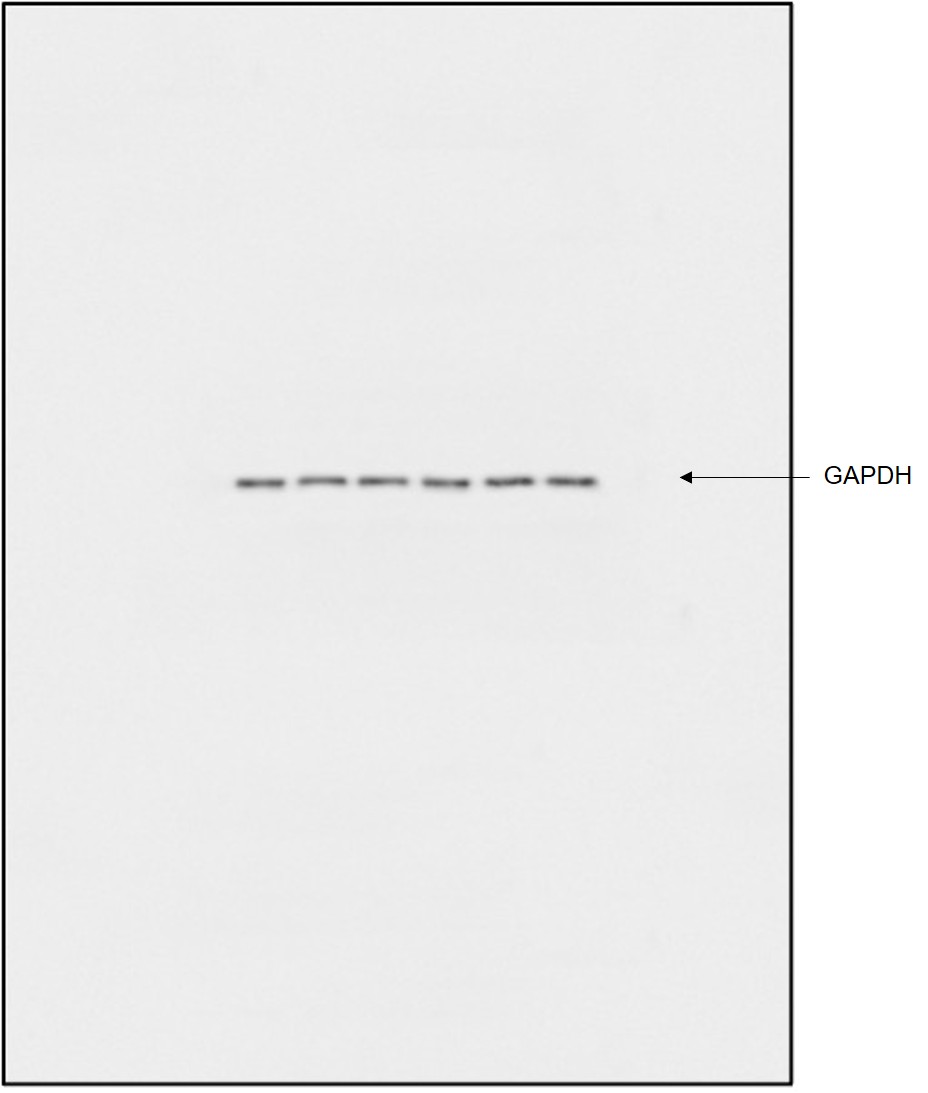

Supplement: Supplementary file 1 [file biomolecules-15-01410-s001.zip › Figure 7A_GAPDH_Batch 2.jpg]

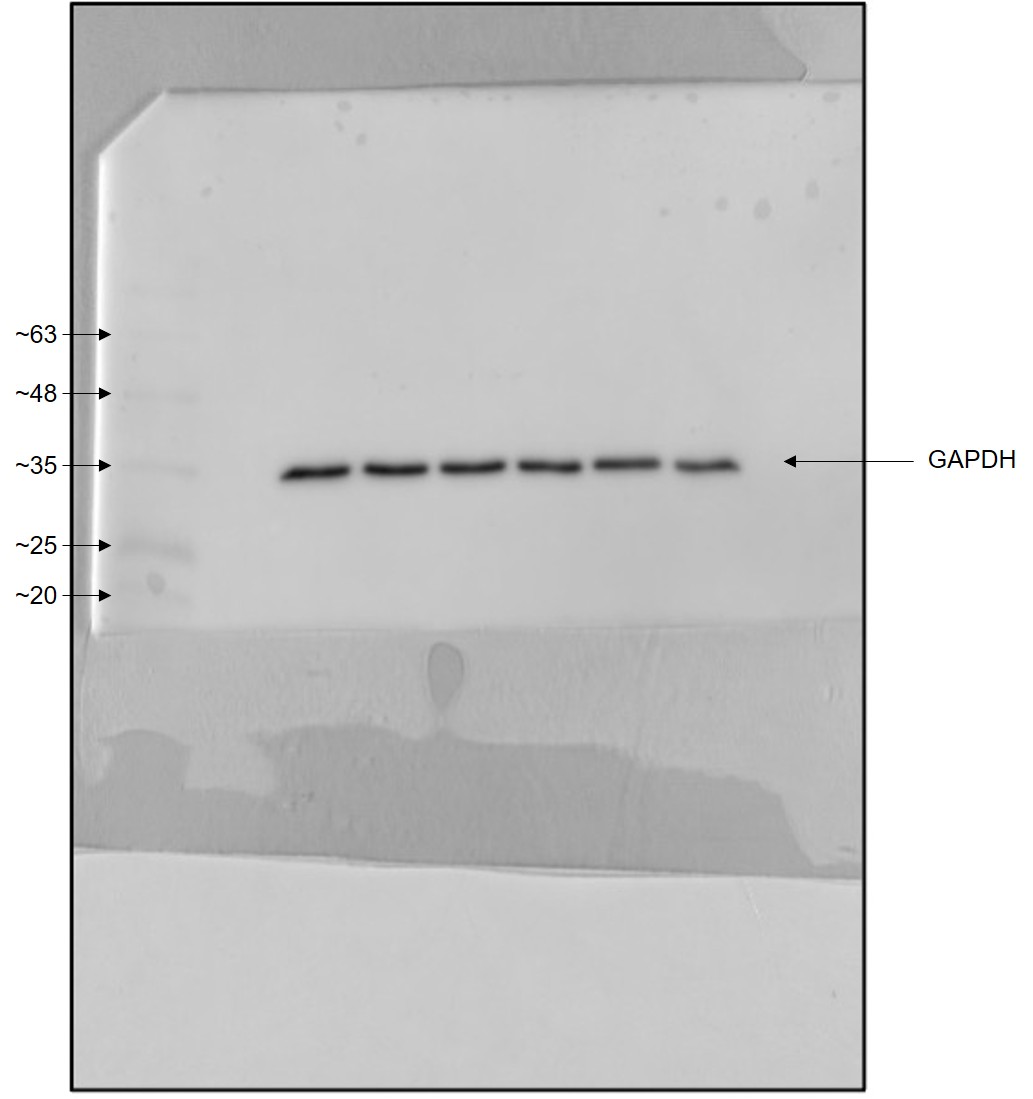

Supplement: Supplementary file 1 [file biomolecules-15-01410-s001.zip › Figure 7A_GAPDH_Batch 3.jpg]

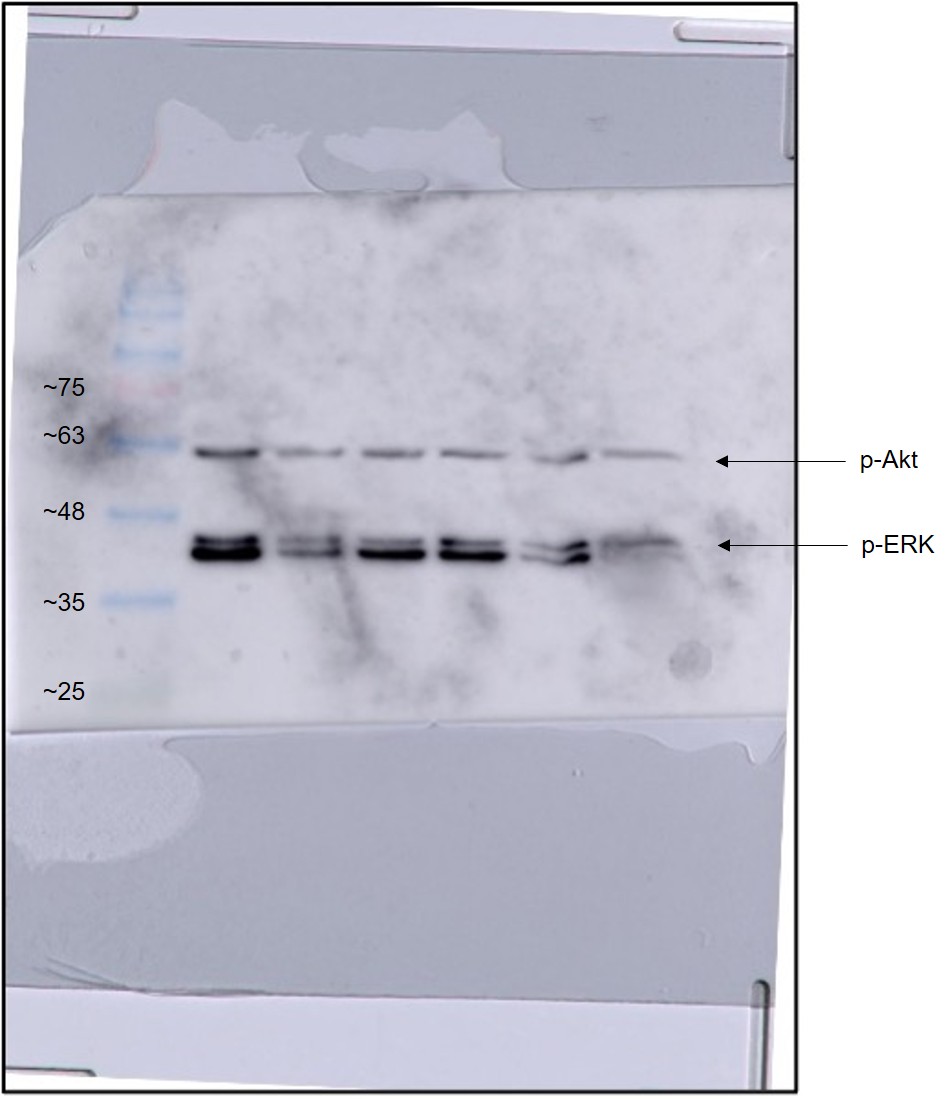

Supplement: Supplementary file 1 [file biomolecules-15-01410-s001.zip › Figure 7A_p-Akt_Batch 1.jpg]

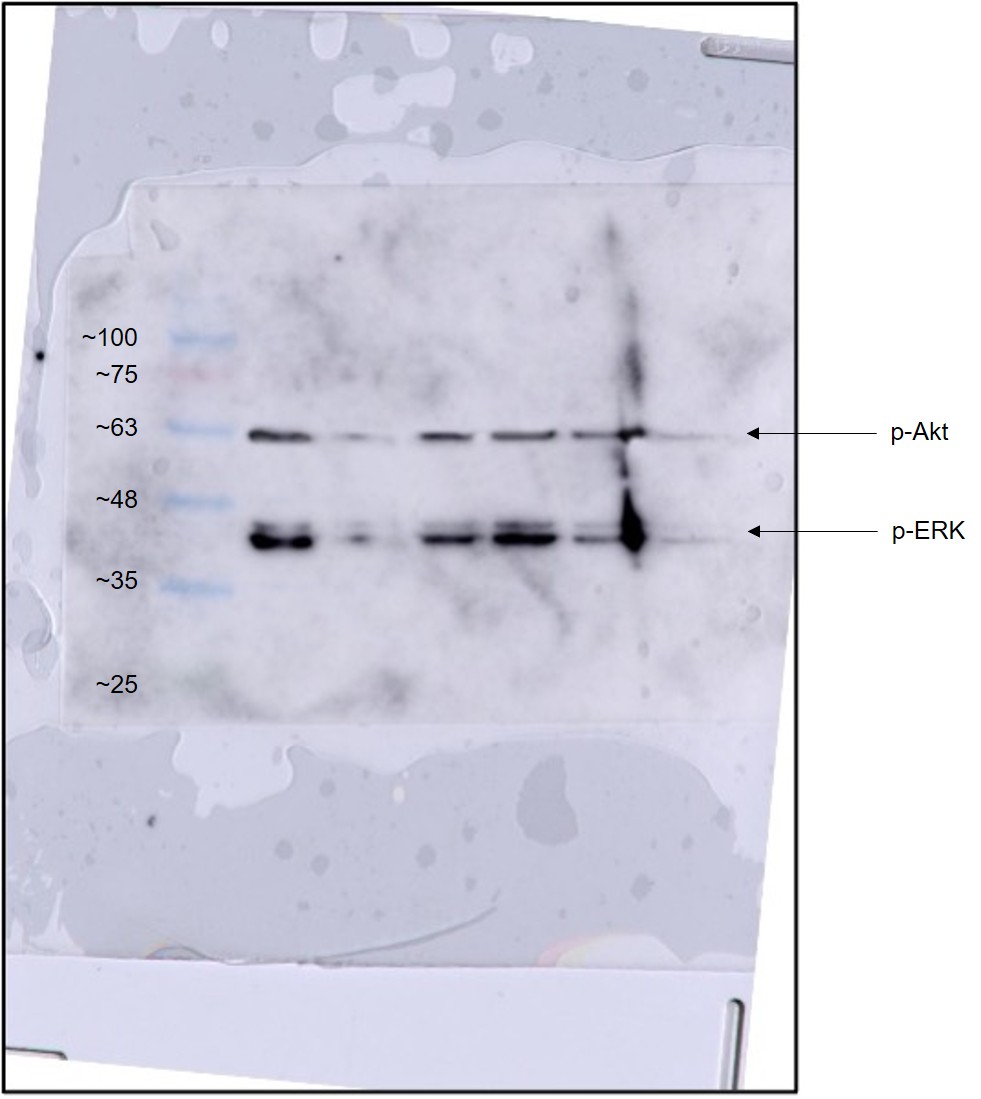

Supplement: Supplementary file 1 [file biomolecules-15-01410-s001.zip › Figure 7A_p-Akt_Batch 2.jpg]

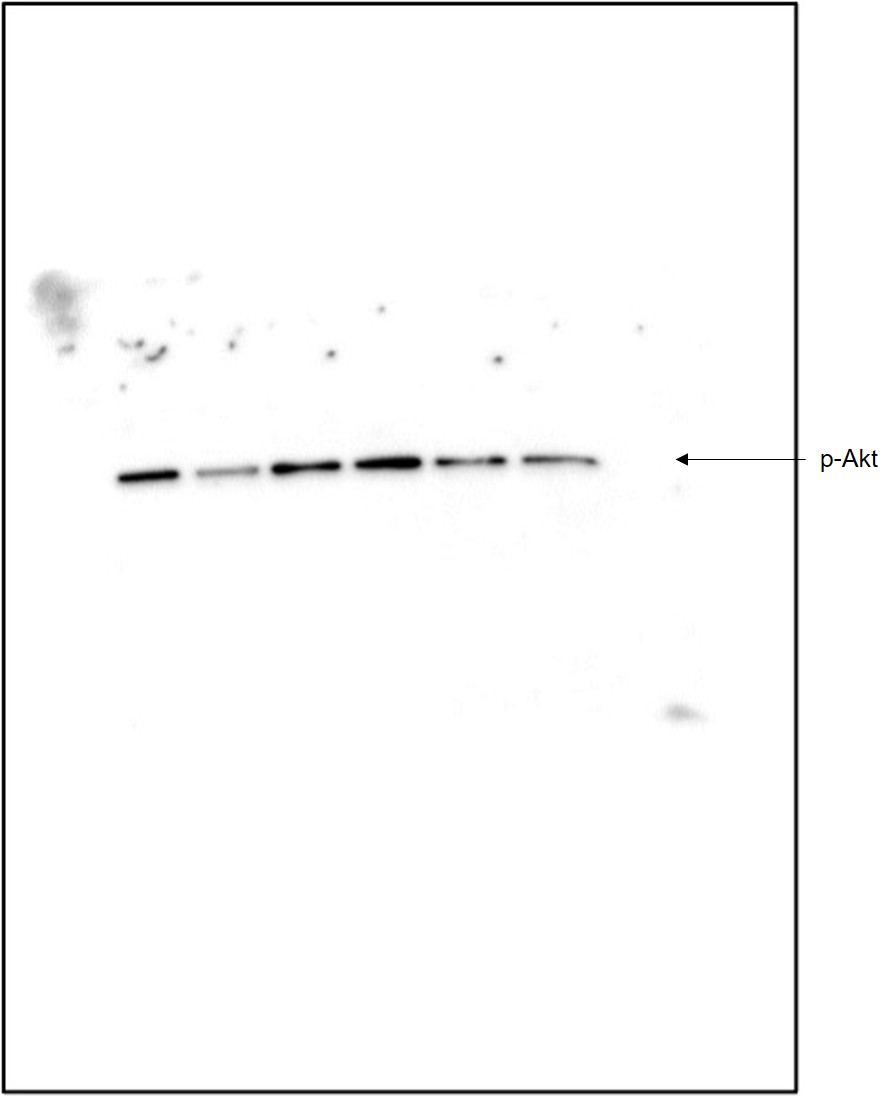

Supplement: Supplementary file 1 [file biomolecules-15-01410-s001.zip › Figure 7A_p-Akt_Batch 3.jpg]

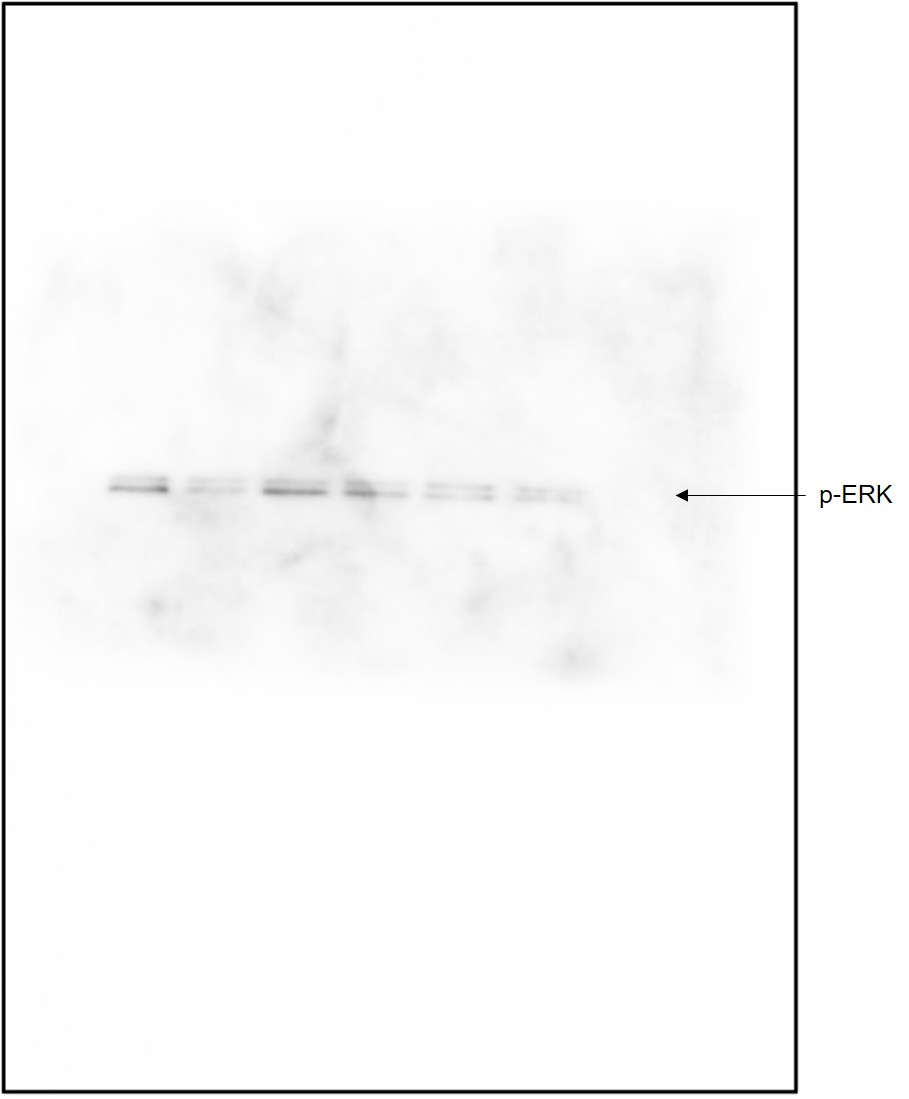

Supplement: Supplementary file 1 [file biomolecules-15-01410-s001.zip › Figure 7A_p-ERK_Batch 3.jpg]

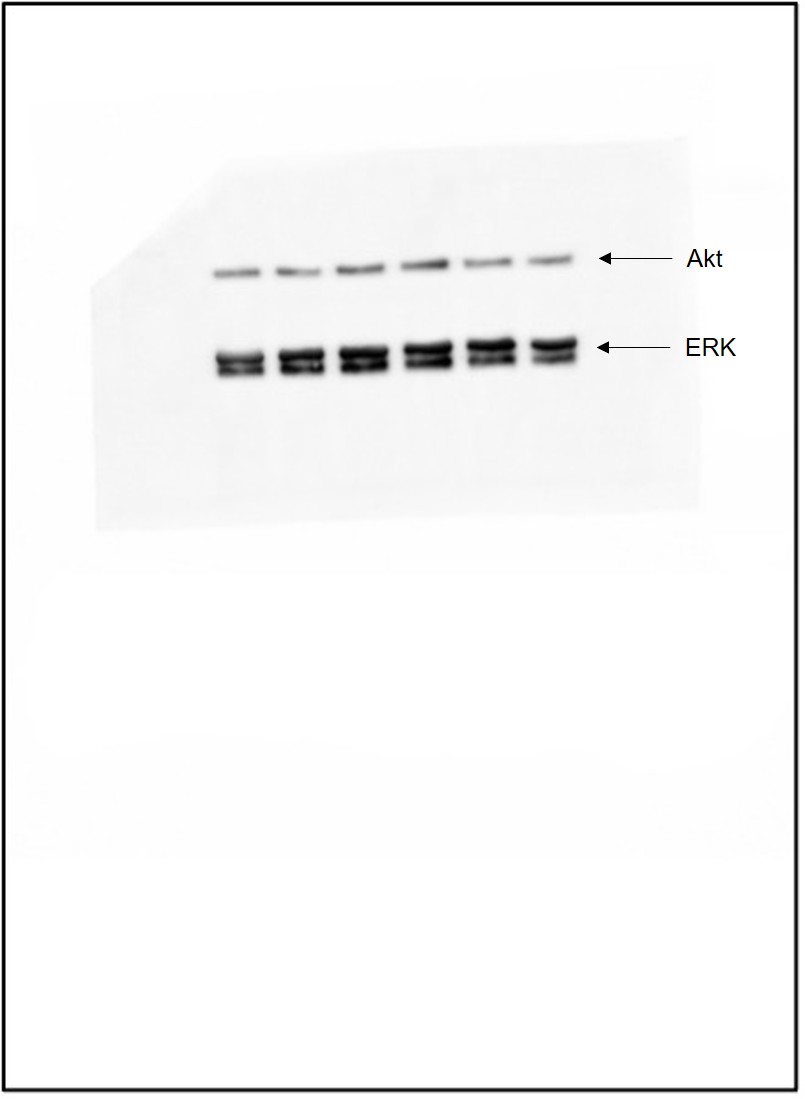

Supplement: Supplementary file 1 [file biomolecules-15-01410-s001.zip › Figure 7B_Akt_Batch 1.jpg]

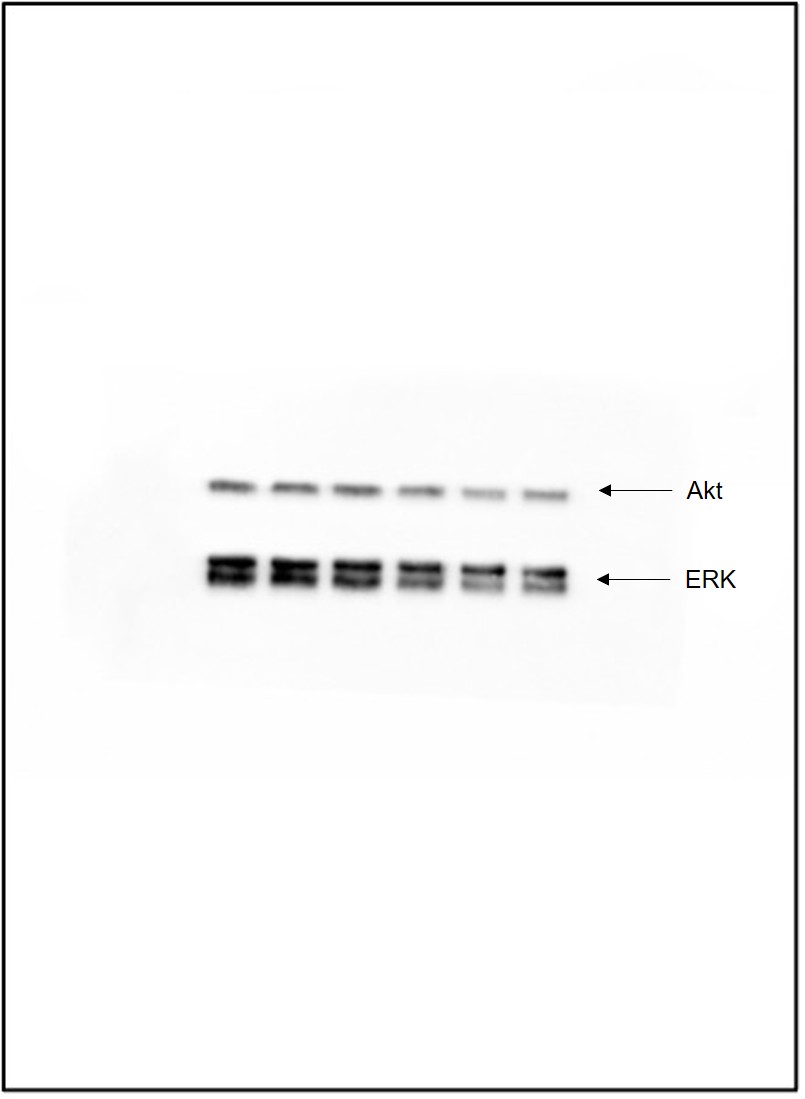

Supplement: Supplementary file 1 [file biomolecules-15-01410-s001.zip › Figure 7B_Akt_Batch 2.jpg]

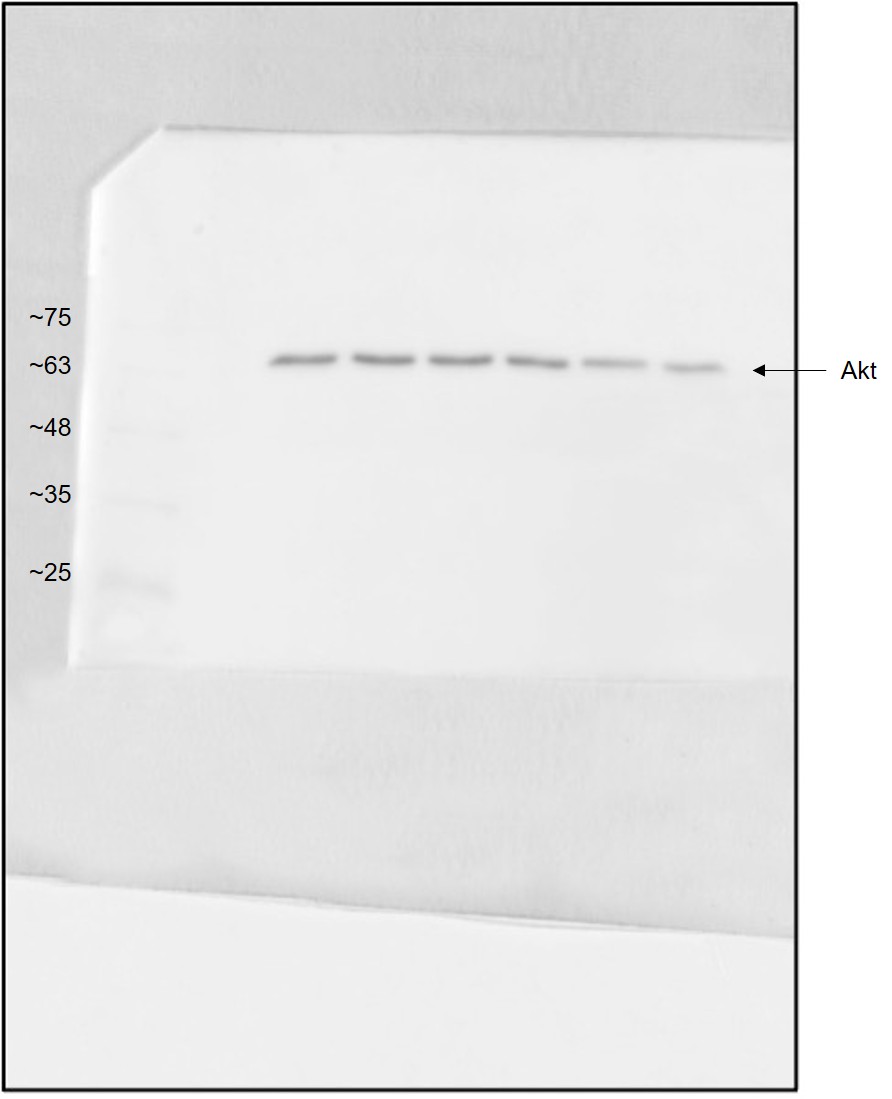

Supplement: Supplementary file 1 [file biomolecules-15-01410-s001.zip › Figure 7B_Akt_Batch 3.jpg]

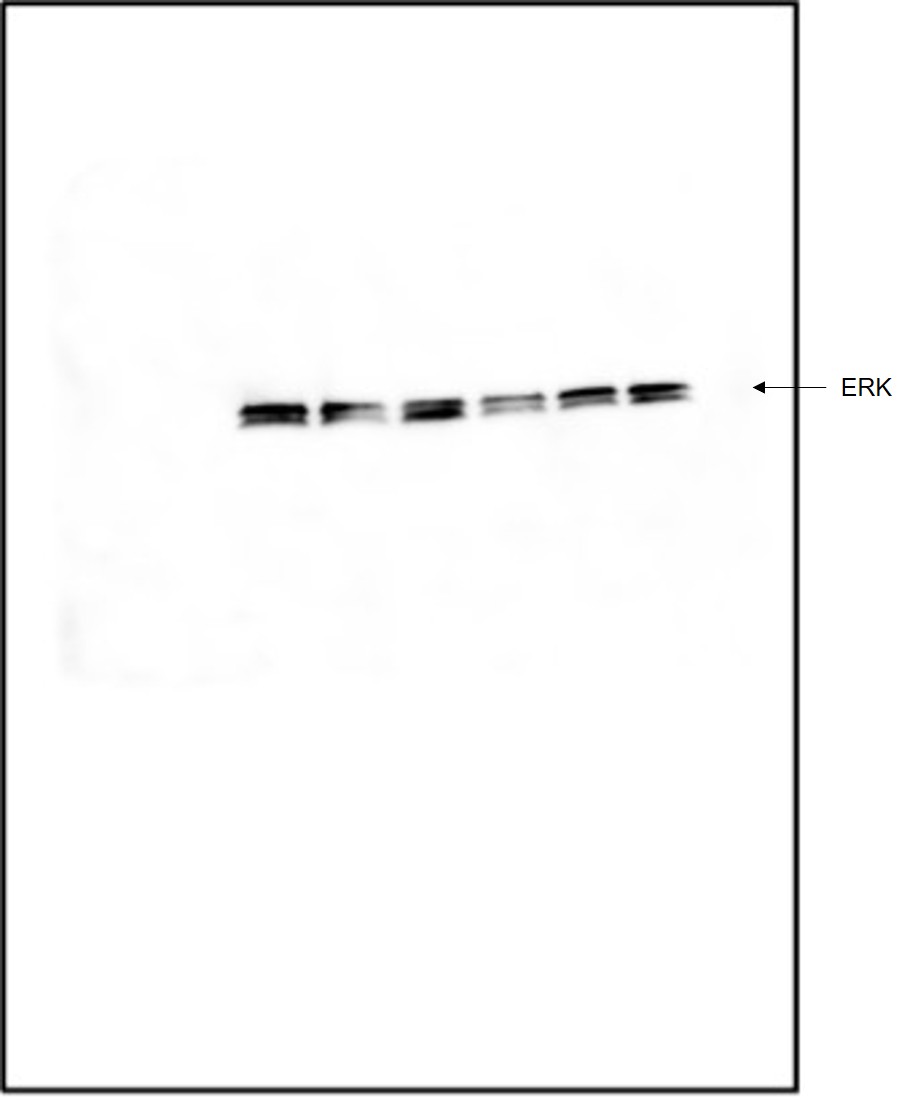

Supplement: Supplementary file 1 [file biomolecules-15-01410-s001.zip › Figure 7B_ERK_Batch 3.jpg]

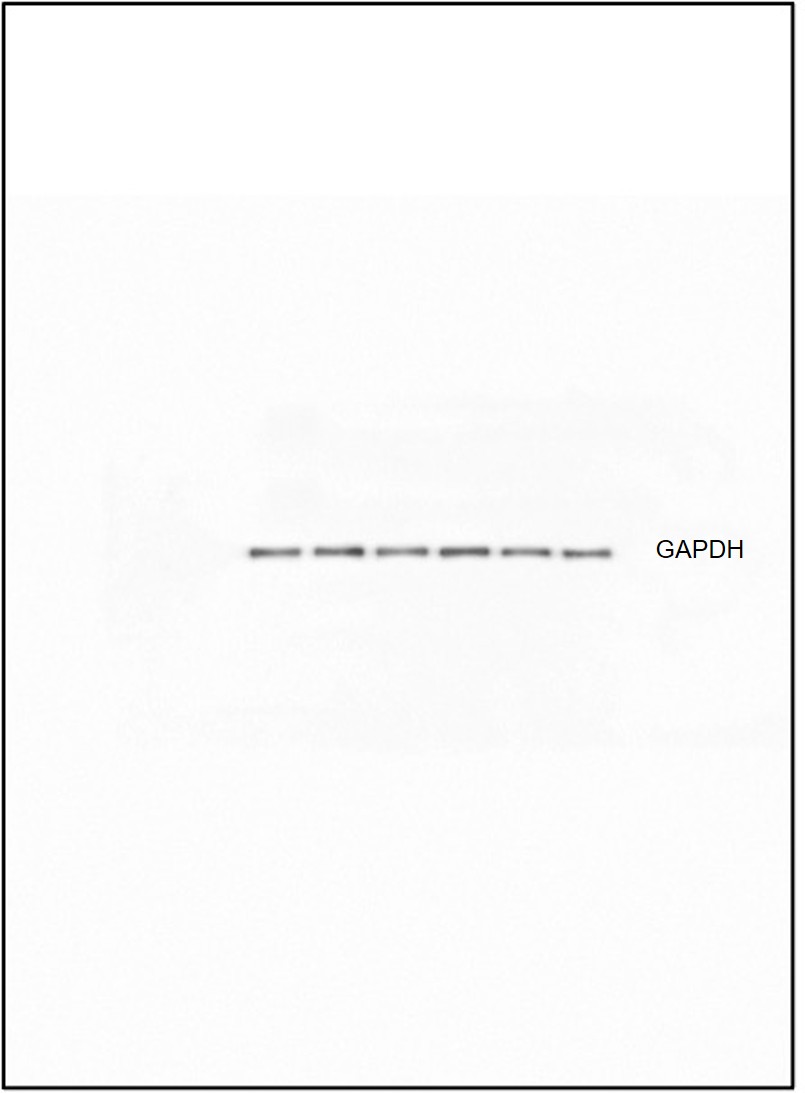

Supplement: Supplementary file 1 [file biomolecules-15-01410-s001.zip › Figure 7B_GAPDH_Batch 1.jpg]

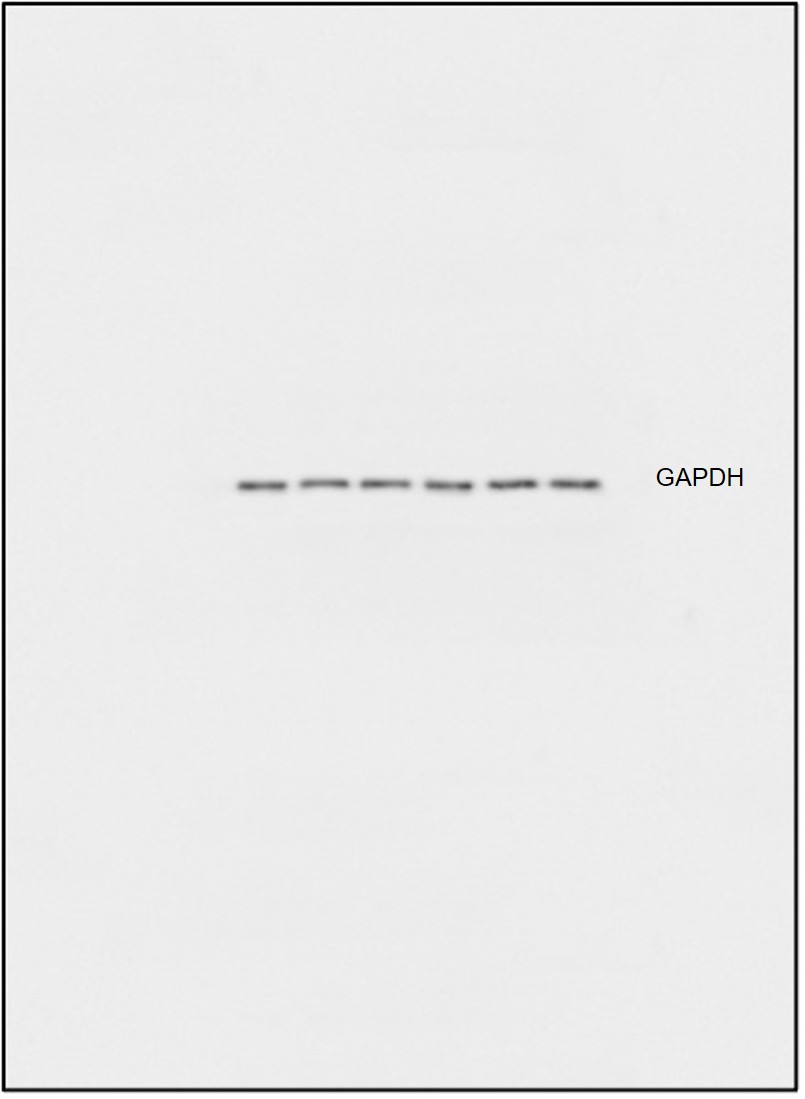

Supplement: Supplementary file 1 [file biomolecules-15-01410-s001.zip › Figure 7B_GAPDH_Batch 2.jpg]

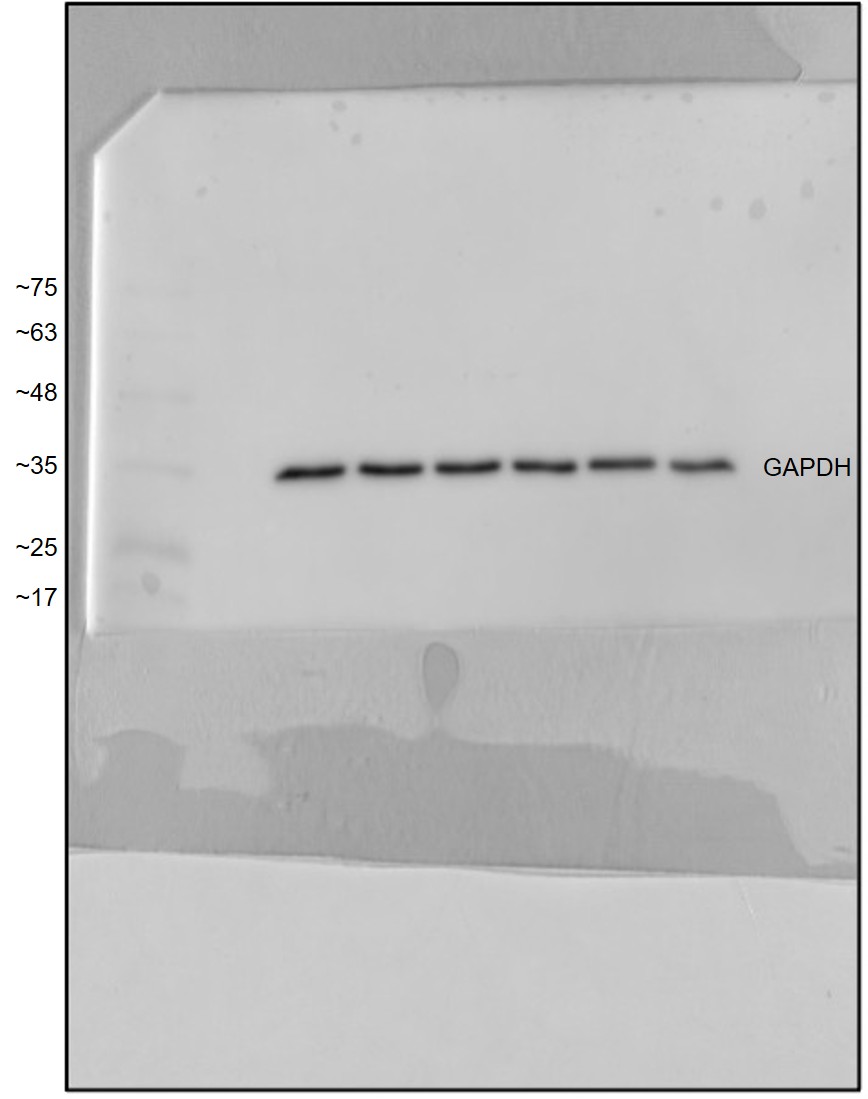

Supplement: Supplementary file 1 [file biomolecules-15-01410-s001.zip › Figure 7B_GAPDH_Batch 3.jpg]

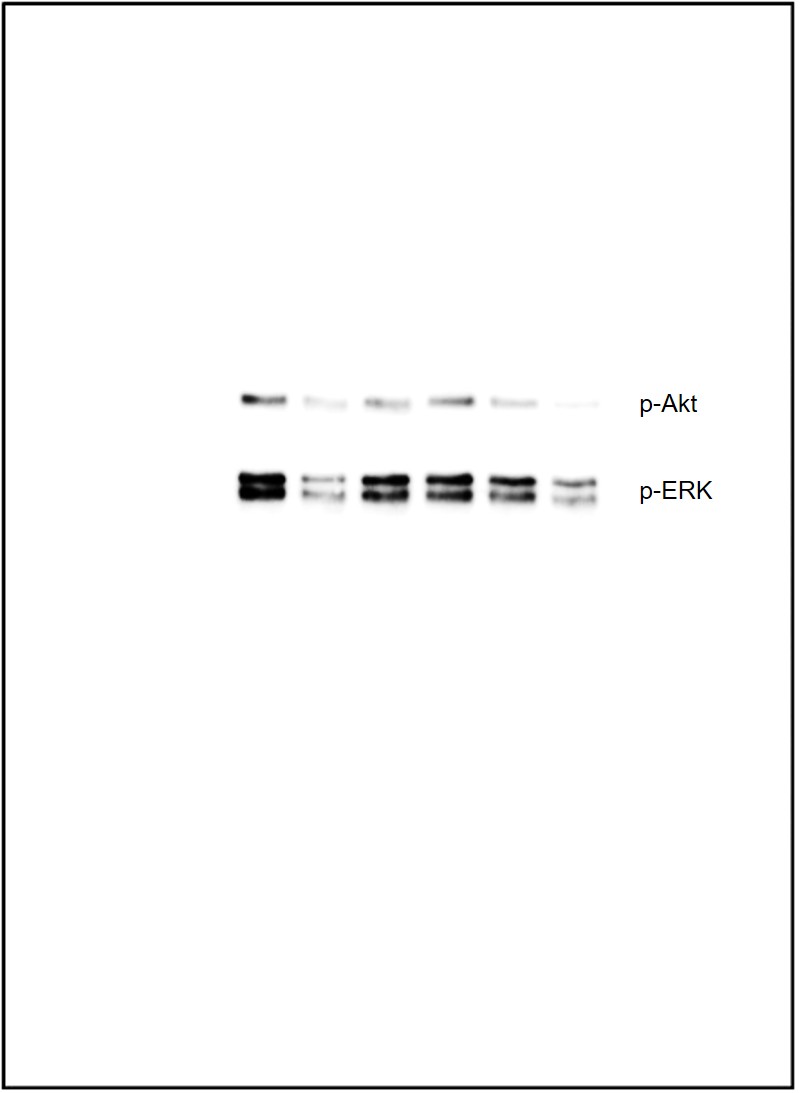

Supplement: Supplementary file 1 [file biomolecules-15-01410-s001.zip › Figure 7B_p-Akt_Batch 1.jpg]

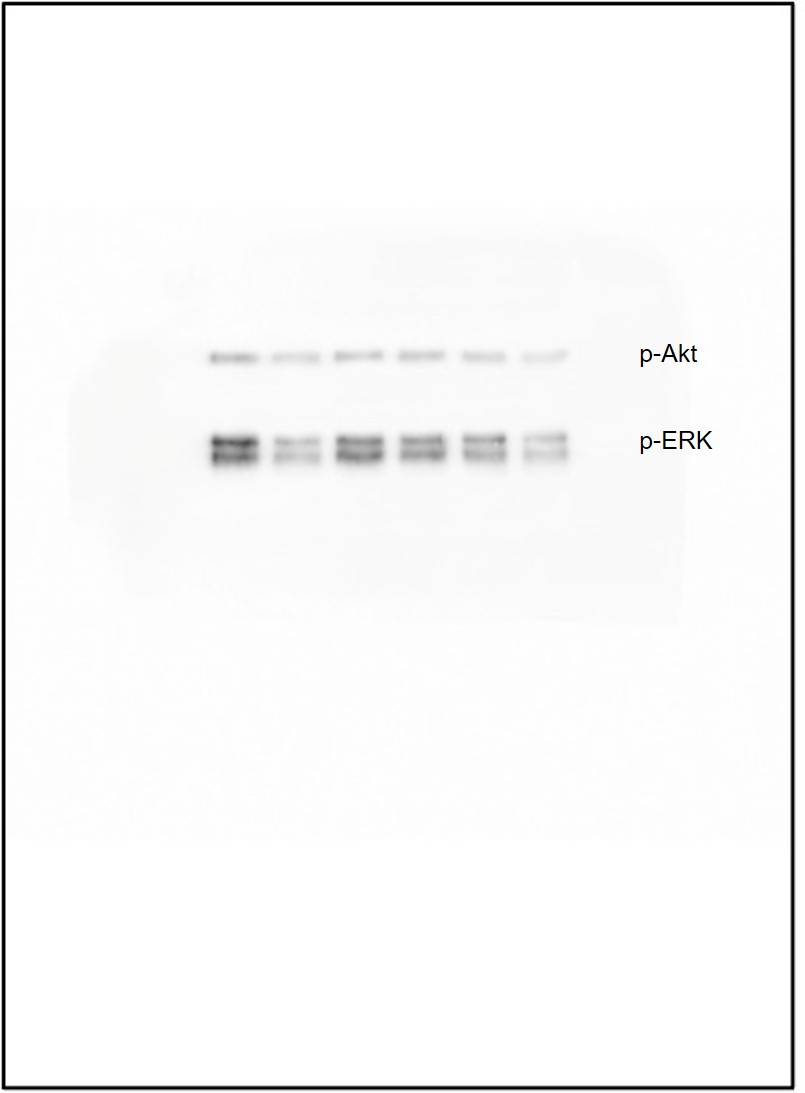

Supplement: Supplementary file 1 [file biomolecules-15-01410-s001.zip › Figure 7B_p-Akt_Batch 2.jpg]

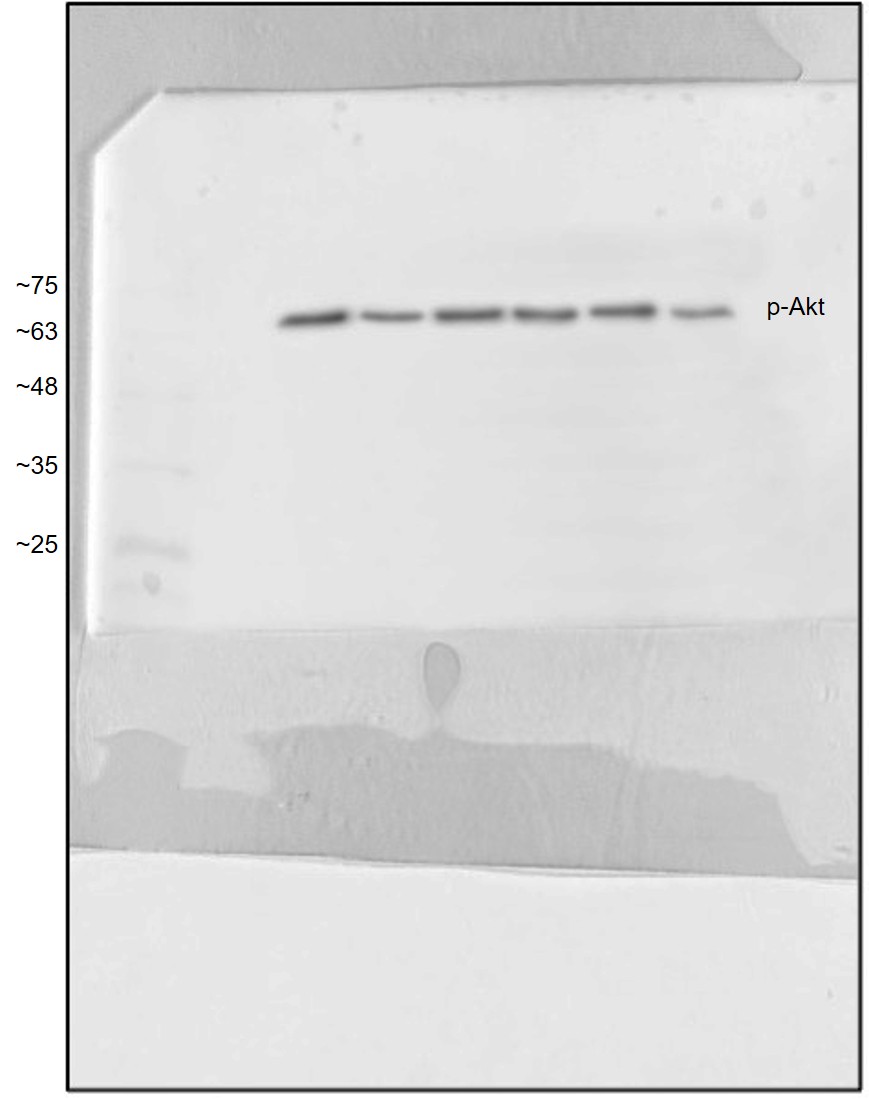

Supplement: Supplementary file 1 [file biomolecules-15-01410-s001.zip › Figure 7B_p-Akt_Batch 3.jpg]

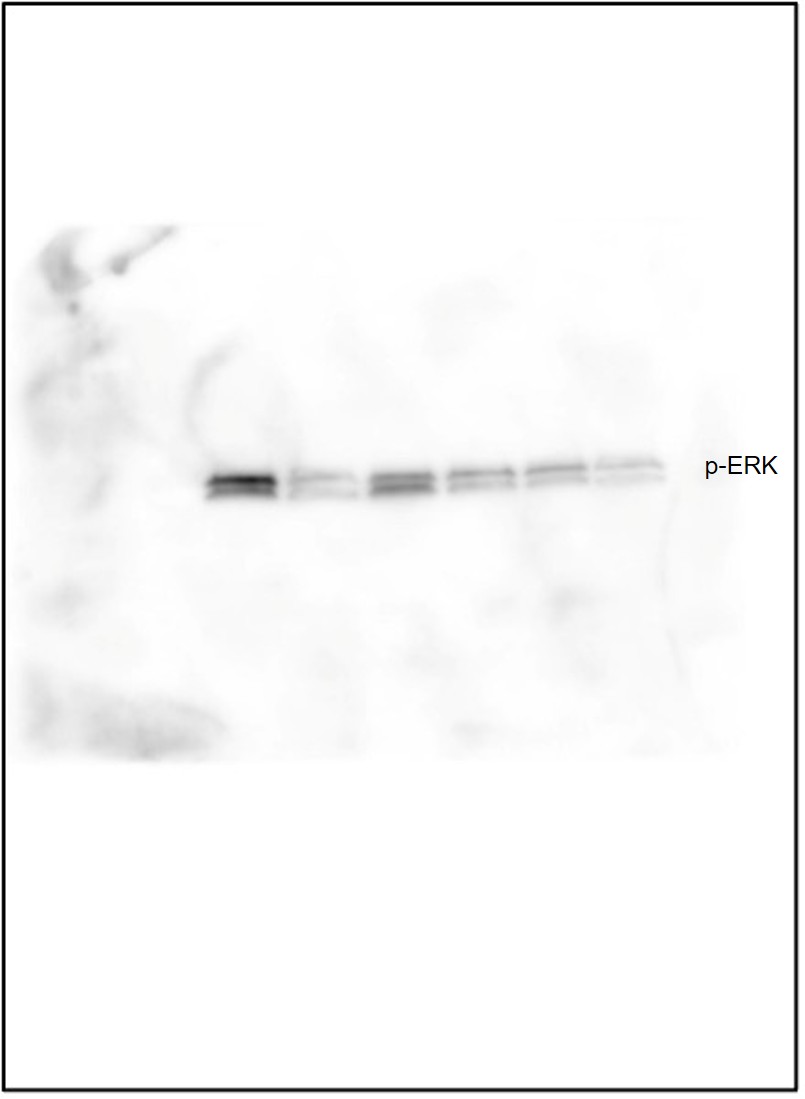

Supplement: Supplementary file 1 [file biomolecules-15-01410-s001.zip › Figure 7B_p-ERK_Batch 3.jpg]

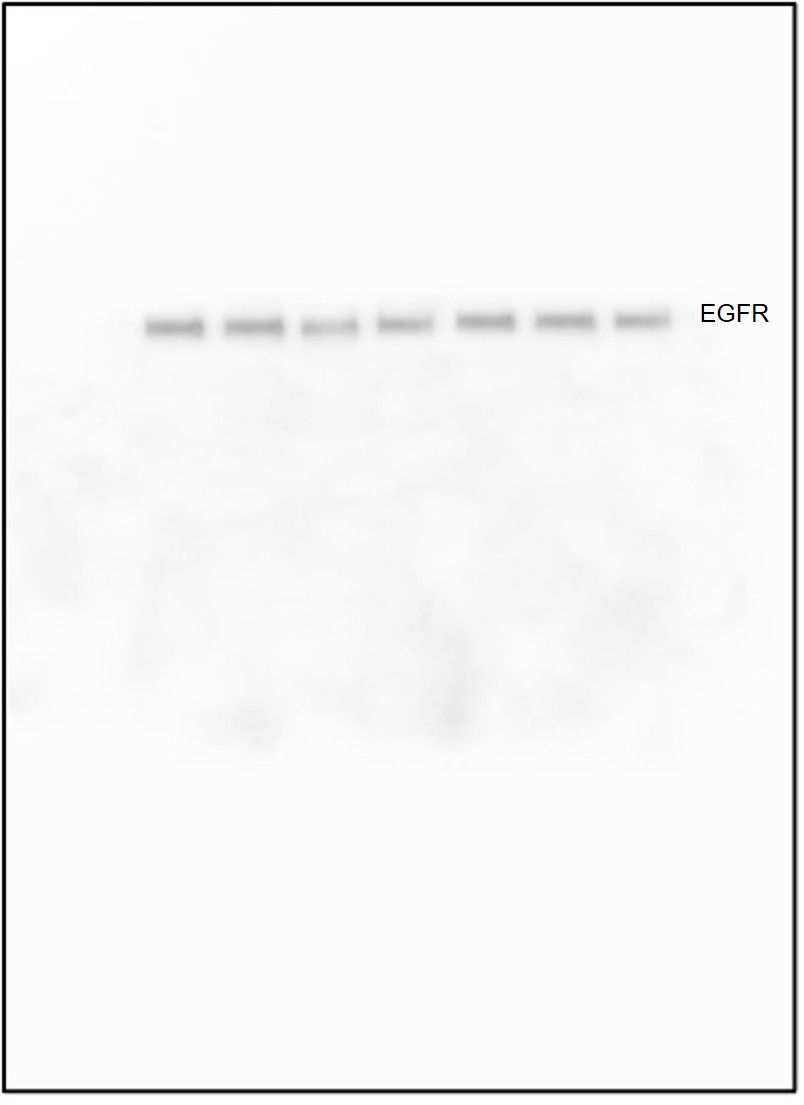

Supplement: Supplementary file 1 [file biomolecules-15-01410-s001.zip › Figure 8A_EGFR_Batch 1.jpg]

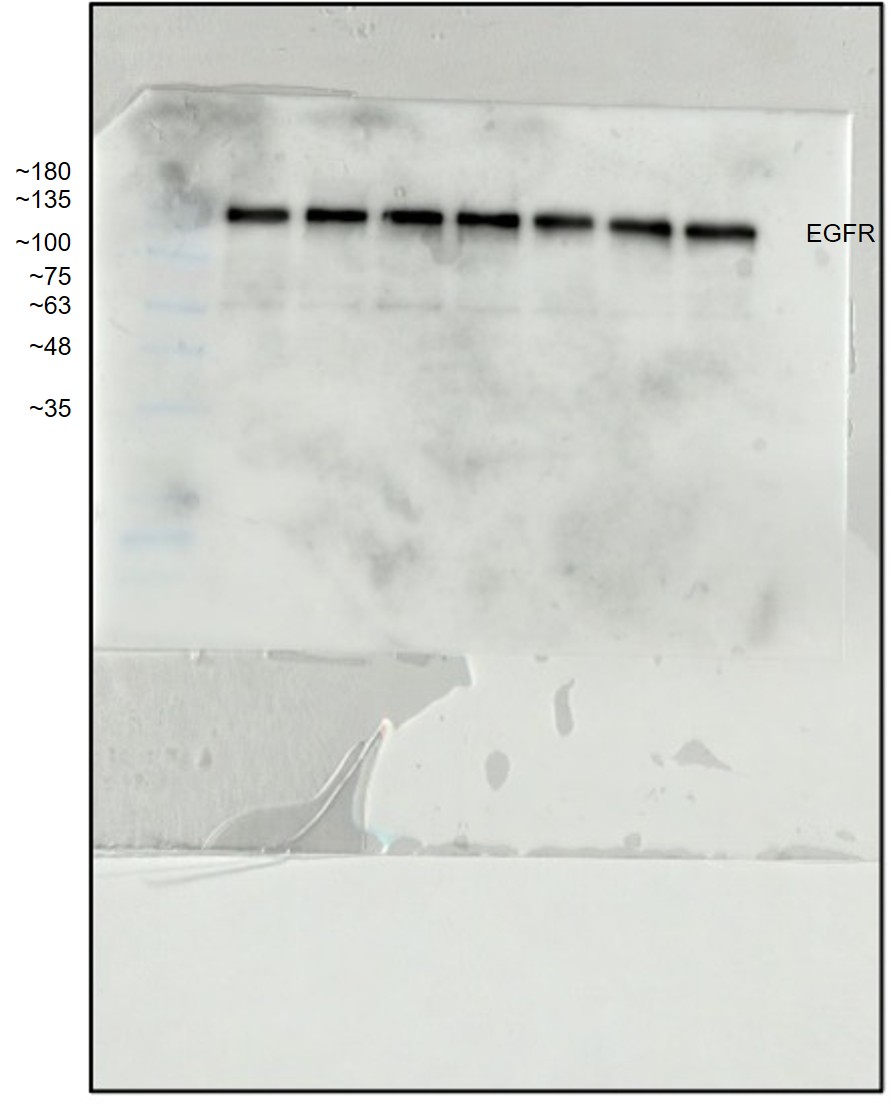

Supplement: Supplementary file 1 [file biomolecules-15-01410-s001.zip › Figure 8A_EGFR_Batch 2.jpg]

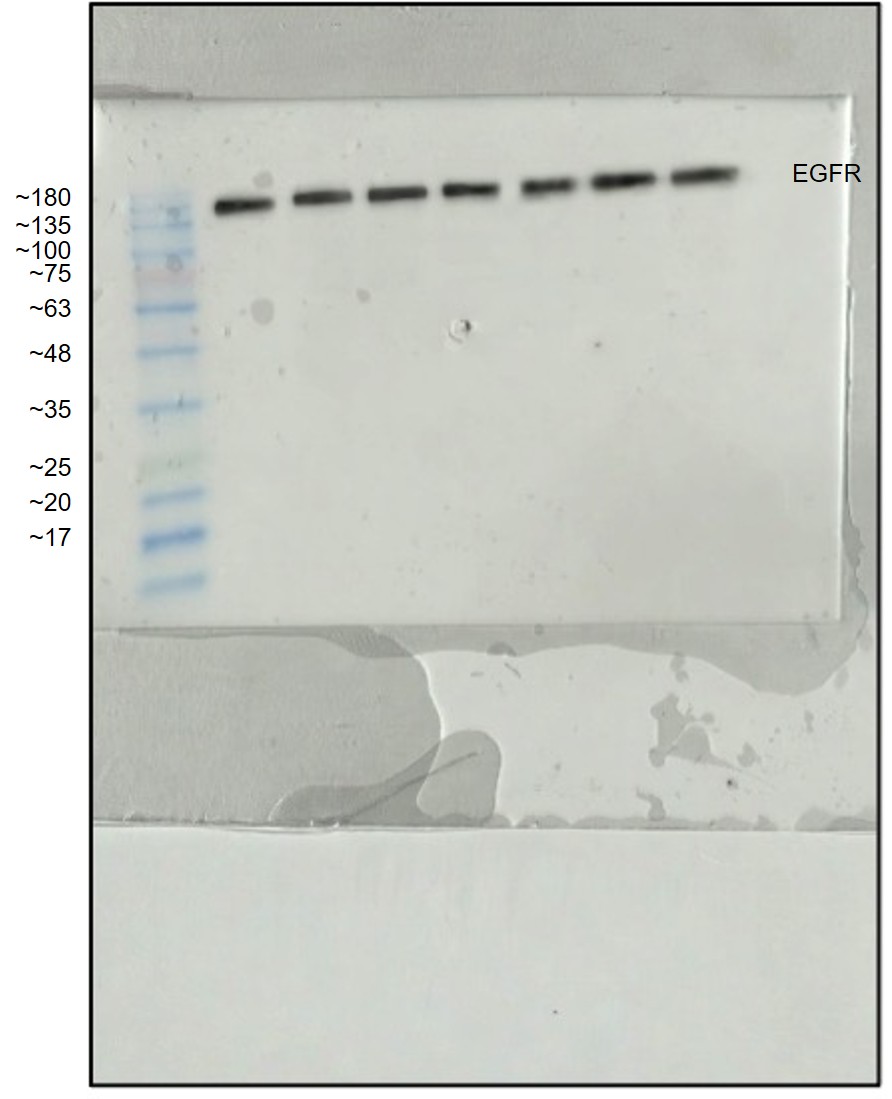

Supplement: Supplementary file 1 [file biomolecules-15-01410-s001.zip › Figure 8A_EGFR_Batch 3.jpg]

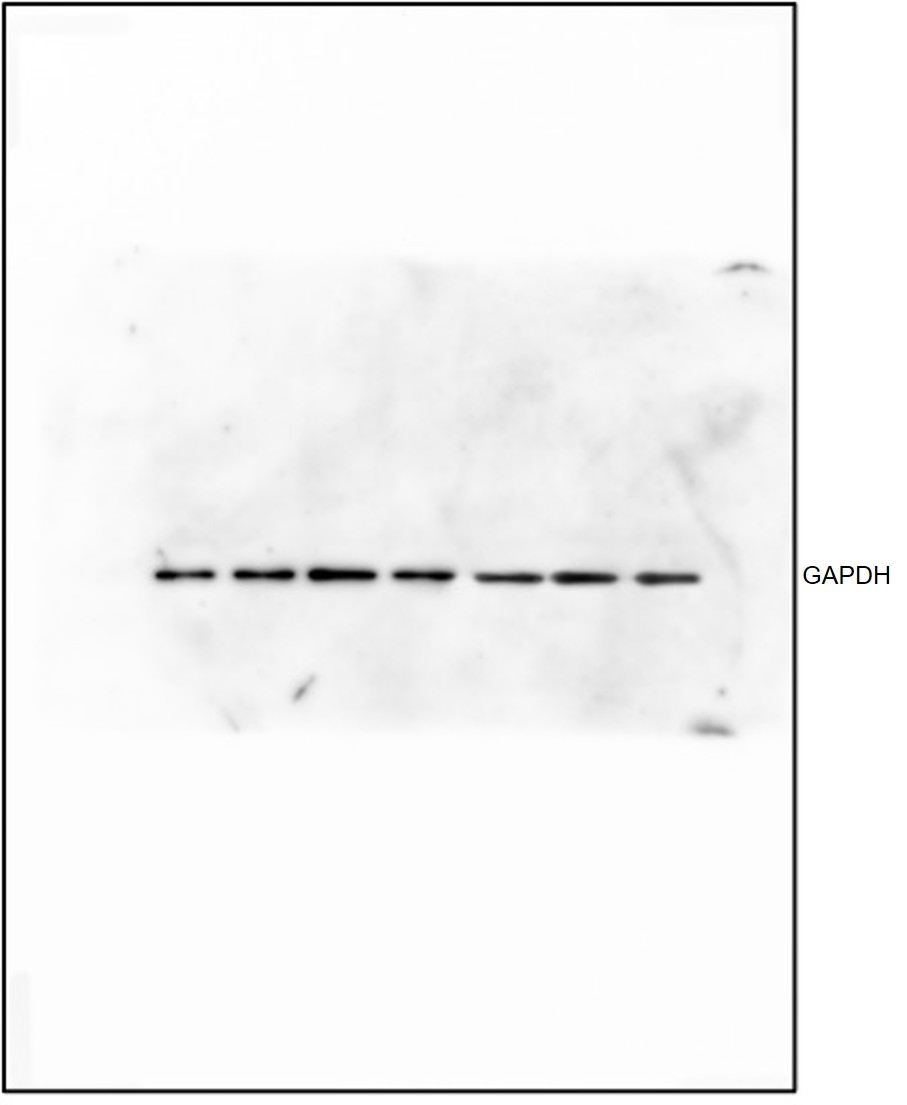

Supplement: Supplementary file 1 [file biomolecules-15-01410-s001.zip › Figure 8A_GAPDH_Batch 1.jpg]

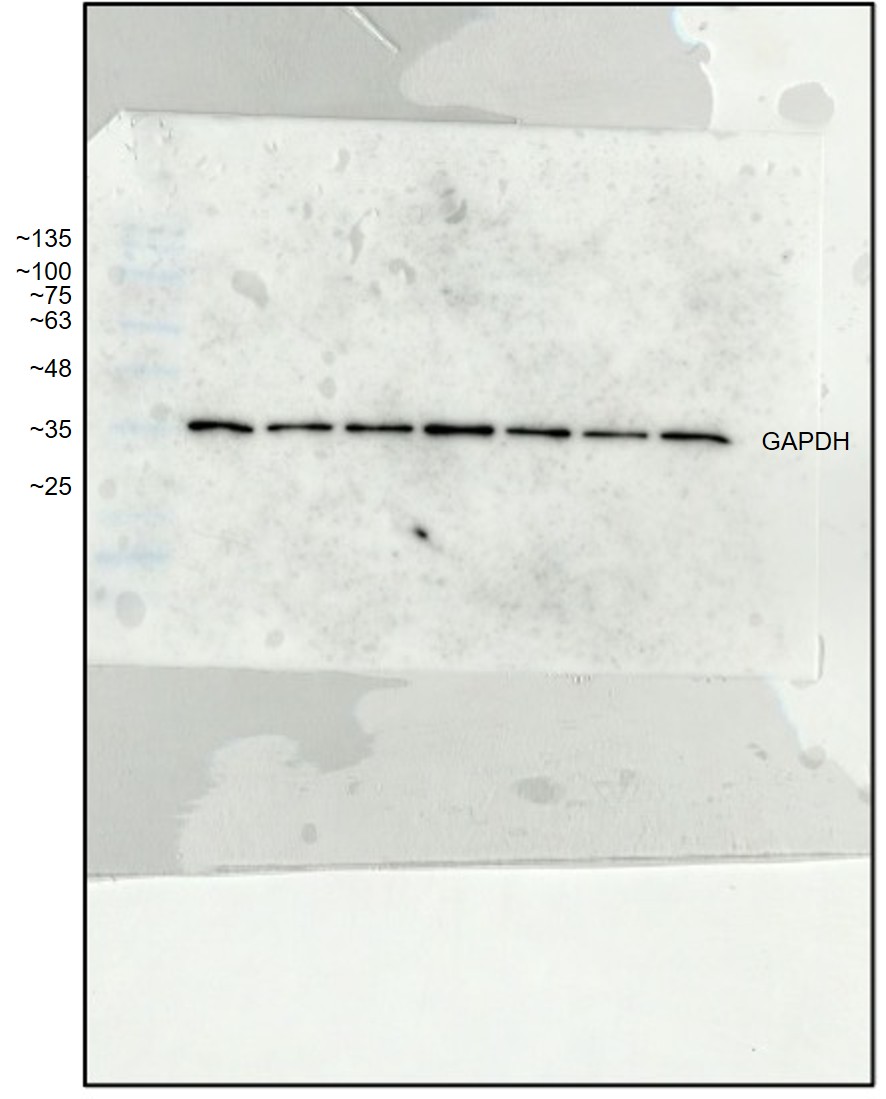

Supplement: Supplementary file 1 [file biomolecules-15-01410-s001.zip › Figure 8A_GAPDH_Batch 2.jpg]

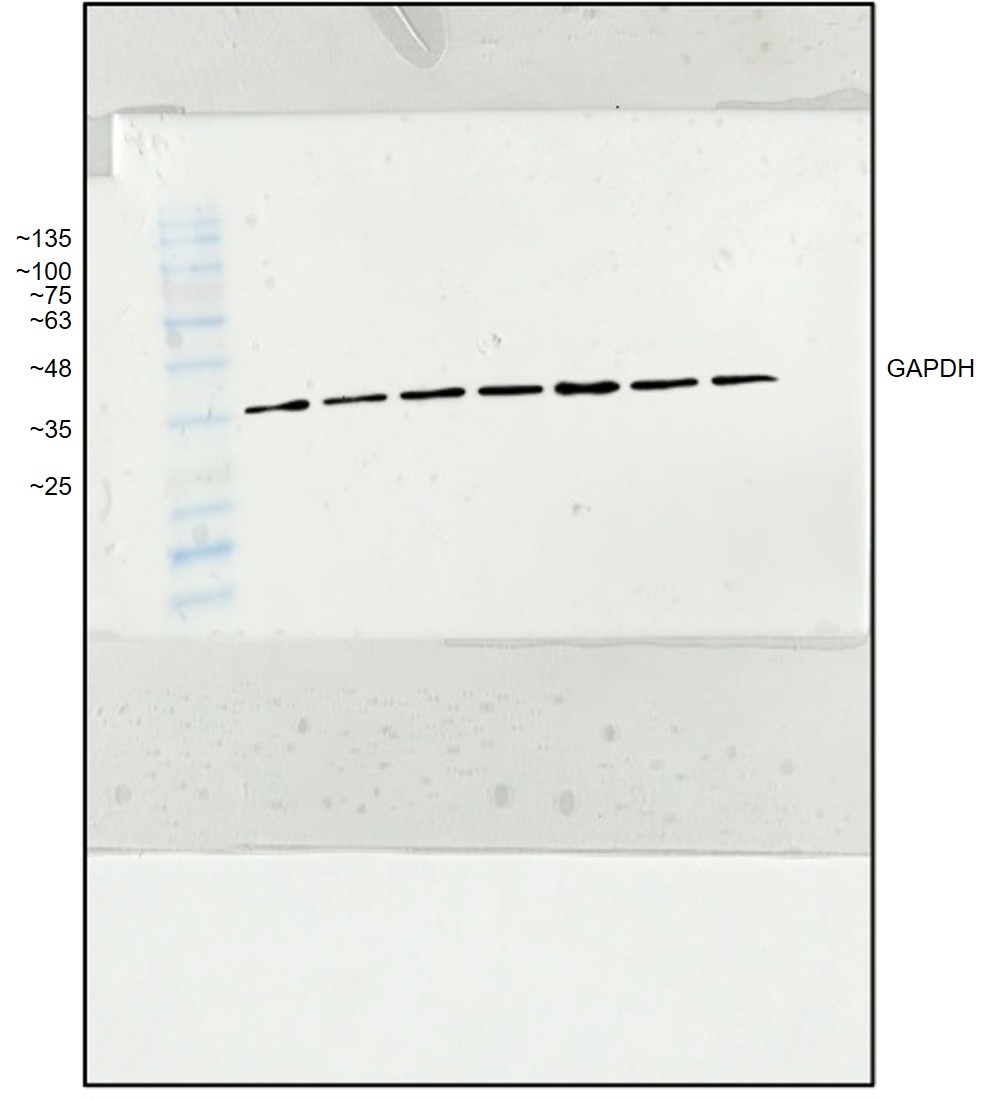

Supplement: Supplementary file 1 [file biomolecules-15-01410-s001.zip › Figure 8A_GAPDH_Batch 3.jpg]

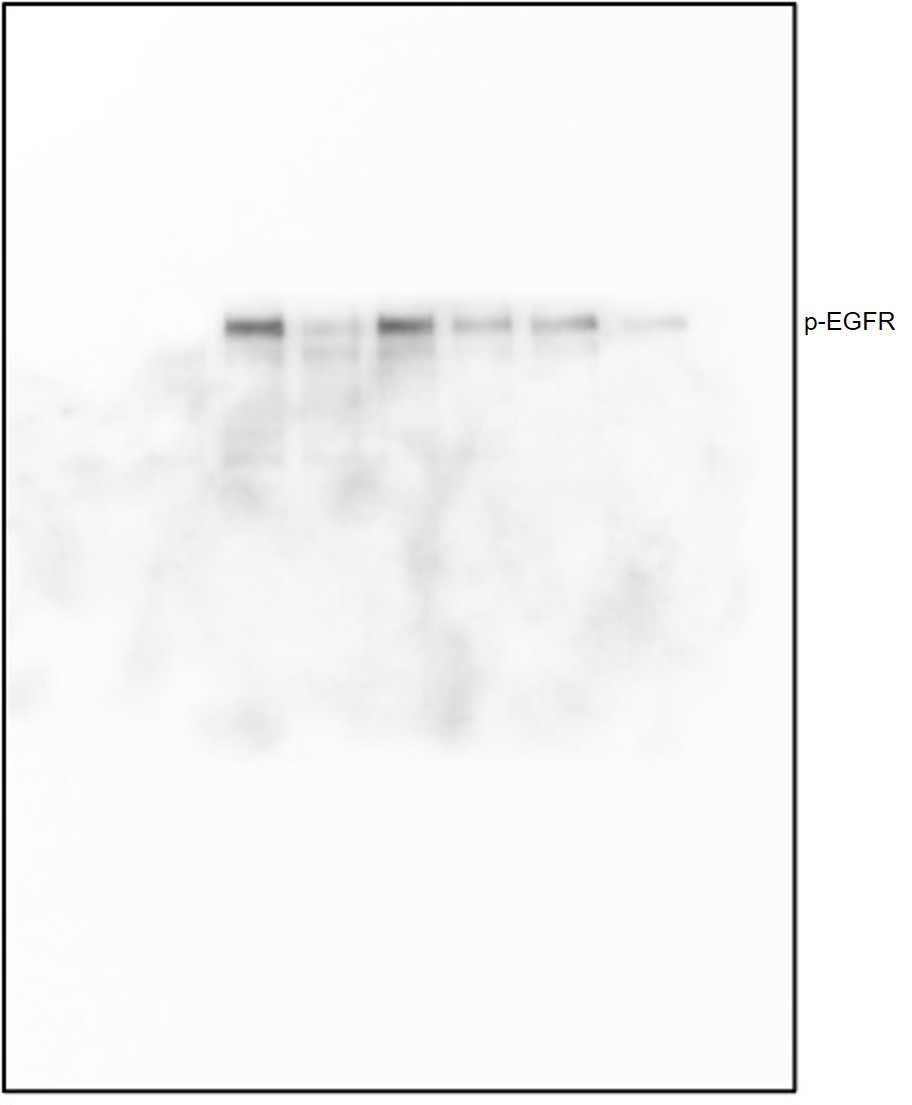

Supplement: Supplementary file 1 [file biomolecules-15-01410-s001.zip › Figure 8A_p-EGFR_Batch 1.jpg]

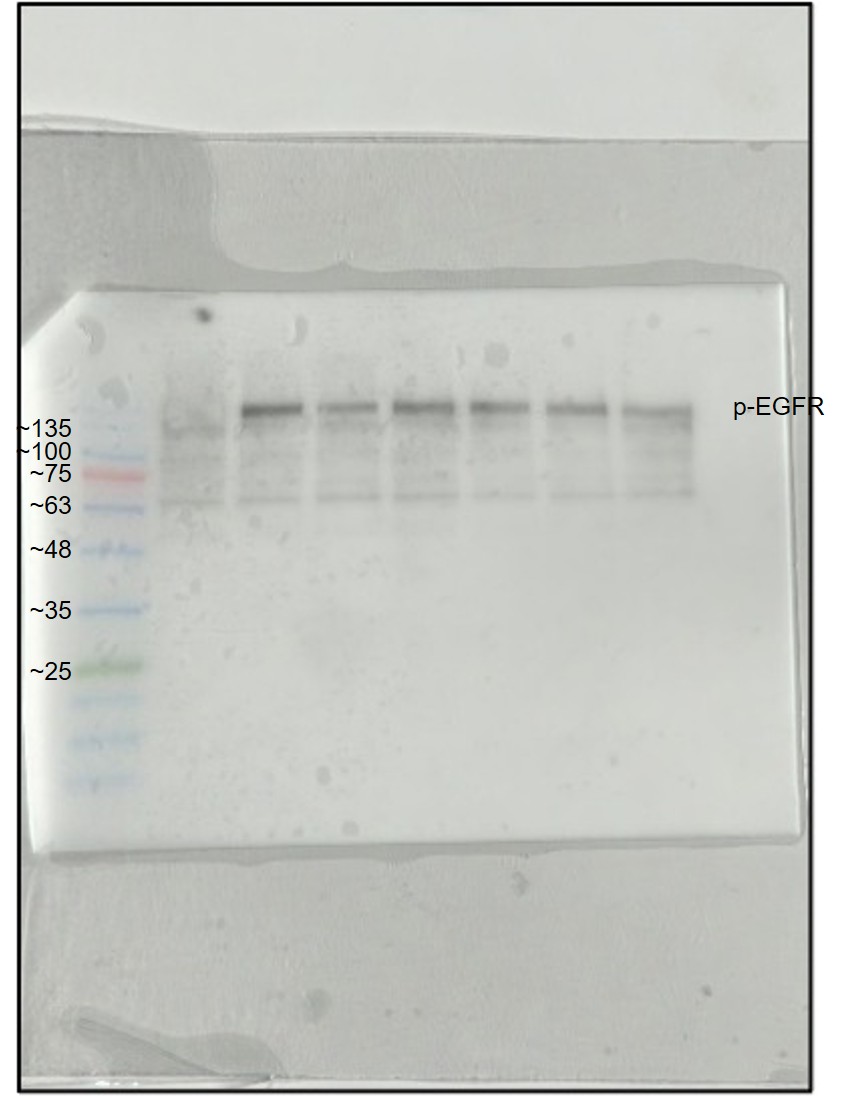

Supplement: Supplementary file 1 [file biomolecules-15-01410-s001.zip › Figure 8A_p-EGFR_Batch 2.jpg]

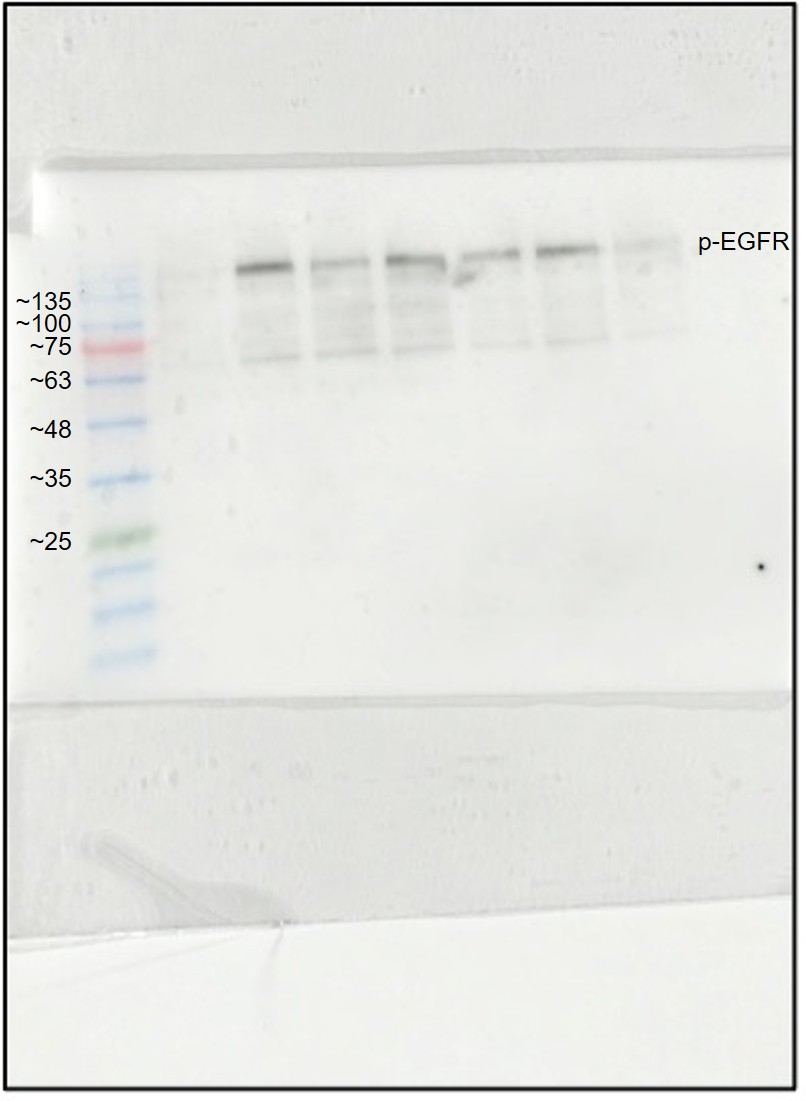

Supplement: Supplementary file 1 [file biomolecules-15-01410-s001.zip › Figure 8A_p-EGFR_Batch 3.jpg]

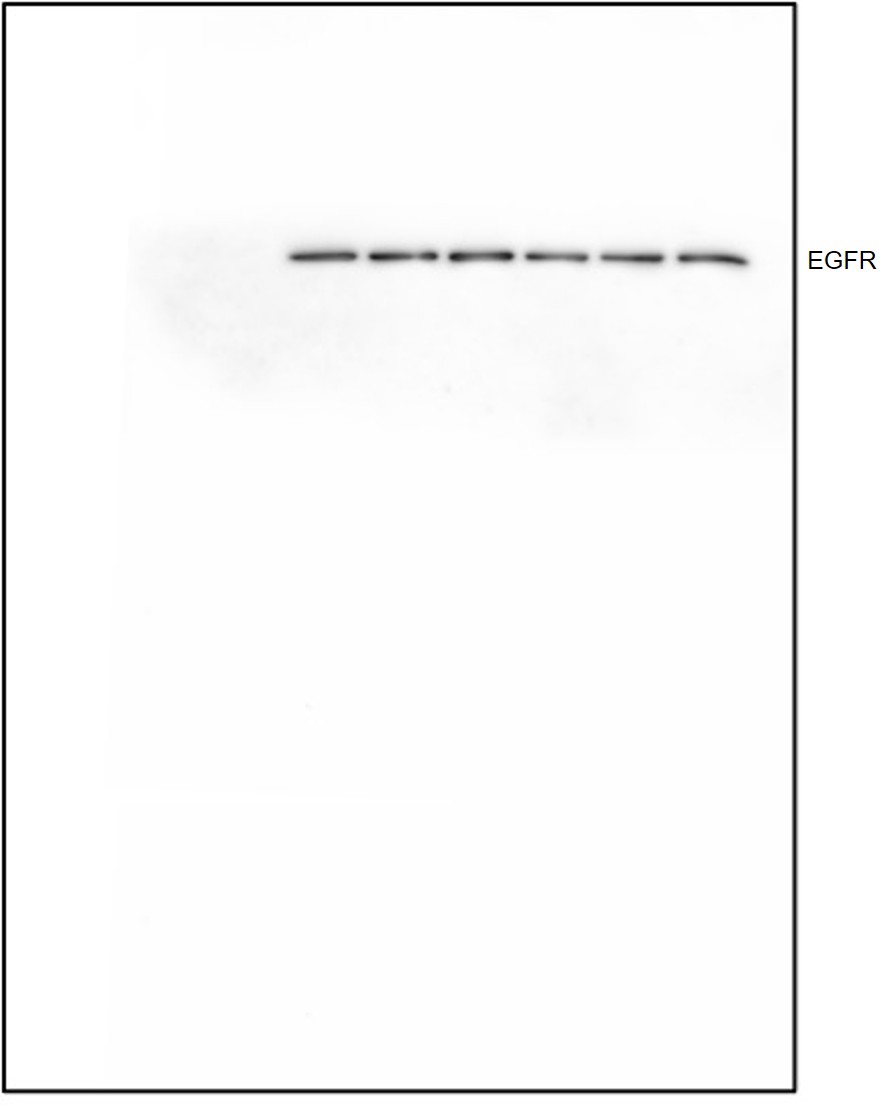

Supplement: Supplementary file 1 [file biomolecules-15-01410-s001.zip › Figure 8B_EGFR_Batch 1.jpg]

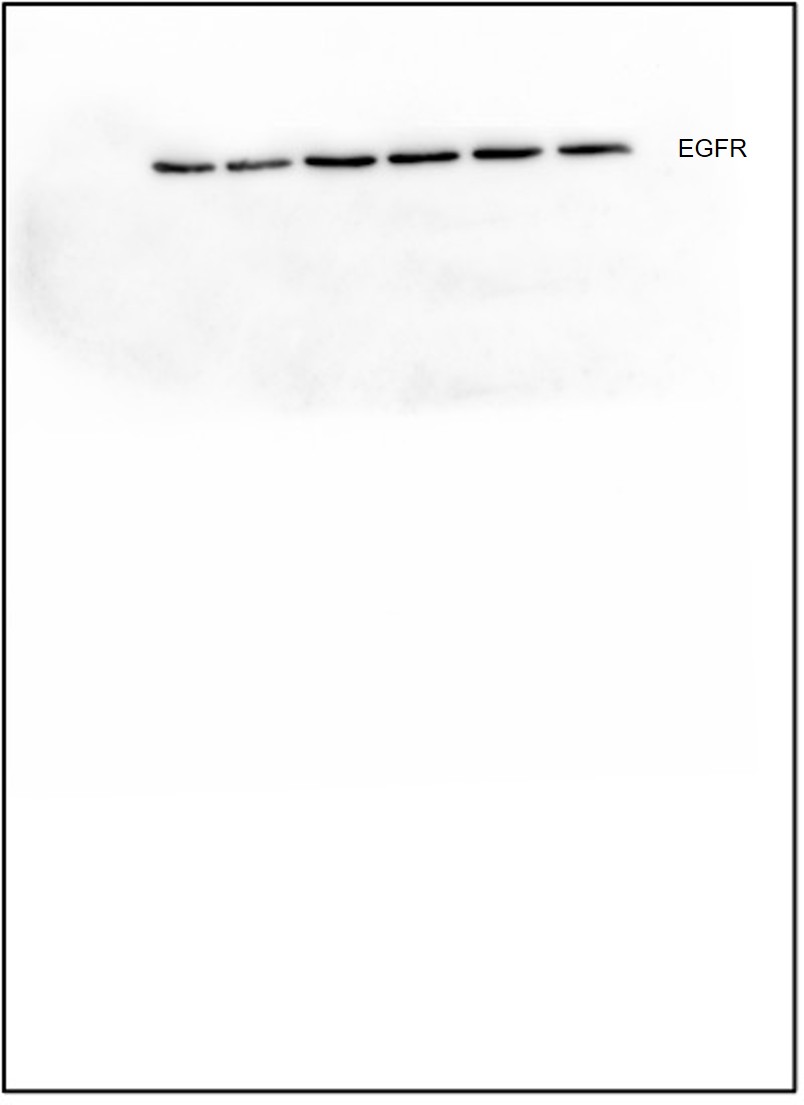

Supplement: Supplementary file 1 [file biomolecules-15-01410-s001.zip › Figure 8B_EGFR_Batch 2.jpg]

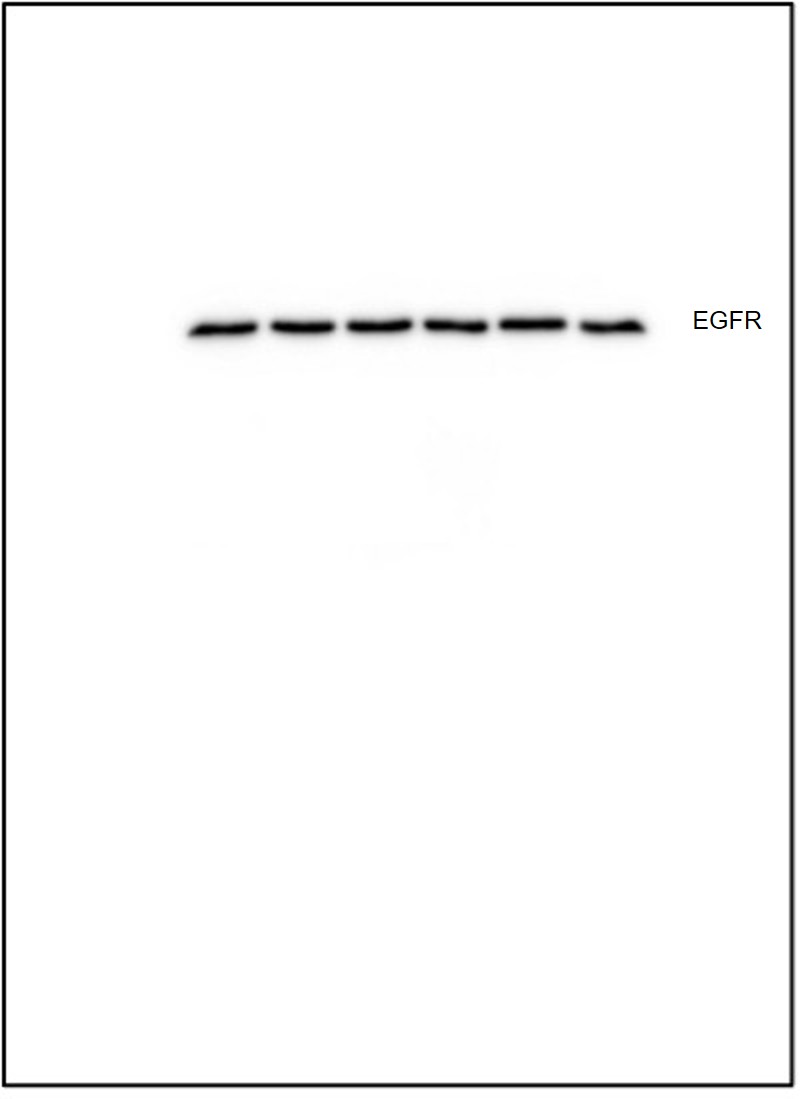

Supplement: Supplementary file 1 [file biomolecules-15-01410-s001.zip › Figure 8B_EGFR_Batch 3.jpg]

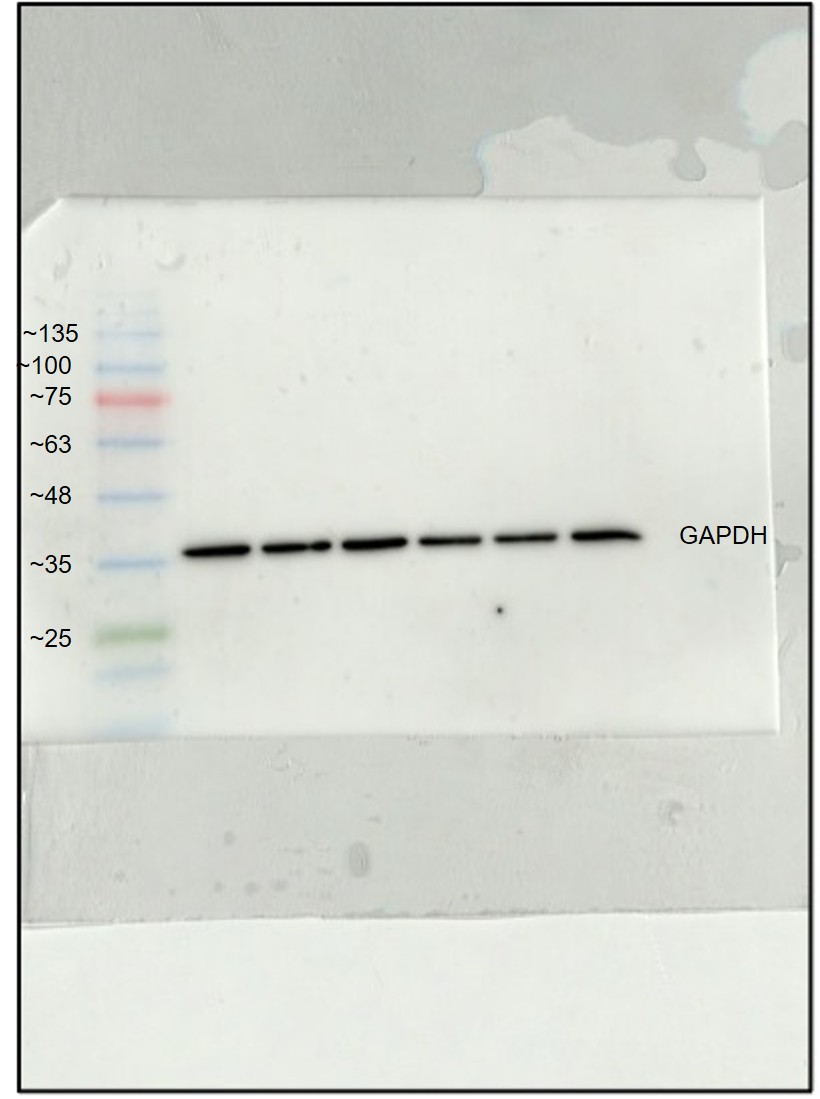

Supplement: Supplementary file 1 [file biomolecules-15-01410-s001.zip › Figure 8B_GAPDH_Batch 1.jpg]

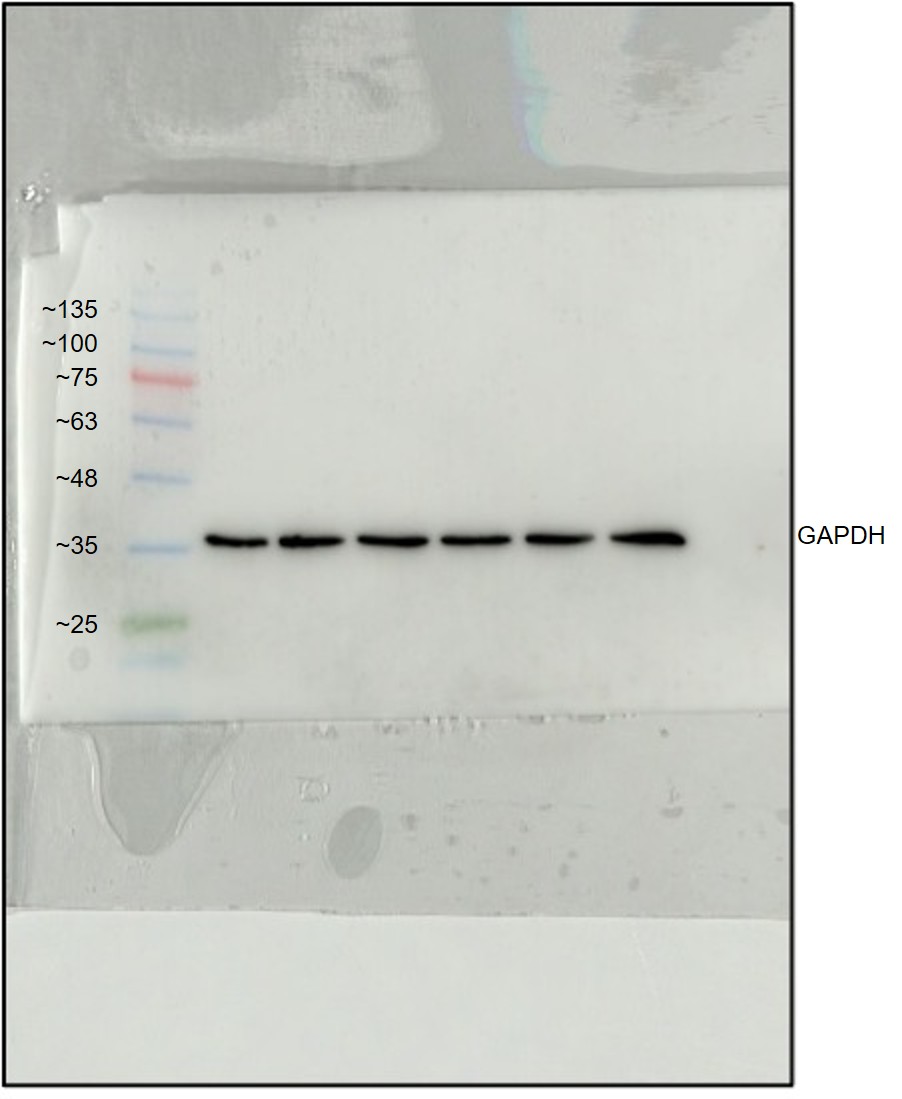

Supplement: Supplementary file 1 [file biomolecules-15-01410-s001.zip › Figure 8B_GAPDH_Batch 2.jpg]

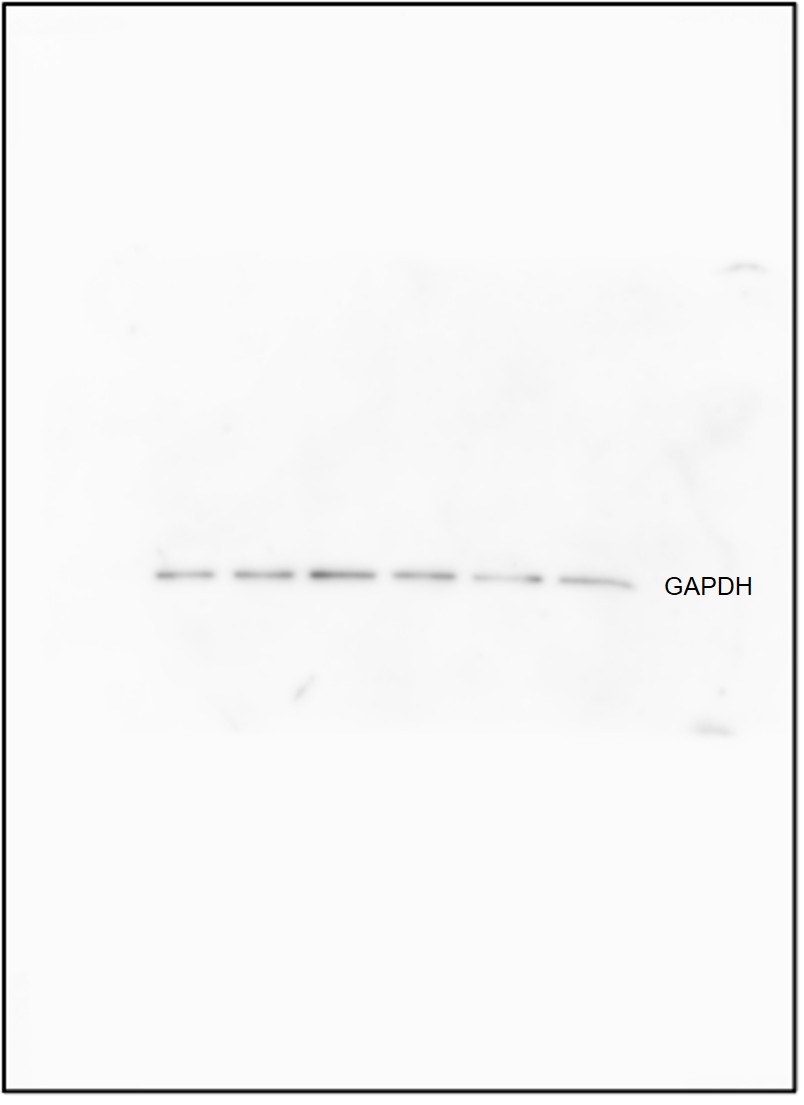

Supplement: Supplementary file 1 [file biomolecules-15-01410-s001.zip › Figure 8B_GAPDH_Batch 3.jpg]

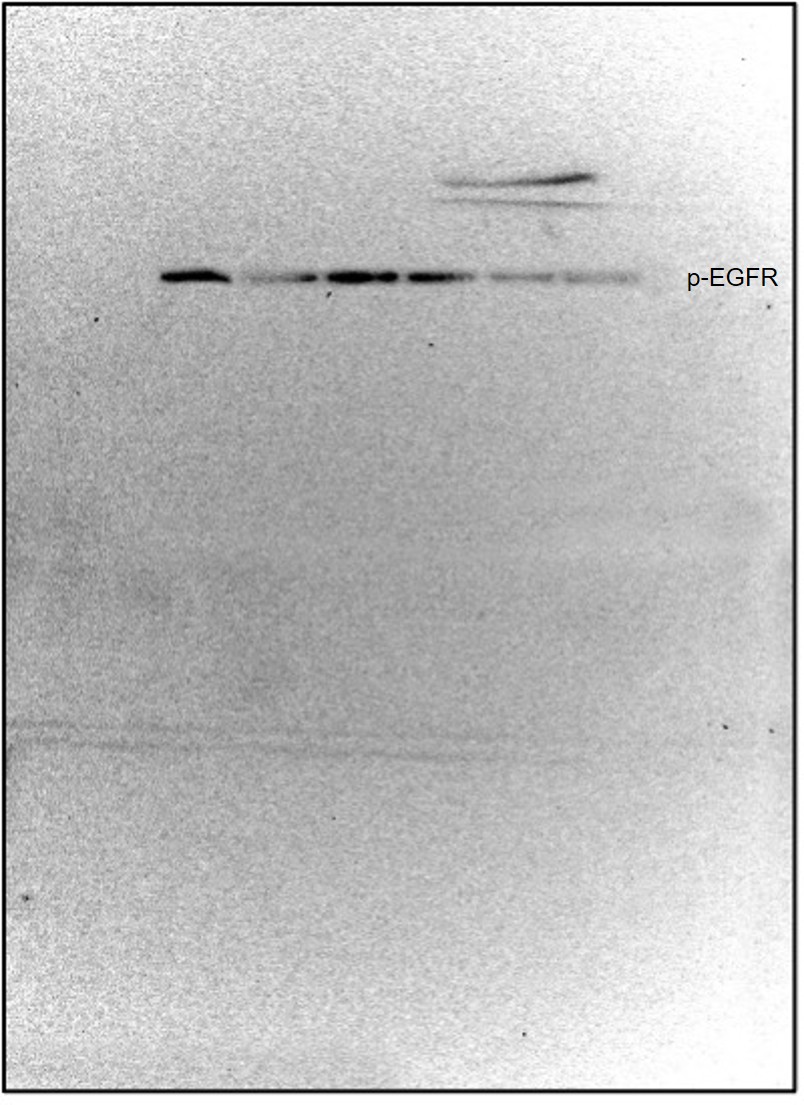

Supplement: Supplementary file 1 [file biomolecules-15-01410-s001.zip › Figure 8B_p-EGFR_Batch 1.jpg]

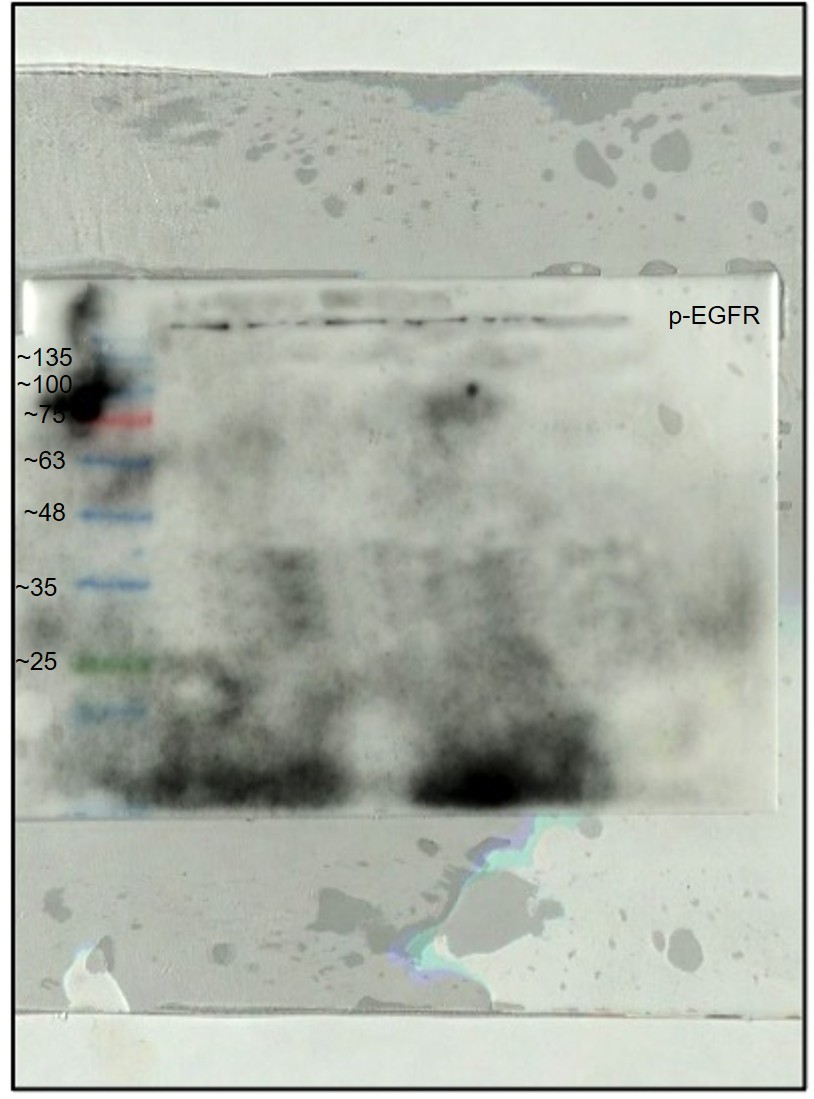

Supplement: Supplementary file 1 [file biomolecules-15-01410-s001.zip › Figure 8B_p-EGFR_Batch 2.jpg]

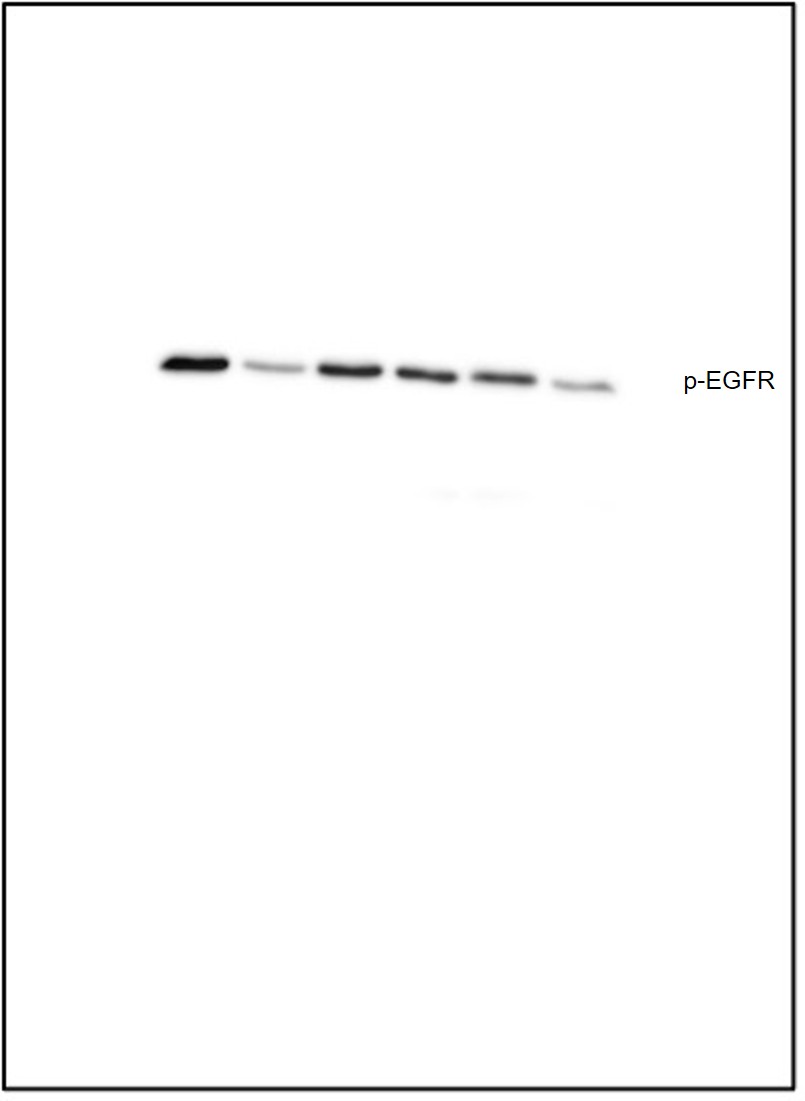

Supplement: Supplementary file 1 [file biomolecules-15-01410-s001.zip › Figure 8B_p-EGFR_Batch 3.jpg]
